# Development of 1,3,4-Oxadiazole Derived Antifungal Agents and Their Application in Maize Diseases Control

**DOI:** 10.3389/fpls.2022.912091

**Published:** 2022-05-04

**Authors:** Lin Yao, Guanghua Zhang, Lili Yu, Shaojing Liu, Xiaoku Wang, Tao Fan, Hui Kang, Wenzhi Feng

**Affiliations:** ^1^Shaanxi Key Laboratory of Chemical Additives for Industry, Shaanxi University of Science and Technology, Xi’an, China; ^2^College of Pharmacy, Xi’an Medical University, Xi’an, China

**Keywords:** oxadiazole derivatives, antifungal pesticides, maize diseases, plant fungicides, crop protection, crop health

## Abstract

Maize is an important food crop and its fungal disease has become a limiting factor to improve the yield and quality of maize. In the control of plant pathogens, commercial fungicides have no obvious effect on corn diseases due to the emergence of drug resistance. Therefore, it is of great significance to develop new fungicides with novel structure, high efficiency, and low toxicity to control maize diseases. In this paper, a series of 1,3,4-oxadiazole derivatives were designed and synthesized from benzoyl hydrazine and aromatic aldehydes through condensation and oxidation cyclization reaction. The antifungal activity of oxadiazole derivatives against three maize disease pathogens, such as *Rhizoctonia solani* (*R. solani*), *Gibberella zeae* (*G. zeae*), and *Exserohilum turcicum* (*E. turcicum*), were evaluated by mycelium growth rate method *in vitro*. The results indicated that most of the synthesized derivatives exhibited positive antifungal activities. Especially against *E. turcicum*, several compounds demonstrated significant antifungal activities and their *EC*_50_ values were lower than positive control carbendazim. The *EC*_50_ values of compounds 4k, 5e, and 5k were 50.48, 47.56, 32.25 μg/ml, respectively, and the carbendazim was 102.83 μg/ml. The effects of active compounds on *E. turcicum* microstructure were observed by scanning electron microscopy (SEM). The results showed that compounds 4k, 5e, and 5k could induce the hyphae of *E. turcicum* to shrink and collapse obviously. In order to elucidate the preliminary mechanism of oxadiazole derivatives, the target compounds 5e and 5k were docked with the theoretical active site of succinate dehydrogenase (SDH). Compounds 5e and 5k could bind to amino acid residues through hydrophobic contact and hydrogen bonds, which explained the possible mechanism of binding between the inhibitor and target protein. In addition, the compounds with antifungal activities had almost no cytotoxicity to MCF-7. This study showed that 1,3,4-oxadiazole derivatives were worthy for further attention as potential antifungal agents for the control of maize diseases.

## Introduction

Plant diseases, especially crop diseases are one of the major agricultural disasters, which cause huge losses to agricultural production every year. Around 70–80% of crop diseases are caused by plant pathogenic fungi. As the main source of crop infections, these fungi can cause a variety of plant diseases, resulting in crop yield and quality reduction (Bebber and Gurr, 2015; Fones et al., 2020; Nazarov et al., 2020). Maize (*Zea mays* L.) is one of the most important crops for food, feed, and energy throughout the world, and is grown in many countries and regions (Waheed et al., 2020; Wang et al., 2021a,b). The main diseases in maize are northern leaf blight (*Exserohilum turcicum*), southern corn rust (*Puccinia polysora*), fusarium head blight (*Gibberella zeae*), corn sheath blight (*Rhizoctonis solani*), gray leaf spot (*Cercospora zeae-maydis*), etc. (Van Rensburg et al., 2016; Hao et al., 2020; Silva et al., 2021). Among them, the northern corn leaf blight caused by *E. turcicum* is one of the most common maize diseases worldwide (Abdelsalam et al., 2022; De Rossi et al., 2022). These diseases are widespread in the world’s major corn producing areas. Because of their wide impact area and great economic loss, they have attracted the attention of domestic and foreign researchers. These diseases are also the most important threat to corn production in China and seriously hinder the stable development of corn production (Hao et al., 2020; Wang et al., 2021b). The most cost-effective and environmentally sustainable way to control plant diseases is to use tolerant or resistant cultivars. However, a single cultivar usually cannot resist all diseases in practice. Chemical control is still one of the most feasible technologies to ensure production efficiency (De Rossi et al., 2022). In order to control diseases, many chemical fungicides have been selected and used for crop protection (Ma et al., 2020). With the widespread application of fungicides in the control of crop diseases, the resistance of plant pathogens to existing fungicides has increased significantly, leading to the unsatisfactory control effect of current commercial fungicides on corn diseases (Leroux and Walker, 2013; Yoon et al., 2013; Shao et al., 2021). Therefore, it is particularly important to develop new fungicides with unique structure, high activity, and low toxicity to effectively control maize diseases.

Heterocyclic compounds have been the focus of research because of their high specificity, high activity, low dose, and low toxicity (Lamberth, 2013). Among them, azole, such as pyrazole, imidazole, oxazole, and other important members of five-member heterocyclic system, exist in many products with physiological active structure, are widely used in biology, medicine, industry and other fields, and show unique properties (Küçükgüzel and Şenkardeş, 2015; Lipunova et al., 2015; Guerrero-Pepinosa et al., 2021; Rulhania et al., 2021; Parikh et al., 2022). Many azole derivatives had become important drugs, which can be used in the treatment of antifungal (Lipunova et al., 2015; Wei et al., 2018; Guerrero-Pepinosa et al., 2021; Zhang et al., 2022), antitumor (Küçükgüzel and Şenkardeş, 2015; Silva-Ortiz et al., 2016; Titi et al., 2020; Balaraju et al., 2021; Oskuei et al., 2021), antituberculosis (Xu et al., 2017; Fan et al., 2018; Negalurmath et al., 2019), hypoglycemic (Al-Harbi et al., 2013), anti-inflammatory (Selvam et al., 2014), and other diseases, especially in pesticides, often used as fungicides and herbicides (Price et al., 2015; Shevaldina et al., 2019). Among them, oxazole derivatives were often used in the structural design of active molecules due to their biological activities such as antifungal, antiparasitic (Yamamuro et al., 2015; Adeyemi et al., 2021), antitumor (Balaraju et al., 2021), antituberculosis (Negalurmath et al., 2019), antimalaria (Verma et al., 2018), hypoglycemia (Taha et al., 2015), and antioxidation (Hejazi et al., 2019).

Therefore, in order to solve the problems of maize diseases caused by plant pathogenic fungi, a series of 1,3,4-oxadiazole derivatives were prepared using oxadiazole as lead compound. The derivatives were used to control three main plant pathogenic fungi (such as *E. turcicum*, *G. zeae*, and *R. solani*) causing maize diseases, and their biological activities against pathogens were evaluated in order to screen out compounds with higher activities. This study can provide candidate compounds for the prevention and control of maize disease.

## Materials and Methods

### Materials and Chemicals

All reagents and solvents required for the synthesis of compounds were analytical or chemical pure, purchased from commercial suppliers and used without further purification. The reactions were observed and monitored by thin layer chromatography (TLC) under UV light (254 nm) using silica gel precoated plate (GF_254_, 0.25 mm). About 200 ~ 300 mesh silica gel was used for column chromatography separation and purification (Qingdao Haiyang Co., Ltd., Qingdao, Shandong, China). All compounds were eluted using a mixture of petroleum ether (PE), dichloromethane (DCM), and ethyl acetate (EA). The melting points (m.p.) were measured by JHX-4B micro-melting point apparatus (Shanghai JIAHANG Instrument Co., Ltd) and were uncorrected. ^1^H-NMR and ^13^C-NMR spectra were recorded on Bruker Advance III 400 MHz nuclear magnetic resonance spectrometer (Bruker Company, United States) using CDCl_3_ or DMSO-*d*_6_ as solvent and tetramethylsilane (TMS) as internal standard. The chemical shifts for the NMR spectra were reported in δ ppm. Peak multiplicities were expressed as singlet (s), doublet (d), triplet (t), quartet (q), and multiplet (m).

### Fungi

Three plant pathogenic fungi species *R. solani*, *G. zeae*, and *E. turcicum* were provided by College of Plant Protection, Southwest University, Chongqing, China. These pathogens can cause corn sheath blight, fusarium head blight, and northern corn leaf blight. The fungi were removed from the storage tube and incubated in a petri dish with potato dextrose agar (PDA) medium at 25°C for 1 week. The activated fungal mycelia were used in antifungal experiments.

### General Procedures of 1,3,4-Oxadiazole Derivatives

#### General Synthetic Procedure for Compounds 4a–4l

*p*-Methoxy benzoyl hydrazide (166 mg, 1 mmol) and *p*-methoxy benzaldehyde (136 mg, 1 mmol) were dissolved in anhydrous EtOH (20 ml), and refluxed at 80°C for 6–10 h. The progress of reaction was monitored by TLC. The reaction was stopped until the initial material disappeared. After the reaction mixture was cooled, the solvent was concentrated under reduced pressure to obtain the first step product *N*-acylaldehyde hydrazone, which was directly used for the next reaction (Majji et al., 2014; Polkam et al., 2017).

Anhydrous K_2_CO_3_ (276 mg, 2 mmol) and I_2_ (26 mg, 0.1 mmol) were added to *N*-acylaldehyde hydrazine and dissolved in 3 ml DMSO. The reaction mixture was refluxed at 60–70°C for 10–12 h. H_2_O_2_ (30% solution in water, 0.45 ml, 4 mmol) was added to the reaction solution nine times in 3 h during the reaction. After the reaction stopped, it was quenched with 5% sodium thiosulfate solution (5 ml). The reaction mixture was extracted with ethyl acetate (3 × 15 ml), the combined organic layer was washed with saturated sodium chloride (1 × 3 ml) and dried over anhydrous Na_2_SO_4_. The organic phase was then filtered and concentrated under reduced pressure to remove the solvent. The product was separated and purified by silica gel column chromatography with a solvent mixture of petroleum ether/dichloromethane/ethyl acetate (4:1:1, *v*/*v*) as mobile phase, and the compound 4a was obtained after vacuum drying for 12 h. Compounds 4b–4l were synthesized by the same method. The synthetic routes and structures of compounds were shown in Scheme 1.

**Scheme 1:** General synthetic procedure of 1,3,4-oxadiazole derivatives (4a–4l, 5a–5l, and 6a–6f).

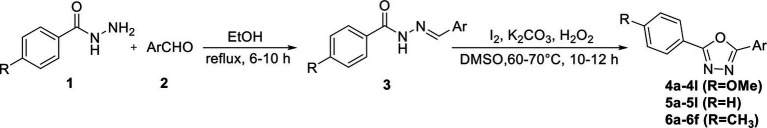					
Compound	R	Ar	Compound	R	Ar
4a	OMe	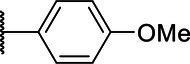	5d	H	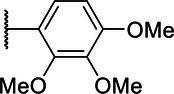
4b	OMe	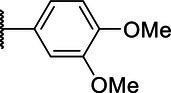	5e	H	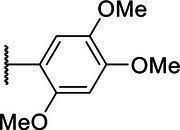
4c	OMe	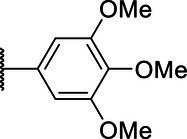	5f	H	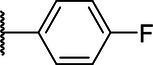
4d	OMe	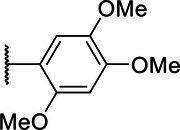	5g	H	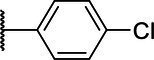
4e	OMe	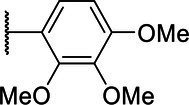	5h	H	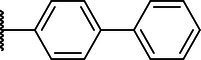
4f	OMe	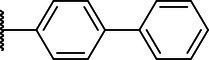	5i	H	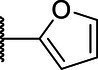
4g	OMe	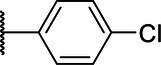	5j	H	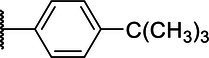
4h	OMe	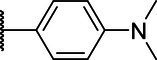	5k	H	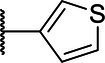
4i	OMe	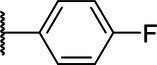	5l	H	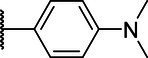
4j	OMe	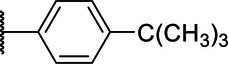	6a	Me	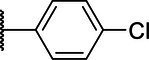
4k	OMe	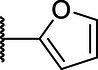	6b	Me	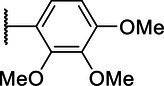
4l	OMe	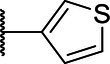	6c	Me	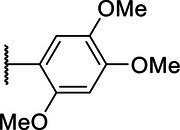
5a	H	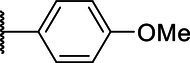	6d	Me	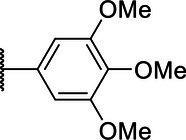
5b	H	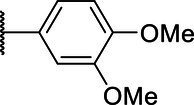	6e	Me	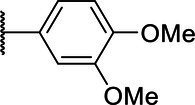
5c	H	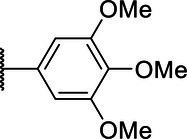	6f	Me	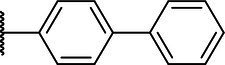

#### General Synthetic Procedure for Compounds 5a–5l

Substitute *p*-methoxy benzoyl hydrazide with benzoyl hydrazide, and the synthetic methods and dosage were the same as general synthetic procedure for compounds 4a–4l to obtain compounds 5a–5l.

#### General Synthetic Procedure for Compounds 6a–6f

Substitute *p*-methoxy benzoyl hydrazide with *p*-methylbenzoyl hydrazine, and the synthetic methods and dosage were the same as general synthetic procedure for compounds 4a–4l to obtain compounds 6a–6f.

### Antifungal Activity Assay *in vitro*

#### Antifungal Activity Assay

The antifungal activity of oxadiazole derivatives and the positive control carbendazim were evaluated against three plant pathogenic fungi by the mycelium growth rate method (Fan et al., 2020; Yang et al., 2022). Firstly, the compounds were dissolved in DMSO and mixed with sterile molten PDA to obtain medicated medium with final concentration of 50 μg/ml. The mixture was poured into disposable petri plates (6 cm), and 3 mm mycelial disks were taken from the edge of hyphae and inoculated on the medicated medium. Three mycelial disks were placed evenly in each petri dish and then were cultured at 30°C PDA containing corresponding concentration of DMSO was used as blank control. The mycelial growth diameters were measured and the data were recorded when the control fungi grew and covered 3/4 of the petri dish. The diameters (mm) of inhibition zones were measured by the cross-bracketing method. The growth inhibition rates were calculated according to the following formula:


$$\mathrm{Mycelial growth inhibiton}\left(\%\right)=\frac{\left[\left(C-d\right)-\left(T-d\right)\right]}{C-d}\times 100$$

where *d* represent the diameter of the mycelial disks (3 mm), *C* and *T* represents growth diameters of fungi on untreated PDA and treated PDA, respectively.

The *EC*_50_ values of the compounds with high inhibitory activity were determined. The target compounds were dissolved in DMSO and prepared to obtain a series of concentrations (100.00, 50.00, 25.00, 12.50, and 6.25 μg/ml). Three parallel controls were established for each concentration, with DMSO as blank control and carbendazim as positive control (Shivamogga Nagaraju et al., 2020). The log value of drug concentration (lg*c*) was taken as the independent variable (*x*), and average inhibition rate was taken as the dependent variable (*y*). The *EC*_50_ values of the target compound were calculated by regression equation.

#### Scanning Electron Microscopy Observations

In order to investigate the effects of active compounds on *E. turcicum* microstructure, the Scanning electron microscopy (SEM) observations were performed according to the literature method (Li et al., 2019). First, *E. turcicum* at logarithmic growth stage (1.5 ml) was centrifuged at 7,000 rpm for 1–2 min to remove the supernatant, then washed with PBS (3 × 1 ml) and suspended in 1.5 ml PBS. Compounds 4k, 5e, and 5k dissolved in DMSO were added to the suspension to make the final concentrations 50.0, 50.0, and 25.0 μg/ml, respectively, and cultured at 30 ± 1°C and 180 rpm for 24–48 h. DMSO was used as blank control, and three parallel controls were established for each compound. Afterward, the hyphae in three parallel samples were combined after washing with PBS (3 × 1 ml) and treated by 1 ml 2.5% glutaraldehyde at 4°C overnight. After removing the fixative, the samples were dehydrated with 1 ml gradient ethanol series (10, 30, 50, 70, 90 and 100%) with 5 min for each time and replaced with 100% tert-butanol twice. Finally, the treated samples were observed by scanning electron microscope (TESCAN MIRA LMS, Czech Republic) after dried in cryogenic freeze-drying device and gold-sprayed.

### Cytotoxicity Assay

The cytotoxic activities of oxadiazole derivatives against MCF-7 were evaluated by MTT method (Mi et al., 2021). The tumor cells were cultured to logarithmic phase and inoculated into 96-well flat-bottom culture plates with the density of 1.0 × 10^5^ cells. The cells were incubated at 37°C under 5% CO_2_ atmosphere for 24 h. The different concentrations of compounds (1.5625, 6.25, 25, and 100 μM) were added into the 96-well plates. About 1% DMSO was used as blank control, and three replicates were set for each concentration. Then, the cells were cultured for 24 h. Subsequently, MTT/PBS solution (1:9, *v*/*v*) was added to each well plate. Around 4 h later, the absorbance at 490 nm was detected by ELISA, and the cell viability was calculated by following equation:


$$\mathrm{Cell viability}\;\left(\%\right)=\left(OD_{treatment}/OD_{control}\right)\times 100$$

where *OD_treatment_* represent the absorbance of samples and *OD_control_* represent the absorbance of blank control.

### Molecular Docking

In order to evaluate the binding modes between target compounds (5e and 5k) with target enzymes succinate dehydrogenase (SDH; Yan et al., 2019; Yu et al., 2021), the molecular docking was conducted using Surflex-Dock program in the Sybyl-X software (version 2.1.1), and interaction analyzed using Ligplot^+^ client (version 2.2.4) and Pymol client. The crystal structure of SDH was downloaded from the Protein Data Bank (PDB code: 2FBW). Ligands and H_2_O in the crystal structure of protein were deleted, and hydrogen atoms and charges were added using the AMBER7 FF99 method. The structures of compounds 5e and 5k were optimized by adding charge (Gasteigere−Hückel) and Tripos force field.

## Results and Discussion

### Chemical Synthesis

The synthetic routes of intermediates and oxadiazole derivatives were performed as illustrated in Scheme 1. Firstly, *p*-methoxy benzoyl hydrazide (1) and aromatic aldehyde (2) were condensed in EtOH by condensation reaction to obtain the intermediate *N*-acylaldehyde hydrazone (3). Then, the intermediate three were oxidized and cyclized by H_2_O_2_ in I_2_ and K_2_CO_3_ to obtain 2, 5-disubstituted-1,3,4-oxadiazole derivatives (4a–4l). Similarly, compound 1 was replaced with benzoyl hydrazine or *p*-methylbenzoyl hydrazine, and oxadiazole derivatives 5a–5l and 6a–6f were obtained using the same experimental method. The yields of these compounds ranged from 47 to 90%. The purity and structure of target compounds were confirmed by TLC, melting point determination, ^1^H-NMR and ^13^C-NMR. The NMR Spectra of oxadiazole derivatives are shown in the supporting materials. All NMR data were consistent with the structure of the target compounds.

### Antifungal Activity *in vitro* and Structure–Activity Relationship

#### Antifungal Activity *in vitro*

**Figure 1:**
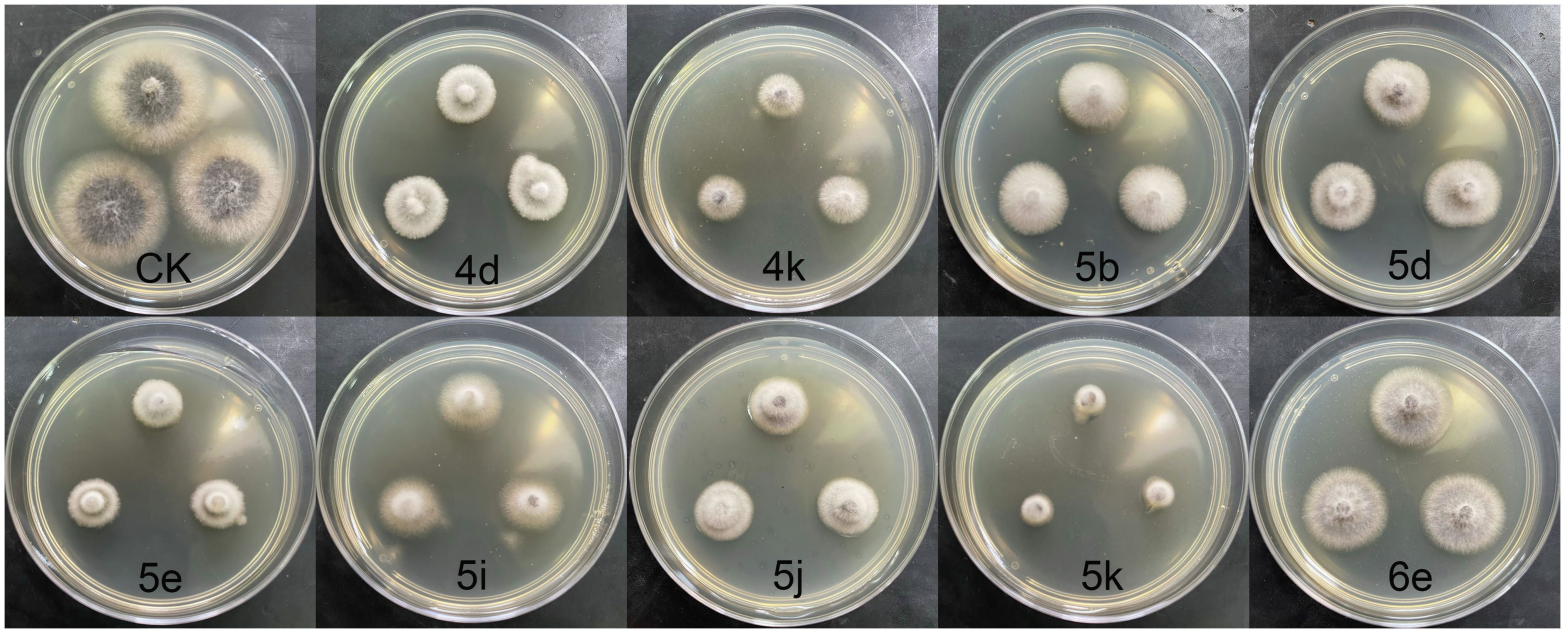
Antifungal activities of active compounds against *Exserohilum turcicum*.

The antifungal activities of oxadiazole derivatives and the positive control carbendazim were evaluated against three plant pathogenic fungi causing maize diseases by the mycelium growth rate method, including *R. solani*, *G. zeae*, and *E. turcicum*. Firstly, the antifungal activities of 29 compounds were determined at 50 μg/ml *in vitro*. As shown in Table 1, the results indicated that compound 5k had superior inhibitory activities against three tested fungi. The inhibitory rates of compound 5k were 50.93, 59.23, and 70.56% against *R. solani*, *G. zeae*, and *E. turcicum*, respectively. Compounds 4k, 5c, and 5k had significant inhibitory effects against *G. zeae*, and the inhibitory rates were more than 50%, among them, 4k showed the highest activity with an inhibitory rate of 70.46%, followed by 5c with an inhibitory rate of 55.52%. Among the three pathogens, oxadiazole derivatives had the most significant activity against *E. turcicum*. The antifungal activities of compounds 4k, 5b, 5d, 5e, 5i, 5k, and 6e were all greater than 50%, and compound 5k had the highest inhibition rate of 70.56%, followed by 4k with 62.19% (Figure 1).

**Table 1:** Antifungal activities of oxadiazole derivatives *in vitro* at 50 μg/ml.^a^

Compound	Inhibition rate^b^ (%)		
	*Rhizoctonia solani*	*Gibberella zeae*	*Exserohilum turcicum*
4a	0.00	2.37 ± 3.35	21.02 ± 1.93
4b	5.05 ± 0.58	26.16 ± 0.98	14.50 ± 0.22
4c	3.13 ± 0.56	0.00	0.00
4d	23.13 ± 0.62	32.04 ± 3.11	44.52 ± 1.53
4e	6.31 ± 1.68	0.00	18.54 ± 1.49
4f	0.44 ± 0.98	0.00	4.65 ± 2.37
4g	0.00	5.67 ± 2.65	6.52 ± 1.08
4h	0.00	14.98 ± 1.14	14.17 ± 2.14
4i	0.44 ± 1.74	0.38 ± 5.54	14.02 ± 4.35
4j	0.00	13.01 ± 4.15	7.77 ± 3.69
4k	26.51 ± 1.25	70.46 ± 3.85	62.19 ± 2.67
4l	12.00 ± 1.94	4.23 ± 2.31	41.40 ± 2.45
5a	0.00	0.00	21.54 ± 1.86
5b	24.76 ± 1.88	15.81 ± 6.87	51.25 ± 0.18
5c	31.62 ± 2.41	55.52 ± 6.43	36.97 ± 1.20
5d	17.34 ± 1.96	17.98 ± 5.89	53.31 ± 4.17
5e	22.22 ± 1.27	35.41 ± 5.05	59.92 ± 3.84
5f	3.00 ± 0.10	20.44 ± 4.03	27.91 ± 3.45
5g	5.47 ± 2.14	23.14 ± 0.82	22.45 ± 3.87
5h	0.00	14.44 ± 6.41	4.13 ± 3.20
5i	41.22 ± 1.33	37.03 ± 2.12	55.71 ± 3.16
5j	22.67 ± 2.75	38.60 ± 0.76	44.52 ± 1.70
5k	50.93 ± 2.84	59.23 ± 2.64	70.56 ± 2.57
6a	0.00	0.00	0.52 ± 1.70
6b	3.15 ± 1.27	3.39 ± 0.42	5.73 ± 1.12
6c	2.89 ± 1.18	9.98 ± 6.11	18.03 ± 5.66
6d	0.00	0.00	14.55 ± 0.24
6e	30.38 ± 2.55	30.18 ± 4.11	53.55 ± 2.17
6f	4.70 ± 1.41	0.00	0.00

a *Rhizoctonia solani* (*R. solani*), *Gibberella zeae* (*G. zeae*), and *Exserohilum turcicum* (*E. turcicum*).

b Values are the mean ± SD of three replicates.

In order to further research the antifungal activities of oxadiazole derivatives, the *EC*_50_ values of compounds with higher inhibition rate were determined, and the results were illustrated in Table 2. Compound 5k showed high antifungal activities against three pathogens. In addition, oxadiazole derivatives had obvious antifungal activity against *E. turcicum*. The inhibitory activities of 4d, 4k, 5b, 5e, 5i, 5j, and 5k were better than positive control carbendazim. Especially, the *EC*_50_ values of 4k, 5e, 5i, and 5k were 50.48, 47.56, 47.05, and 32.25 μg/ml, respectively, and the positive control carbendazim was 102.83 μg/ml. Among them, 5k showed the strongest activity, it was about 3-fold more active than carbendazim. These results indicate that oxadiazole derivatives have potential as control drugs for maize diseases.

**Table 2:** Antifungal activities with *EC*_50_ of the target compounds against three plant pathogenic fungi *in vitro*.

Fungal species	Compound	Regression equation^a^	*EC*_50_ (μg/ml)	*R* ^2^
*Rhizoctonia solani*	5i	*y* = 1.7176*x* + 1.8239	63.04	0.8844
	5k	*y* = −0.4371*x* + 3.2440	47.43	0.9332
	Carbendazim^b^	*y* = 4.8410*x* + 1.7872	1.23	0.8419
*Gibberella zeae*	4k	*y* = 0.0758*x* + 2.4399	104.28	0.9193
	5i	*y* = 2.8069*x* − 0.3231	78.79	0.9999
	5k	*y* = −0.0755*x* + 2.8019	64.78	0.9141
	Carbendazim	*y* = 3.8018*x* + 0.9276	19.58	0.9974
*Exserohilum turcicum*	4d	*y* = 3.3590*x* + 0.8333	93.15	0.9694
	4k	*y* = 0.7614*x* + 2.4888	50.48	0.9298
	5b	*y* = 2.2442*x* + 1.3916	95.57	0.8648
	5d	*y* = 4.0792*x* + 0.4099	176.45	0.8721
	5e	*y* = 2.9551*x* + 1.2192	47.56	0.9784
	5i	*y* = 0.7322*x* + 2.5516	47.05	0.9955
	5j	*y* = 1.0486*x* + 2.0898	77.77	0.9763
	5k	*y* = 2.0559*x* + 1.9516	32.25	0.9779
	6e	*y* = 3.8762*x* + 0.3870	801.73	0.9787
	Carbendazim	*y* = 0.0875*x* + 2.4415	102.83	0.9497

a *x* represents the logarithm of molar concentration (*lgc*) and *y* represents the average inhibition rate.

b Commercial fungicide carbendazim as positive control.

#### Structure–Activity Relationship

Based on the antifungal activity data (Tables 1, 2), the structure–activity relationship (SAR) of oxadiazole derivatives were analyzed. Compounds 5i and 5k had superior inhibitory effects on the three tested plant pathogenic fungi. Their structures contain aromatic five-membered heterocycles, in which 5i was furan ring and 5k was thiophene ring. It shows that the introduction of aromatic five-membered heterocycles may promote the improvement of antifungal activity. Such as compound 4k contained furan ring and exhibited higher inhibition against the other two fungi except *R. solani*.

However, not all oxadiazole derivatives containing five-membered heterocycles had significant antifungal activity. For example, compound 4l contained thiophene ring, but its inhibitory activities against *R. solani* and *G. zeae* were inferior than 5i and 5k. This was probably due to the presence of methoxide group on the other side of oxazole ring, which weakens hydrophobic force of thiophene. Among the three series of compounds, the activity of series 5 was stronger than series 4 and series 6. These results inferred that most of highly active compounds were not substituted or only one substituted on the oxadiazole-linked aromatic ring. The simultaneous attachment of substituents to 2, 5-position aromatic groups of oxadiazole may have an adverse effect on its antifungal activity. Other groups, such as the position and number of methoxy group and the presence of halogen, had no obvious influence on antifungal activity.

### Effects on Mycelial Morphology of Plant Pathogenic Fungi

**Figure 2:**
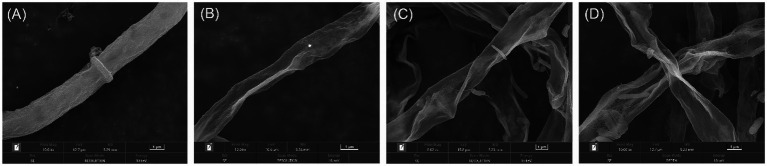
Scanning electron microscopy (SEM) micrographs of *Exserohilum turcicum* hyphae. **(A)** Blank control, **(B)** Treated by 4k at 50 μg/ml, **(C)** Treated by 5e at 50 μg/ml, and **(D)** Treated by 5k at 25 μg/ml.

The morphological changes of *E. turcicum* mycelia treated by active compounds were observed under SEM. Three compounds 4k, 5e, and 5k were selected according to the results of their antifungal activity and structures. The results were shown in Figure 2. The edge of the blank control hyphae treated by DMSO (Figure 2A) was uniform, and the surface of hyphae was regular and relatively smooth. However, after treated by 4k, 5e, and 5k (Figures 2B–D), the morphology of *E. turcicum* hyphae were curved, with obvious shrinkage and collapse on the surface and roughness on the outer walls. These results conjectured that compounds 4k, 5e, and 5k may disrupt the morphology and structure of mycelium and lead to cytoplasmic outflow. The SEM results further confirmed that the inhibitory effects of oxazole derivatives against plant pathogens were consistent with the experimental results *in vitro*.

### Cytotoxicity

**Figure 3:**
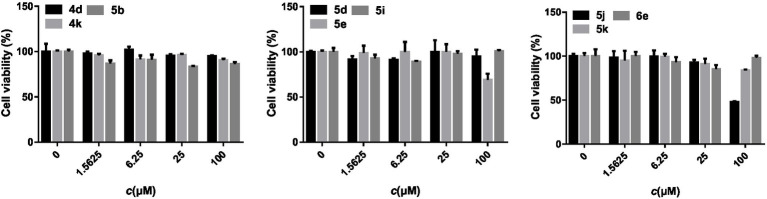
Cytotoxic activity results of target compounds.

The cytotoxic activities of several oxadiazole derivatives against MCF-7 cell line were determined by MTT assay (Gewirtz, 1999), and the results were shown in Figure 3. Specifically, compounds 4d, 4k, 5b, 5d, 5i, 5k, and 6e demonstrated no cytotoxicity at all tested concentrations, with survival rates greater than 80% at 100 μmol/L. Only 5e and 5j manifested weaker inhibition, the survival rates were 69 and 48%, respectively. The cell viability of two compounds decreased at the concentration of 100 μmol/L, but the toxicities were not significant. Compounds with superior antifungal activities, such as 4k, 5e, 5i, and 5k, showed no cytotoxic activities. These results can preliminarily show that most oxadiazole derivatives had no potential damage on mammalian cells. However, the cytotoxicity against human normal liver cells remains to be further verified.

### Molecular Docking Results

**Figure 4:**
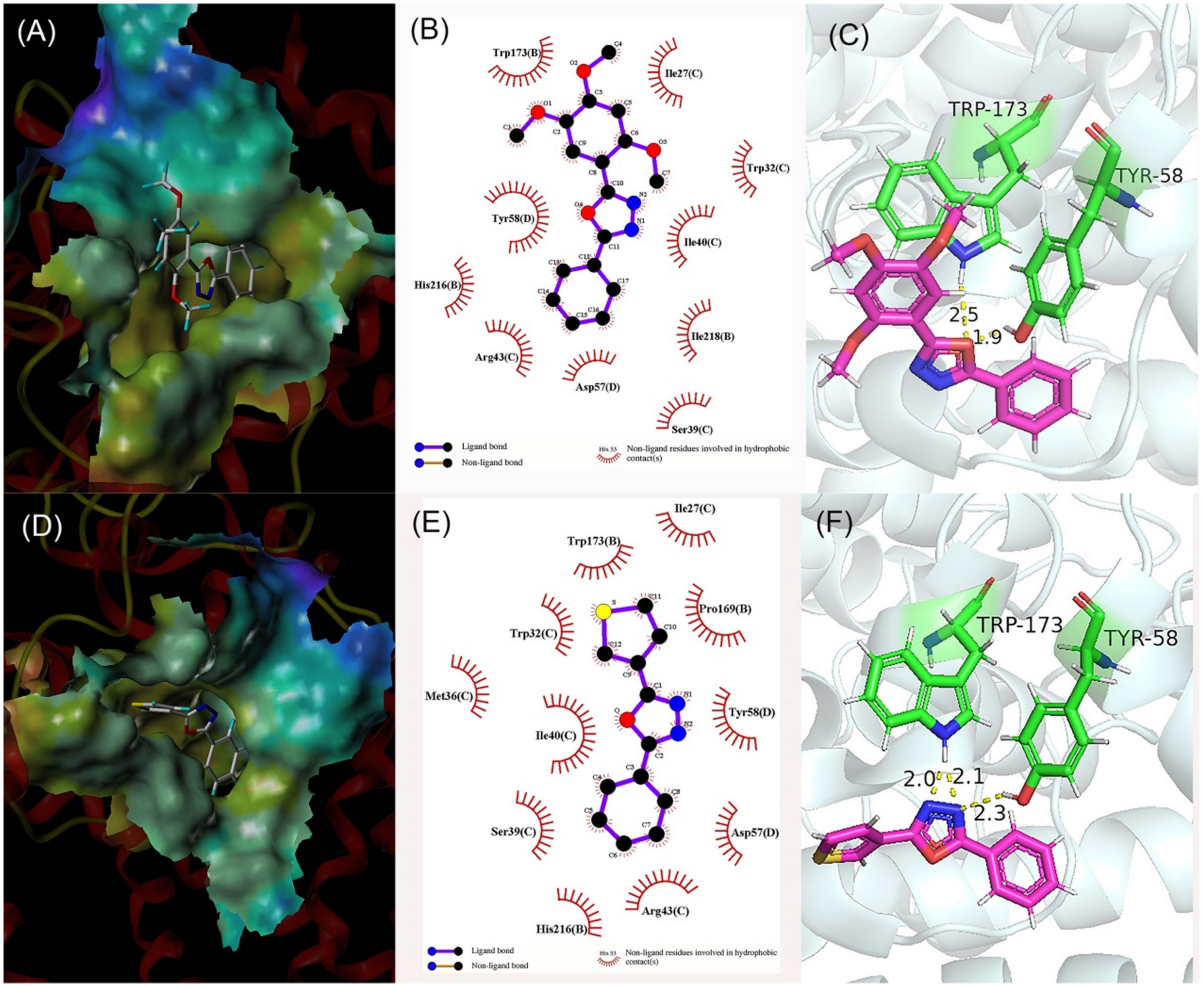
Molecular docking results of compound 5e **(A–C)** and compound 5k **(D–F)**. **(A,D)** Predicted binding mode of compound within active pocket; **(B,E)** Predicted interactions between compound and amino acids; and **(C,F)** Binding modes of compound and amino acids.

In order to elucidate the preliminary mechanism of antifungal activities of these compounds, the target compounds were docked with the theoretical active site of SDH by SYBYL-X. SDH was one of the important molecular targets for developing new fungicides. Compounds 5e and 5k were selected for molecular docking because of their high antifungal activities and different structural units. Molecular docking results were shown in Figure 4.

In Figures 4A,B, compounds 5e was bound in the active pocket of SDH, and can produce hydrophobic contact with 10 amino acid residues, including Trp173, Tyr58, His216, Arg43, Asp57, Ser39, Ile218, Ile40, Trp32, and Ile27. Meanwhile, compound 5e can form two hydrogen bonds with amino acid residues Trp173 and tyr58 through the oxygen atom on the oxadiazole ring, which was crucial for the binding between the inhibitor and SDH. The hydrogen bond lengths were 1.9 Å and 2.5 Å, respectively (Figure 4C). In Figures 4D,E, compounds 5k can produce hydrophobic contact with 11 amino acid residues, including Trp173, Trp32, Met36, Ile40, Ser39, His216, Arg43, Asp57, Tyr58, Pro169, and Ile27, and can form three hydrogen bonds with amino acid residues Trp173 and Tyr58 through nitrogen atoms on the oxadiazole ring. The hydrogen bond lengths were 2.0, 2.1, and 2.3 Å, respectively (Figure 4F).

It can be seen that the number of residues and hydrogen bonds between compound 5k and SDH are greater than 5e. The presence of 2,4,5-methoxyl group in the 5e structure leads to its repulsion with amino acid residues, which may be the reason for its relative weaker antifungal potency. Molecular docking results showed that hydrophobic contact and hydrogen bond formation could promote the binding between compound and SDH, and improve the antifungal activity.

## Conclusion

Maize is an important food crop and its fungal disease has become a limiting factor to improve the yield and quality of maize. In the control of plant pathogens, commercial fungicides have no obvious effect on corn diseases due to the emergence of drug resistance. Therefore, it is of great significance to develop new fungicides with novel structure, high efficiency, and low toxicity to control maize diseases. In this paper, a series of 1,3,4-oxadiazole derivatives (4a–4l, 5a–5l, and 6a–6f) were designed and synthesized from three kinds of benzoyl hydrazine and various aromatic aldehydes. The antifungal activities of oxadiazole derivatives against *R. solani*, *G. zeae*, and *E. turcicum* which caused maize diseases were evaluated. The results indicated that compound 5k had superior inhibitory activities against three tested fungi. Especially against *E. turcicum*, the inhibitory activity of 5k exceeded carbendazim, and their *EC*_50_ values were 32.25 and 102.83 μg/ml, respectively. The compounds with antifungal activities had almost no cytotoxicity to MCF-7, which could provide a basis for further cytotoxicity study on normal human liver cells. The preliminary mechanism research indicated that compounds 4k, 5e, and 5k could induce the obvious shrinkage and collapse of *E. turcicum* mycelia. Molecular docking results further revealed the interaction between receptor and ligand. Compounds 5e and 5k could bind to amino acid residues through hydrophobic contact and hydrogen bonds, which explained the possible mechanism of binding between the inhibitor and target protein. Consequently, oxadiazole derivatives have the potential as plant fungicides to control maize diseases.

## Data Availability Statement

The original contributions presented in the study are included in the article/Supplementary Material, further inquiries can be directed to the corresponding author.

## Author Contributions

LYa, GZ, and LYu contributed to the conception and design of this study. SL characterized the structure of the derivatives. XW measured the cytotoxic activity of the derivatives. TF completed the molecular docking. HK and WF completed the determination of antifungal activities and the statistics of some databases. LYa finished the first draft. All authors contributed to the article and approved the submitted version.

## Funding

This work was supported financially by the Special Scientific Research Program of Shaanxi Education Department (No. 20JK0892), the Natural Science Basic Research Project of Shaanxi Science and Technology Department (No. 2022JM-492), and the Science and Technology Innovation Team of Xi’an Medical University (No. 2021TD07).

## Conflict of Interest

The authors declare that the research was conducted in the absence of any commercial or financial relationships that could be construed as a potential conflict of interest.

## Publisher’s Note

All claims expressed in this article are solely those of the authors and do not necessarily represent those of their affiliated organizations, or those of the publisher, the editors and the reviewers. Any product that may be evaluated in this article, or claim that may be made by its manufacturer, is not guaranteed or endorsed by the publisher.
