# Supplementary material for: Development of 1,3,4-Oxadiazole Derived Antifungal Agents and Their Application in Maize Diseases Control
Source: Front Plant Sci. 2022 May 4;13:912091. doi: 10.3389/fpls.2022.912091 (PMC9114798; doi:10.3389/fpls.2022.912091)
Supplement: Supplementary file 1 [file Data_Sheet_1.docx]

Supplementary Material

**Contents**

Page

1. Synthesis and spectral data of compounds **4a–4l**. S2

2. Synthesis and spectral data of compounds **5a–5l**. S4

3. Synthesis and spectral data of compounds **6a–6f**. S6

4. Copies of NMR spectra of 1, 3, 4-Oxadiazole Derivatives. S8

# Synthesis and spectral data of compounds 4a–4l.

**4a:**

2,5-Bis(4-methoxyphenyl)-1,3,4-oxadiazole(**4a**): White solid, yield 75%; m.p.:177.1-178.2 °C; ^1^H-NMR (400 MHz, CDCl_3_) δ 8.10-8.08(d, 4*H*, Ar-*H*), 7.06-7.04(d, 4*H*, Ar-*H*), 3.92(s, 6*H*, -OC*H*_3_); ^13^C-NMR (101 MHz, CDCl_3_) δ 164.08, 162.18, 128.58, 116.60, 114.46, 55.48.

**4b:**

2-(3,4-Dimethoxyphenyl)-5-(4-methoxyphenyl)-1,3,4-oxadiazole(**4b**): White solid, yield 81%; m.p. 156.8-158.1 °C; ^1^H-NMR (400 MHz, DMSO) δ 8.07-8.05(d, 2*H*, Ar-*H*), 7.70-7.67(q, 1*H*, Ar-*H*), 7.58(d, 1*H*, Ar-*H*), 7.18-7.14(m, 3*H*, Ar-*H*), 3.89-3.86(d, 9*H*, -OC*H*_3_); ^13^C-NMR (101 MHz, CDCl_3_) δ 164.17, 164.08, 162.20, 151.85, 149.30, 128.58, 120.22, 116.65, 116.50, 114.44, 111.04, 109.34, 56.15, 56.03, 55.46.

**4c:**

2-(4-Methoxyphenyl)-5-(3,4,5-trimethoxyphenyl)-1,3,4-oxadiazole(**4c**): White solid, yield 89%; m.p. 169.2-170.0 °C; ^1^H-NMR (400 MHz, CDCl_3_) δ 8.11-8.09(d, 2*H*, Ar-*H*), 7.36(s, 2*H*, Ar-*H*), 7.07-7.05(d, 2*H*, Ar-*H*), 4.00-3.92(t, 12*H*, -OC*H*_3_); ^13^C-NMR (101 MHz, CDCl_3_) δ 164.47, 164.05, 162.33, 153.66, 140.98, 128.69, 119.19, 116.37, 114.49, 104.08, 61.03, 56.41, 55.48.

**4d:**

2-(4-Methoxyphenyl)-5-(2,4,5-trimethoxyphenyl)-1,3,4-oxadiazole(**4d**): White solid, yield 62%; m.p. 147.0-147.8 °C; ^1^H-NMR (400 MHz, CDCl_3_) δ 8.10-8.07(t, 2*H*, Ar-*H*), 7.55(s, 1*H*, Ar-*H*), 7.06-7.03(q, 2*H*, Ar-*H*), 6.65(s, 1*H*, Ar-*H*), 4.01-4.00(d, 6*H*, -OC*H*_3_), 3.96(s, 3*H*, -OC*H*_3_), 3.91.(s, 3H, -OC*H*_3_); ^13^C-NMR (101 MHz, CDCl_3_) δ 163.03, 162.01, 161.08, 152.24, 151.77, 142.31, 127.59, 115.81, 113.39, 111.28, 103.52, 96.73, 56.01, 55.57, 55.10, 54.44.

**4e:**

2-(4-Methoxyphenyl)-5-(2,3,4-trimethoxyphenyl)-1,3,4-oxadiazole(**4e**): White solid, yield 61%; m.p.144.3-145.0 °C; ^1^H-NMR (400 MHz, CDCl_3_) δ 8.10-8.08(d, 2*H*, Ar-*H*), 7.79-7.77(d, 1*H*, Ar-*H*), 7.06-7.04(q, 2*H*, Ar-*H*), 6.85-6.83(d, 1*H*, Ar-*H*), 4.07(s, 3*H*, -OC*H*_3_), 3.97-3.91(t, 9*H*, -OC*H*_3_); ^13^C-NMR (101 MHz, CDCl_3_) δ 163.23, 161.52, 161.16, 155.50, 151.76, 142.08, 127.58, 123.91, 115.70, 113.46, 110.60, 106.82, 60.66, 60.07, 55.15, 54.44.

**4f:**

2-([1,1'-Biphenyl]-4-yl)-5-(4-methoxyphenyl)-1,3,4-oxadiazole(**4f**): White solid, yield 68%; m.p. 147.2-148.3 °C; ^1^H-NMR (400 MHz, CDCl_3_) δ 8.23-8.21(d, 2*H*, Ar-*H*), 8.14-8.12, (d, 2*H*, Ar-*H*) 7.79-7.77(d, 2*H*, Ar-*H*), 7.69-7.68(d, 2*H*, Ar-*H*), 7.54-7.50 (t, 2*H*, Ar-*H*) 7.45-7.44(d, 1*H*, Ar-*H*), 7.08-7.06(d, 2*H*, Ar-*H*), 3.93(s, 3*H*, -OC*H*_3_); ^13^C-NMR (101 MHz, CDCl_3_) δ 164.57, 164.08, 162.39, 144.30, 139.89, 129.01, 128.76, 128.17, 127.70, 127.31, 127.18, 122.85, 116.47, 114.55, 55.51.

**4g:**

2-(4-Chlorophenyl)-5-(4-methoxyphenyl)-1,3,4-oxadiazole(**4g**): White solid, yield 49%; m.p. 167.0-167.7 °C; ^1^H-NMR (400 MHz, CDCl_3_) δ 8.10-8.07(m, 4*H*, Ar-*H*), 7.54-7.52(d, 2*H*, Ar-*H*), 7.07-7.05(d, 2*H*, Ar-*H*), 3.92(s, 3*H*, -OC*H*_3_); ^13^C-NMR (101 MHz, CDCl_3_) δ 163.64, 162.28, 161.41, 136.72, 128.39, 127.70, 127.03, 121.52, 115.17, 113.51, 54.46.

**4h:**

4-(5-(4-Methoxyphenyl)-1,3,4-oxadiazol-2-yl)-*N,N*-dimethylaniline(**4h**): Yellow solid, yield 58%; m.p. 187.6-188.8 °C; ^1^H-NMR (400 MHz, CDCl_3_) δ 8.08-8.06(d, 2*H*, Ar-*H*), 8.00-7.98(d, 2*H*, Ar-*H*), 7.05-7.03(d, 2*H*, Ar-*H*), 6.81-6.79(d, 2*H*, Ar-*H*), 3.91(s, 3*H*, -OC*H*_3_) 3.09(s, 6H, -OC*H*_3_); ^13^C-NMR (101 MHz, CDCl_3_) δ 163.80, 162.43, 160.90, 151.20, 127.39, 127.16, 115.95, 113.35, 110.54, 110.10, 54.41, 39.07.

**4i:**

2-(4-Fluorophenyl)-5-(4-methoxyphenyl)-1,3,4-oxadiazole(**4i**): White solid, yield 53%; m.p. 178.0-178.9 °C; ^1^H-NMR (400 MHz, CDCl_3_) δ 8.17-8.14(q, 2*H*, Ar-*H*), 8.10-8.08(d, 2*H*, Ar-*H*), 7.27-7.22(q, 2*H*, Ar-*H*), 7.07-7.05(d, 2*H*, Ar-*H*), 3.92(s, 3*H*, -OC*H*_3_); ^13^C-NMR (101 MHz, CDCl_3_) δ 164.49, 163.55, 162.82, 162.31, 161.37, 128.08, 128.02, 127.67, 119.41, 115.43, 115.28, 113.51, 54.46.

**4j:**

2-(4-(Tert-butyl) phenyl)-5-(4-methoxyphenyl)-1,3,4-oxadiazole(**4j**): White solid, yield 47%; m.p. 161.6-162.3 °C; ^1^H-NMR (400 MHz, CDCl_3_) δ 8.12-8.07(q, 4*H*, Ar-*H*), 7.58-7.56(d, 2*H*, Ar-*H*), 7.07-7.05(d, 2*H*, Ar-*H*), 3.92(s, 3*H*, -OC*H*_3_), 1.40 (s, 9*H*, -OC*H*_3_); ^13^C-NMR (101 MHz, CDCl_3_) δ 164.32, 164.23, 162.25, 155.11, 128.65, 126.66, 126.01, 121.26, 116.58, 114.47, 55.47, 35.07, 31.14.

**4k:**

2-(Furan-2-yl)-5-(4-methoxyphenyl)-1,3,4-oxadiazole(**4k**): White solid, yield 66%; m.p. 130.1-131.7 °C; ^1^H-NMR (400 MHz, CDCl_3_) δ 8.10-8.08(d, 2*H*, Ar-*H*), 7.69(s, 1*H*, Ar-*H*), 7.24-7.23(d, 1*H*, Ar-*H*), 7.06-7.04(d, 2*H*, Ar-*H*), 6.65-6.64(q, 1*H*, Ar-*H*), 3.91(s, 3*H*, -OC*H*_3_); ^13^C-NMR (101 MHz, CDCl_3_) δ 163.94, 162.45, 157.05, 145.54, 139.64, 128.80, 116.00, 114.53, 113.73, 112.16, 55.49.

**4l:**

2-(4-Methoxyphenyl)-5-(thiophen-3-yl)-1,3,4-oxadiazole(**4l**): White solid, yield 73%; m.p. 135.3-136.4 °C; ^1^H-NMR (400 MHz, CDCl_3_) δ 8.12-8.07(q, 3*H*, Ar-*H*), 7.76-7.74(d, 1*H*, Ar-*H*), 7.51-7.49(q, 1*H*, Ar-*H*), 7.06-7.04(d, 2*H*, Ar-*H*), 3.92 (s, 3*H*, -OC*H*_3_); ^13^C-NMR (101 MHz, CDCl_3_) δ 163.92, 162.30, 160.91, 128.67, 127.33, 127.09, 126.04, 125.46, 116.36, 114.49, 55.47.

**2. Synthesis and spectral data of compounds 5a–5l.**

**5a:**

2-(4-Methoxyphenyl)-5-phenyl-1,3,4-oxadiazole (**5a**): White solid, yield 71%; m.p. 149.3-150.9 °C; ^1^H-NMR (400 MHz, CDCl_3_) δ 8.17-8.14(m, 2*H*, Ar-*H*), 8.12-8.10(m, 2*H*, Ar-*H*), 7.57-7.55(m, 3*H*, Ar-*H*), 7.07-7.05(d, 2*H*, Ar-*H*), 3.92(s, 3*H*, -OC*H*_3_); ^13^C-NMR (101 MHz, CDCl_3_) δ 164.54, 164.13, 162.34, 131.54, 129.04, 128.70, 126.82, 124.07, 116.42, 114.51, 55.49.

**5b:**

2-(3,4-Dimethoxyphenyl)-5-phenyl-1,3,4-oxadiazole (**5b**): White solid, yield 85%; m.p. 132.3-133.2 °C; ^1^H-NMR (400 MHz, CDCl_3_) δ 8.18-8.14(m, 2*H*, Ar-*H*), 7.74-7.68(m, 2*H*, Ar-*H*), 7.57-7.55(m, 3*H*, Ar-*H*), 7.02-7.00(d, 1*H*, Ar-*H*), 4.03-3.99(d, 6*H*, -OC*H*_3_); ^13^C-NMR (101 MHz, CDCl_3_) δ 164.56, 164.25, 152.03, 149.36, 131.59, 129.05, 126.86, 124.03, 120.41, 116.52, 111.08, 109.45, 56.19, 56.07.

**5c:**

2-Phenyl-5-(3,4,5-trimethoxyphenyl)-1,3,4-oxadiazole (**5c**): White solid, yield 90%; m.p. 126.0-127.2 °C; ^1^H-NMR (400 MHz, CDCl_3_) δ 8.19-8.16(m, 2*H*, Ar-*H*), 7.60-7.55(m, 3*H*, Ar-*H*), 7.40-7.39(d, 2*H*, Ar-*H*), 4.02-3.93(m, 9*H*, -OC*H*_3_); ^13^C-NMR (101 MHz, CDCl_3_) δ 164.52, 153.70, 131.75, 129.08, 126.94, 123.89, 119.02, 104.20, 61.06, 56.43.

**5d:**

2-Phenyl-5-(2,3,4-trimethoxyphenyl)-1,3,4-oxadiazole (**5d**): White solid, yield 63%; m.p. 121.6-122.5 °C; ^1^H-NMR (400 MHz, CDCl_3_) δ 8.17-8.15(m, 2*H*, Ar-*H*), 7.81-7.79(d, 1*H*, Ar-*H*), 7.57-7.55(m, 3*H*, Ar-*H*), 6.86-6.84(d, 1*H*, Ar-*H*) , 4.08 (s, 3*H*, -OC*H*_3_) , 3.98-3.96 (d, 6*H*, -OC*H*_3_); ^13^C-NMR (101 MHz, CDCl_3_) δ 164.30, 163.04, 156.68, 152.87, 143.11, 131.51, 129.05, 126.84, 125.02, 124.20, 111.45, 107.88, 61.70, 61.09, 56.19.

**5e:**

2-Phenyl-5-(2,4,5-trimethoxyphenyl)-1,3,4-oxadiazole (**5e)**: White solid, yield 61%; m.p. 113.8-114.3 °C; ^1^H-NMR (400 MHz, DMSO) δ 8.08(s, 2*H*, Ar-*H*), 7.64-7.63 (t, 3*H*, Ar-*H*), 7.49-7.48 (d, 1*H*, Ar-*H*), 6.91(s, 1*H*, Ar-*H*), 3.96-3.92(d, 6*H*, -OC*H*_3_), 3.82(s ,3*H*, -OC*H*_3_); ^13^C-NMR (101 MHz, DMSO) δ 163.77, 163.39, 153.52, 143.29, 132.22, 129.89, 129.02, 126.90, 124.04, 112.68, 103.31, 99.04, 57.31, 56.63, 56.40.

**5f:**

2-(4-Fluorophenyl)-5-phenyl-1,3,4-oxadiazole (**5f**): White solid, yield 60%; m.p. 155.1-155.6 °C; ^1^H-NMR (400 MHz, CDCl_3_) δ 8.20-8.15(m, 4*H*, Ar-*H*), 7.61-7.54(m, 3*H*, Ar-*H*), 7.28-7.24(t, 2*H*, Ar-*H*); ^13^C-NMR (151 MHz, CDCl_3_) δ 165.64, 164.62, 163.96, 163.79, 131.81, 129.24, 129.18, 129.11, 126.92, 123.81, 120.29, 116.52, 116.37.

**5g:**

2-(4-Chlorophenyl)-5-phenyl-1,3,4-oxadiazole (**5g**): White solid, yield 71%; m.p. 163.1-163.8 °C; ^1^H-NMR (400 MHz, CDCl_3_) δ 8.17-8.10(m, 4*H*, Ar-*H*), 7.58-7.54(m, 5*H*, Ar-*H*); ^13^C-NMR (151 MHz, CDCl_3_) δ 164.74, 163.79, 138.01, 131.89, 129.49, 129.13, 128.18, 126.96, 123.71, 122.39.

**5h:**

2-([1,1'-Biphenyl]-4-yl)-5-phenyl-1,3,4-oxadiazole (**5h**): Pale yellow solid, yield 67%; m.p. 166.9-167.2 °C; ^1^H-NMR (400 MHz, CDCl_3_) δ 8.26-8.19(m, 4*H*, Ar-*H*), 7.81-7.79(d, 2*H*, Ar-*H*), 7.70-7.68(d, 2*H*, Ar-*H*), 7.59-7.44(m, 6*H*, Ar-*H*); ^13^C-NMR (151 MHz, CDCl_3_) δ 164.57, 144.45, 139.79, 131.72, 129.08, 128.99, 128.18, 127.70, 127.38, 127.16, 126.94, 123.94, 122.66.

**5i:**

2-(Furan-2-yl)-5-phenyl-1,3,4-oxadiazole (**5i**): Pale yellow solid, yield 67%; m.p. 103.7-104.1 °C; ^1^H-NMR (400 MHz, CDCl_3_) δ 8.17-8.14(q, 2*H*, Ar-*H*), 7.70(d, 1*H*, furan-*H*), 7.59-7.55(m, 3*H*, Ar-*H*), 7.29-7.26(t, 1*H*, furan-*H*), 6.66-6.65(q, 1*H*, furan-*H*); ^13^C-NMR (151 MHz, CDCl_3_) δ 163.96, 157.47, 145.74, 139.49, 131.87, 129.10, 127.01, 123.52, 114.11, 112.22.

**5j:**

2-(4-(Tert-butyl) phenyl)-5-phenyl-1,3,4-oxadiazole (**5j**): White solid, yield 60%; m.p. 93.5-94.1 °C; ^1^H-NMR (400 MHz, DMSO) δ 8.14-8.12(q, 2*H*, Ar-*H*), 8.06-8.04(d, 2*H*, Ar-*H*), 7.65-7.63(d, 5*H*, Ar-*H*), 1.33(s, 9*H*, -C*H*_3_); ^13^C-NMR (101 MHz, DMSO) δ 164.49, 164.27, 155.45, 132.46, 129.89, 127.09, 127.01, 126.70, 123.86, 121.09, 35.31, 31.28.

**5k:**

2-Phenyl-5-(thiophen-3-yl)-1,3,4-oxadiazole (**5k**): White solid, yield 70%; m.p. 112.5-113.0 °C; ^1^H-NMR (400 MHz, DMSO) δ 8.51-8.50(t, 1*H*, thiophen-*H*), 8.13-8.10(m, 2*H*, thiophen-*H*), 7.87-7.85(m, 1*H*, Ar-*H*), 7.74-7.72(q, 1*H*, Ar-*H*), 7.65-7.63(d, 3*H*, Ar-*H*); ^13^C-NMR (101 MHz, DMSO) δ 163.78, 161.40, 132.46, 129.88, 129.53, 129.44, 127.08, 126.15, 124.92, 123.78.

**5l:**

*N, N*-Dimethyl-4-(5-phenyl-1,3,4-oxadiazol-2-yl) aniline (**5l**): Yellow solid, yield 60%; m.p. 120.5-121.1 °C; ^1^H-NMR (400 MHz, CDCl_3_) δ 8.15-8.13(m, 2*H*, Ar-*H*), 8.01-7.99(d, 2*H*, Ar-*H*), 7.55-7.53(t, 3*H*, Ar-*H*), 6.79-6.77(d, 2*H*, Ar-*H*) 3.08(s, 6*H*, -C*H*_3_); ^13^C-NMR (101 MHz, CDCl_3_) δ 165.30, 163.51, 152.31, 131.23, 128.98, 128.33, 126.70, 124.37, 111.58, 110.87, 40.14.

**3. Synthesis and spectral data of compounds 6a–6f.**

**6a:**

2-(4-Chlorophenyl)-5-(*p*-tolyl)-1,3,4-oxadiazole(**6a**): White solid, yield 80%; m.p. 206.2-207.5 °C; ^1^H-NMR (400 MHz, CDCl_3_) δ 8.11-8.03(q, 4*H*, Ar-*H*), 7.55-7.52(d, 2*H*, Ar-*H*), 7.37-7.35(d, 2*H*, Ar-*H*), 2.47 (s, 3*H*, -C*H*_3_); ^13^C-NMR (101 MHz, CDCl_3_) δ 164.89, 163.54, 142.51, 137.88, 129.84, 129.46, 128.15, 126.92, 122.48, 120.92, 21.72.

**6b:**

2-(*p*-Tolyl)-5-(2,3,4-trimethoxyphenyl)-1,3,4-oxadiazole(**6b**): White powdery solid, yield 79%; m.p. 164.5-164.8 °C; ^1^H-NMR (400 MHz, CDCl_3_) δ 8.05-8.03(d, 2*H*, Ar-*H*), 7.80-7.78(d, 1*H*, Ar-*H*), 7.36-7.34(d, 2*H*, Ar-*H*), 6.85-6.83(d, 2*H*, Ar-*H*), 4.07(s, 3*H*, -OC*H*_3_), 3.97-3.96(d, 6*H*, -OC*H*_3_), 2.46(s, 3*H*, -C*H*_3_); ^13^C-NMR (101 MHz, CDCl_3_) δ 164.45, 162.75, 156.59, 152.81, 143.06, 142.06, 129.76, 126.81, 125.00, 121.35, 111.50, 107.83, 61.72, 61.12, 56.20, 21.70.

**6c:**

2-(*p*-Tolyl)-5-(2,4,5-trimethoxyphenyl)-1,3,4-oxadiazole(**6c**): White solid, yield 70%; m.p. 114.5-115.3 °C; ^1^H-NMR (400 MHz, DMSO) δ 7.96-7.94(d, 2*H*, Ar-*H*), 7.45-7.41(t, 3*H*, Ar-*H*), 6.88(s, 1*H*, Ar-*H*), 3.95-3.91(d, 6*H*, -OC*H*_3_), 3.81(s, 3*H*, -OC*H*_3_), 2.40(s, 3*H*, -C*H*_3_); ^13^C-NMR (101 MHz, DMSO) δ 163.85, 163.12, 153.45, 143.27, 142.29, 130.39, 126.84, 121.30, 112.67, 103.39, 99.02, 57.29, 56.62, 56.37, 21.60.

**6d:**

2-(p-tolyl)-5-(3,4,5-trimethoxyphenyl)-1,3,4-oxadiazole(**6d**): White solid, yield 65%; m.p. 124.3-125.0 °C; ^1^H-NMR (400 MHz, DMSO) δ 8.06-8.04(d, 2*H*, Ar-*H*), 7.44-7.42(d, 2*H*, Ar-*H*), 7.38(s, 2*H*, Ar-*H*), 3.92(s, 6*H*, -OC*H*_3_), 3.76(s, 3*H*, -OC*H*_3_), 2,41(s, 3*H*, -C*H*_3_); ^13^C-NMR (101 MHz, DMSO) δ 164.49, 164.15, 153.91, 142.65, 140.96, 130.36, 127.13, 121.00, 119.01, 104.44, 60.69, 56.65, 21.63.

**6e:**

2-(3,4-dimethoxyphenyl)-5-(p-tolyl)-1,3,4-oxadiazole(**6e**): White powdery solid, yield 71%; m.p. 129.2-129.9 °C; ^1^H-NMR (400 MHz, CDCl_3_) δ 8.04-8.02(d, 2*H*, Ar-*H*), 7.72-7.67(m, 2*H*, Ar-*H*), 7.35-7.34(d, 2*H*, Ar-*H*), 7.01-6.99(d, 1*H*, Ar-*H*), 4.02-3.98(d, 6*H*, -OC*H*_3_), 2.45(s, 3*H*, -C*H*_3_); ^13^C-NMR (101 MHz, CDCl_3_) δ 164.40, 164.31, 151.91, 149.30, 142.15, 129.75, 126.81, 121.19, 120.35, 116.56, 111.01, 109.36, 56.19, 56.07, 21.70.

**6f:**

2-([1,1'-biphenyl]-4-yl)-5-(p-tolyl)-1,3,4-oxadiazole(**6f**): White powdery solid, yield 80%; m.p. 165.0-165.2 °C; ^1^H-NMR (400 MHz, CDCl_3_) δ 8.24-8.22(d, 2*H*, Ar-*H*), 8.08-8.06(d, 2*H*, Ar-*H*), 7.79-7.77(d, 2*H*, Ar-*H*), 7.69-7.67(d, 2*H*, Ar-*H*), 7.53-7.36(m, 5*H*, Ar-*H*), 2.48(s, 3*H*, -C*H*_3_); ^13^C-NMR (101 MHz, CDCl_3_) δ 164.75, 164.25, 144.35, 142.33, 139.83, 129.82, 129.01, 128.19, 127.70, 127.36, 127.18, 126.92, 122.74, 121.13, 21.73.

**4. Copies of NMR spectra of 1, 3, 4-Oxadiazole Derivatives.**


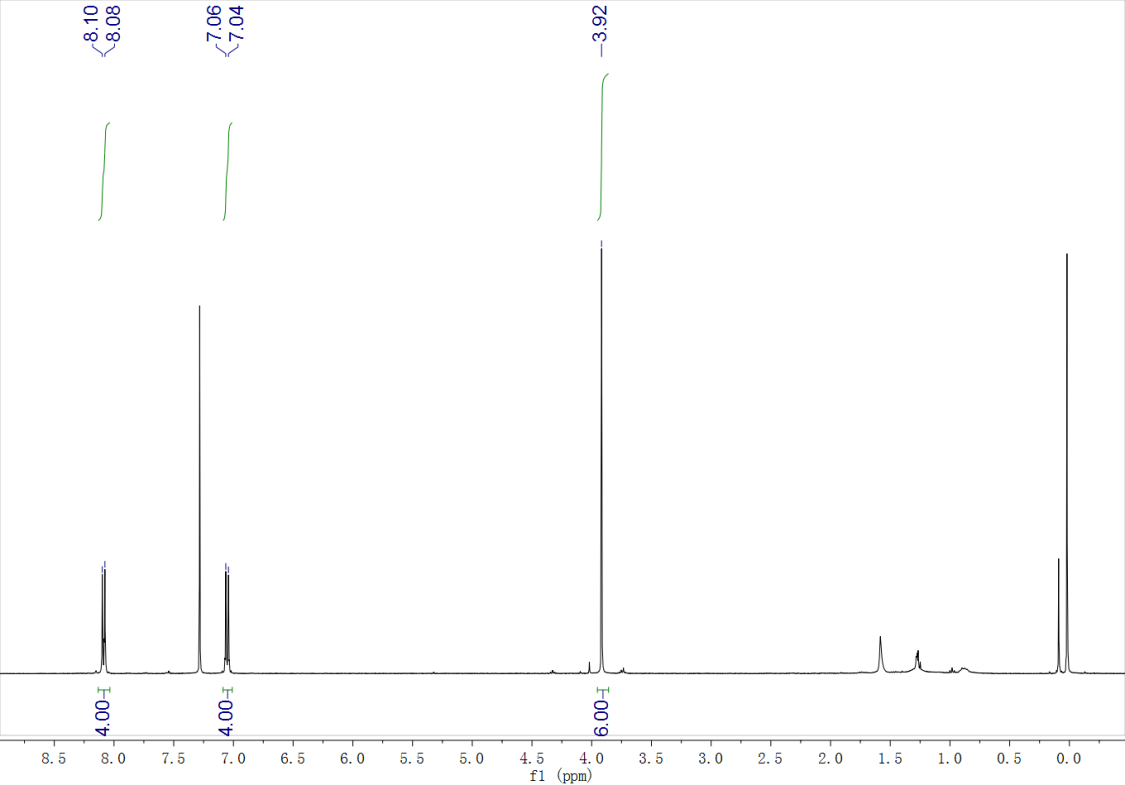


**Figure S1.** ^1^H-NMR spectrum of compound **4a**


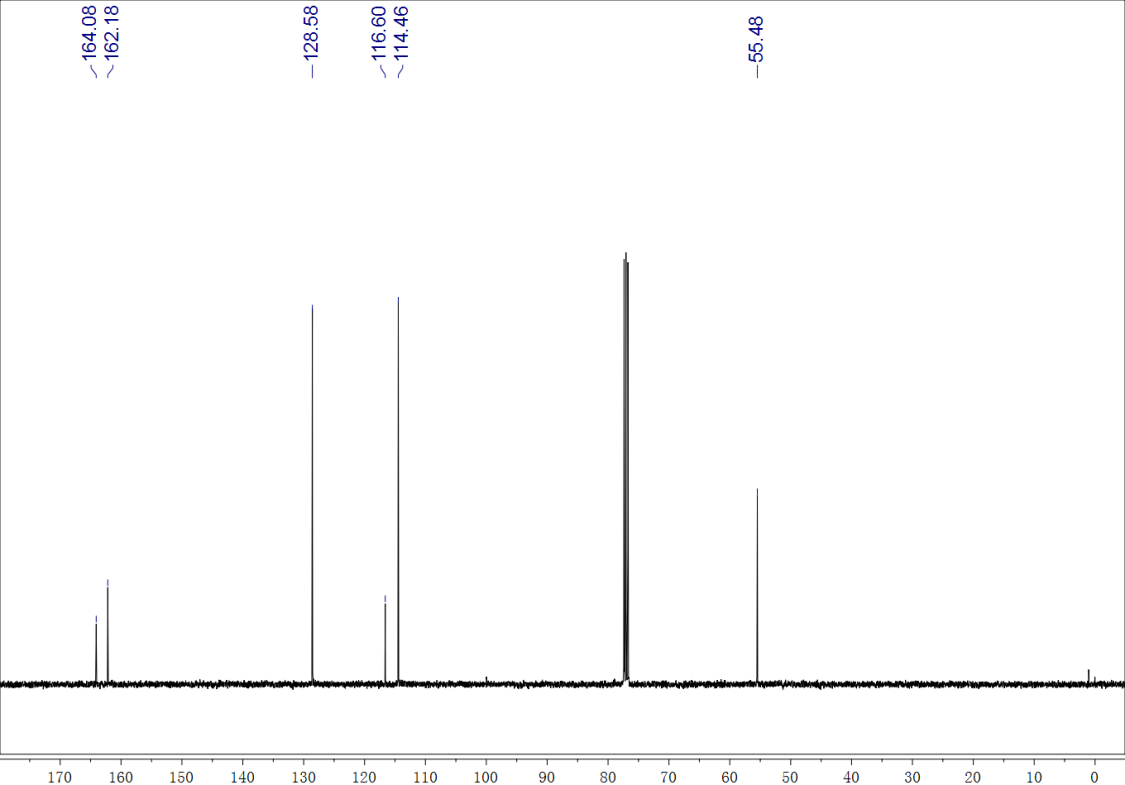


**Figure S2.** ^13^C-NMR spectrum of compound **4a**


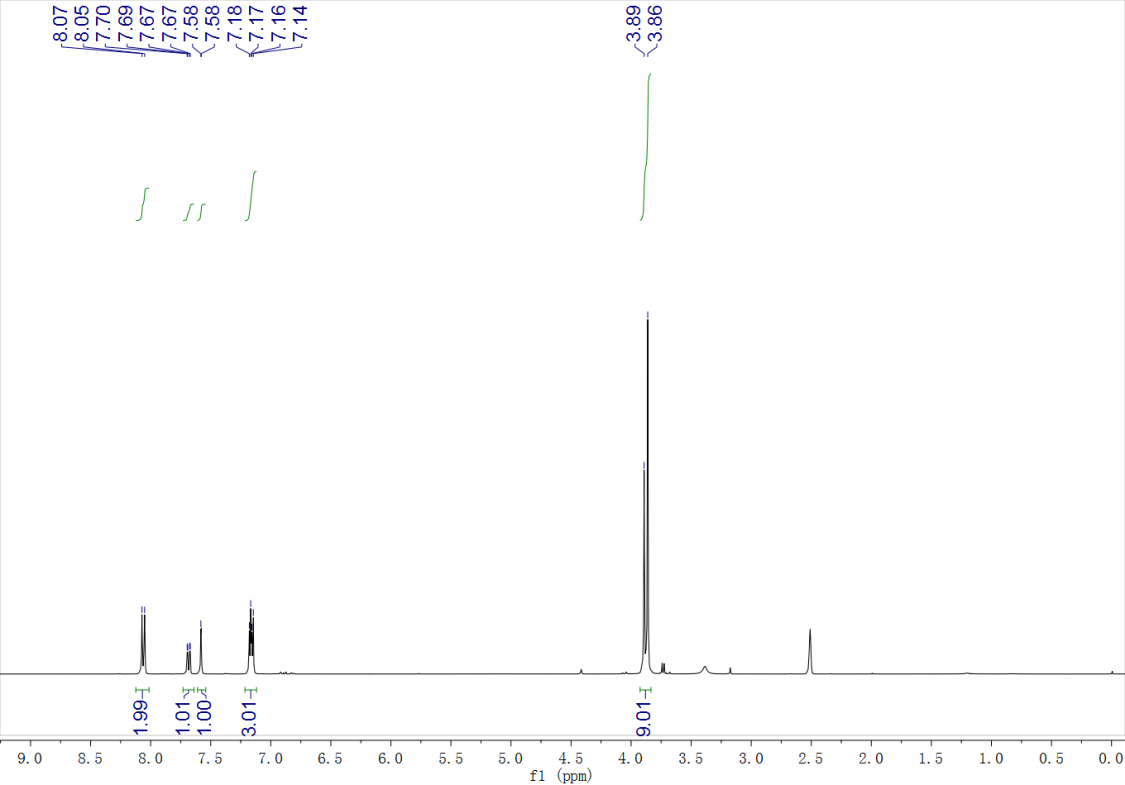


**Figure S3.** ^1^H-NMR spectrum of compound **4b**


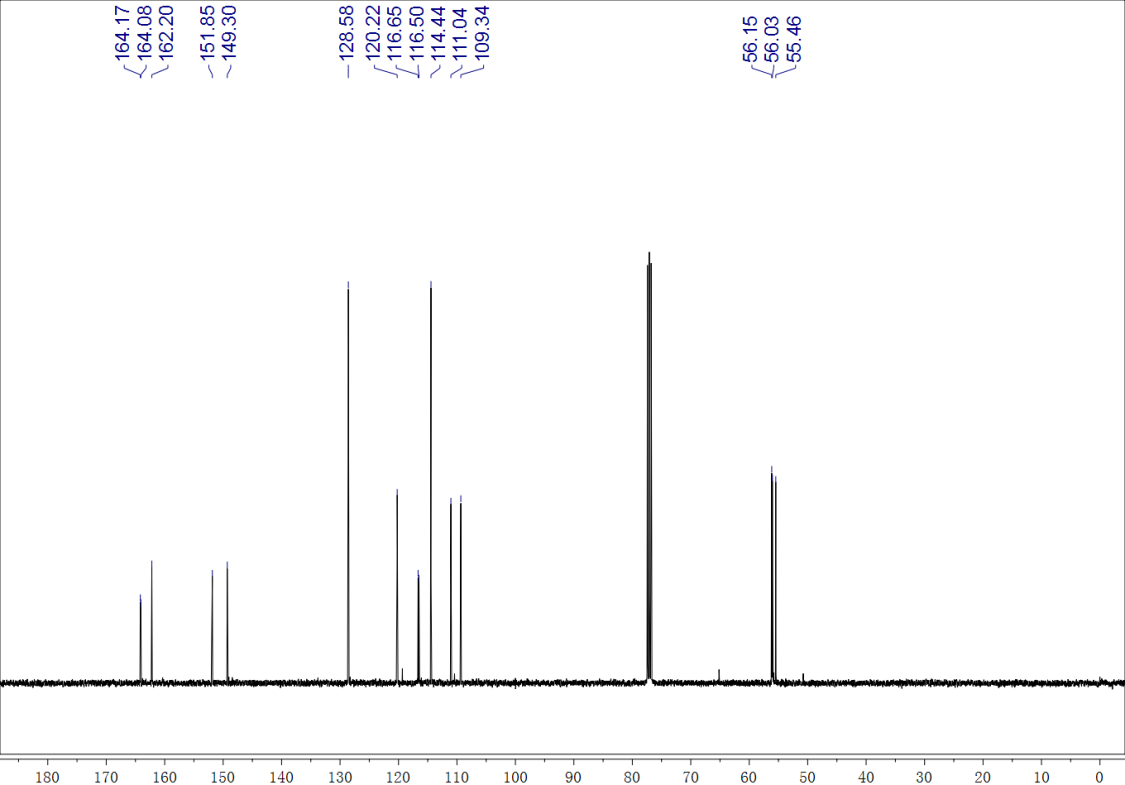


**Figure S4.** ^13^C-NMR spectrum of compound **4b**


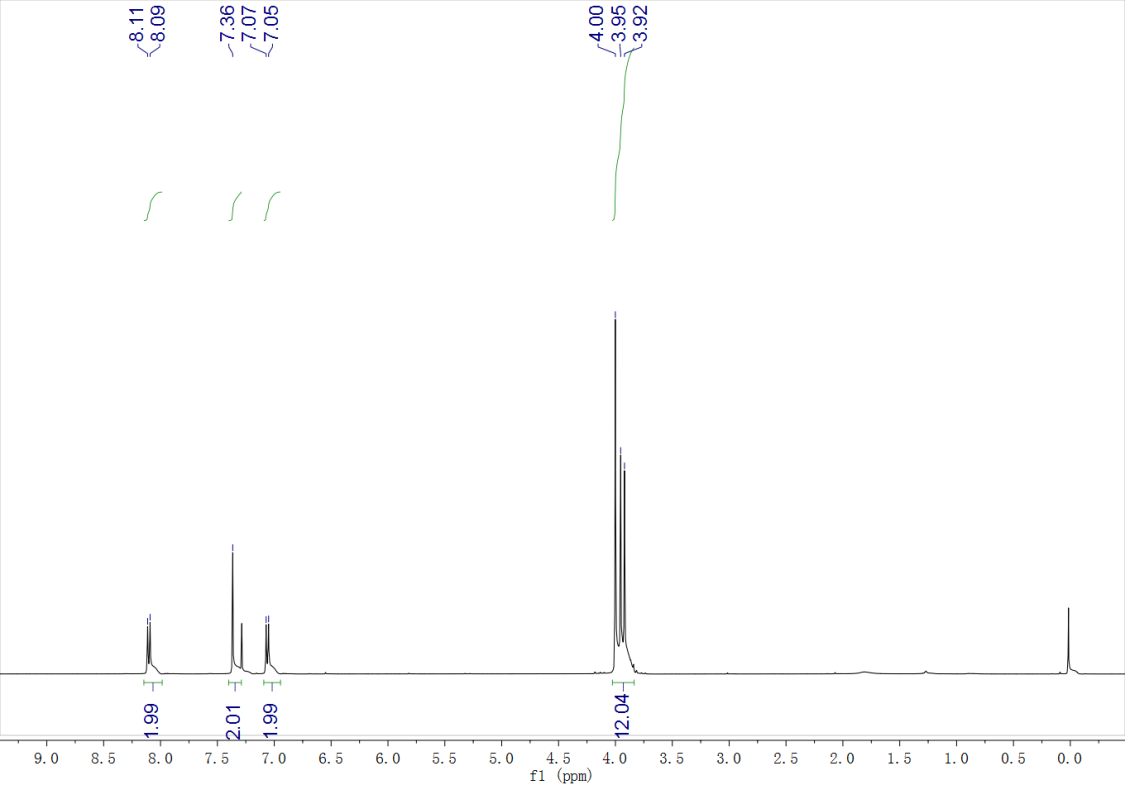


**Figure S5.** ^1^H-NMR spectrum of compound **4c**


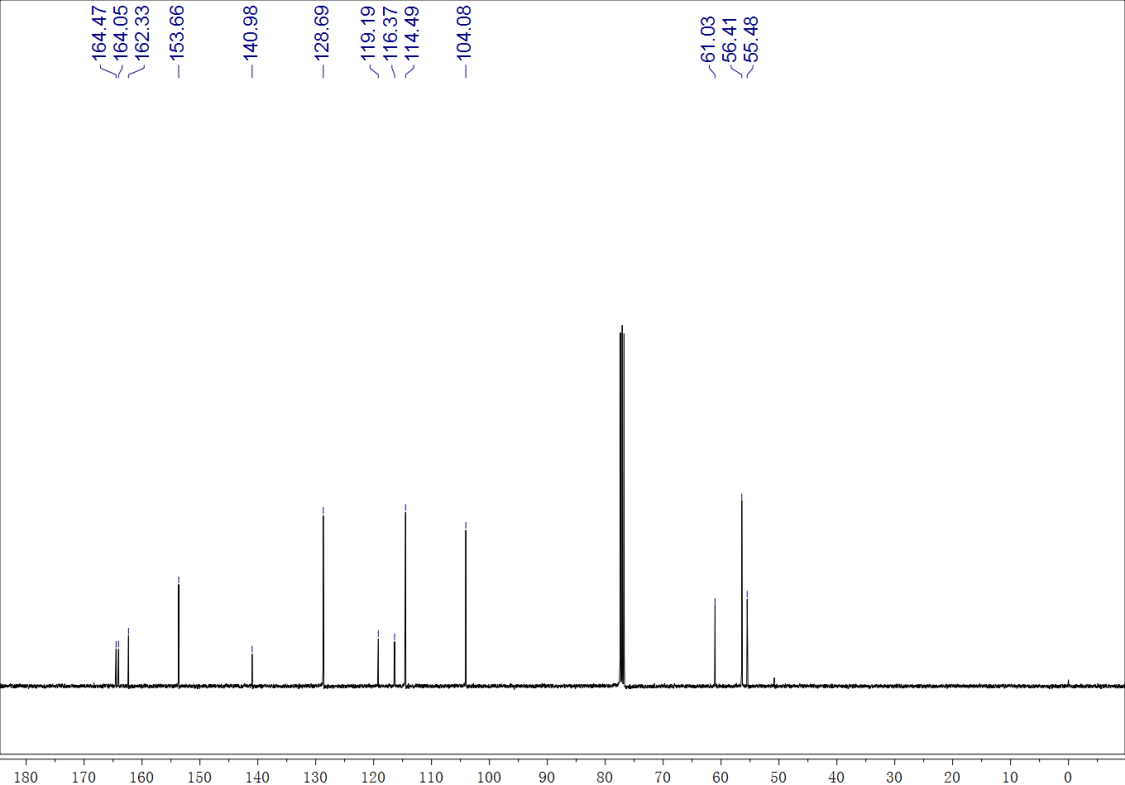


**Figure S6.** ^13^C-NMR spectrum of compound **4c**


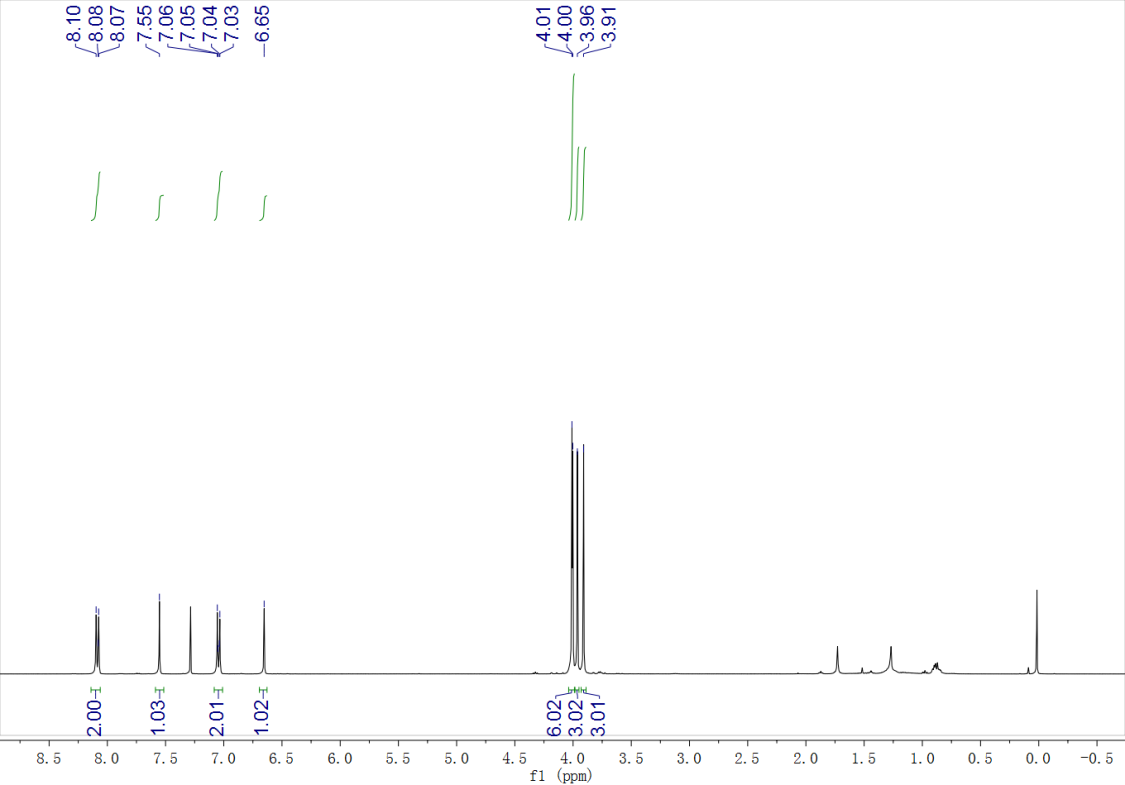


**Figure S7.** ^1^H-NMR spectrum of compound **4d**


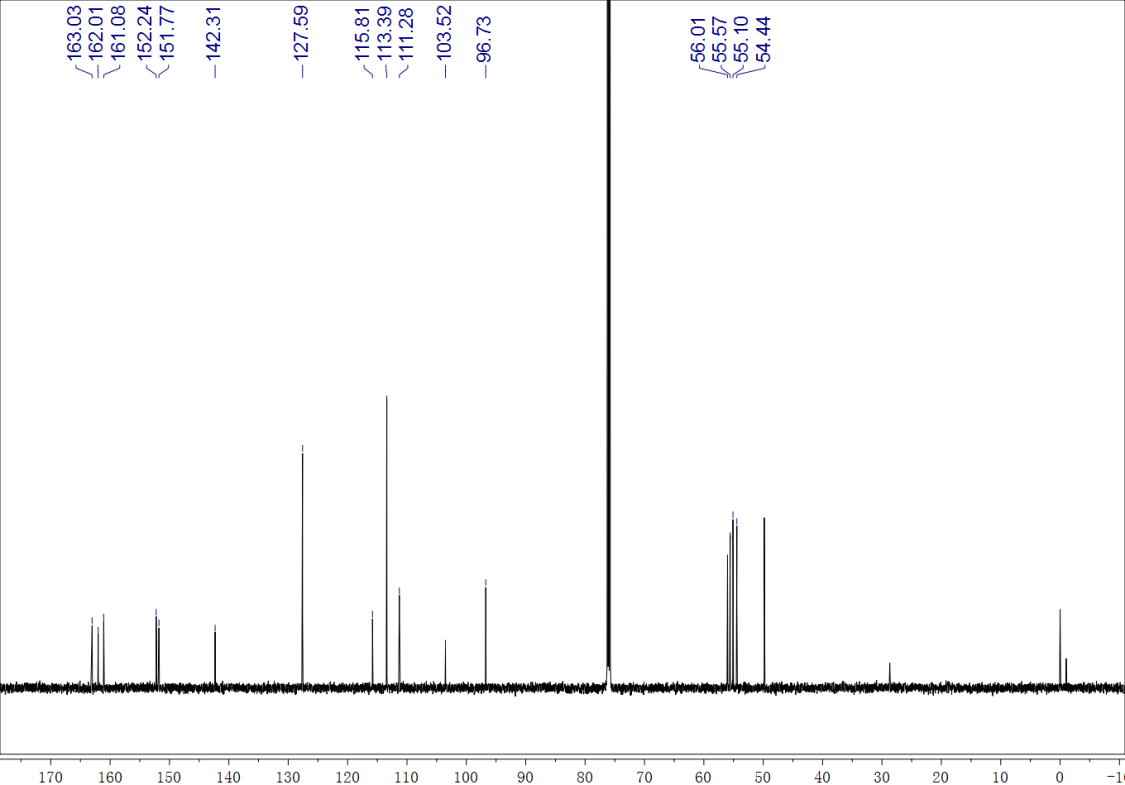


**Figure S8.** ^13^C-NMR spectrum of compound **4d**


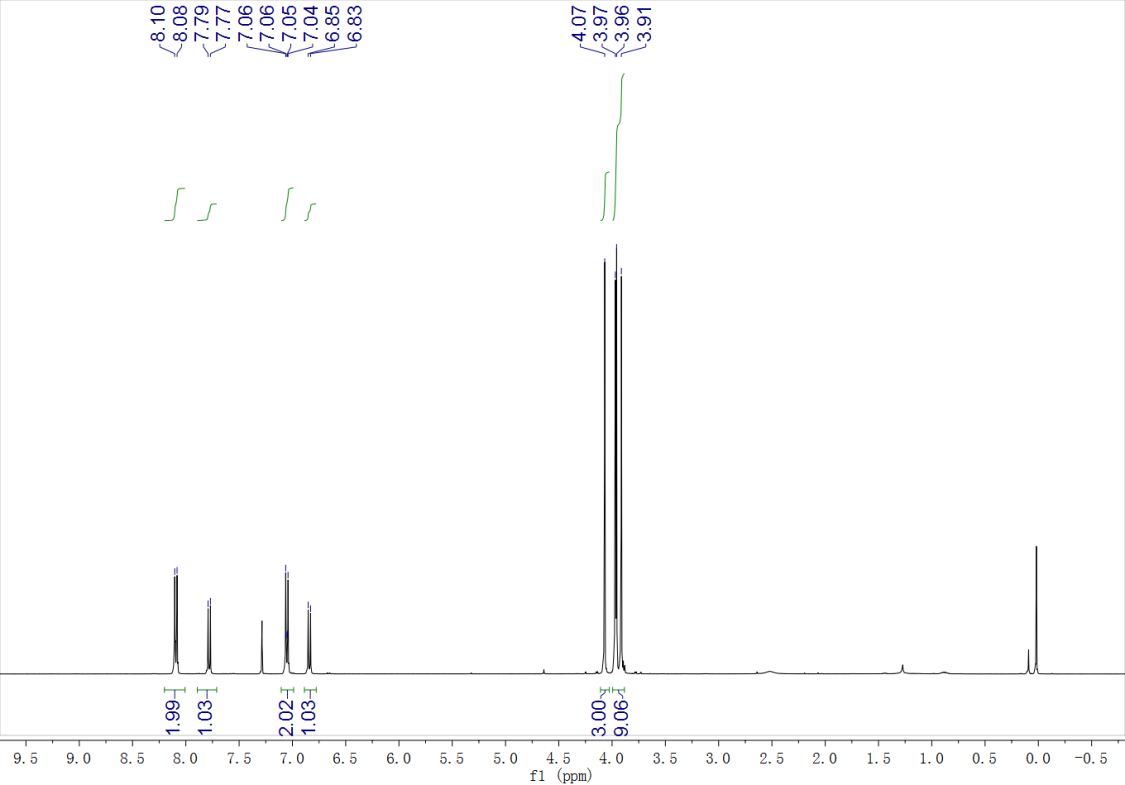


**Figure S9.** ^1^H-NMR spectrum of compound **4e**


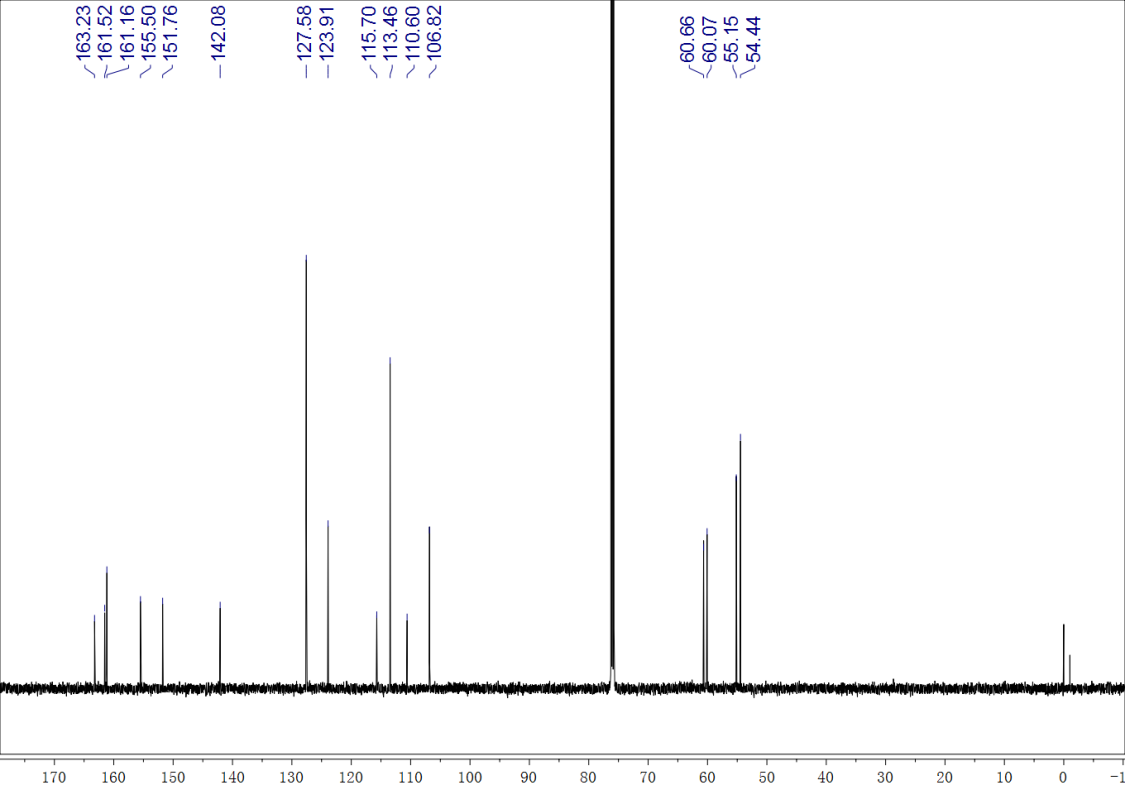


**Figure S10.** ^13^C-NMR spectrum of compound **4e**


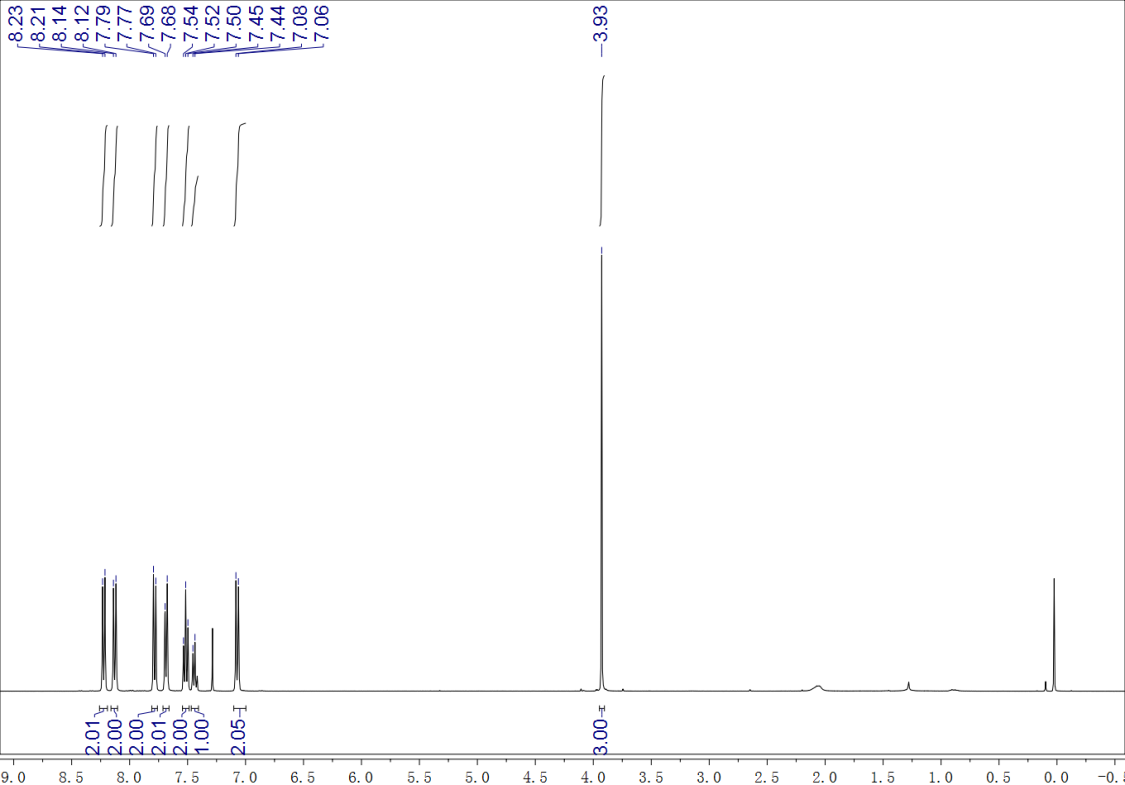


**Figure S11.** ^1^H-NMR spectrum of compound **4f**


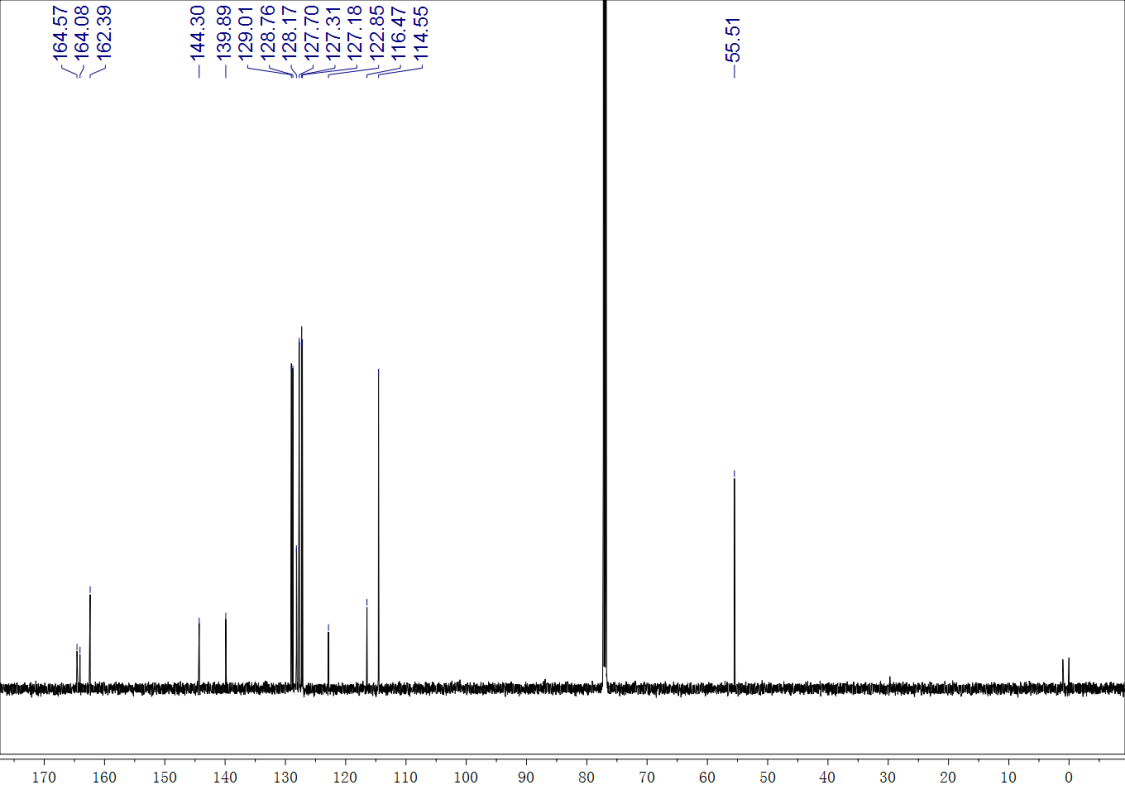


**Figure S12.** ^13^C-NMR spectrum of compound **4e**


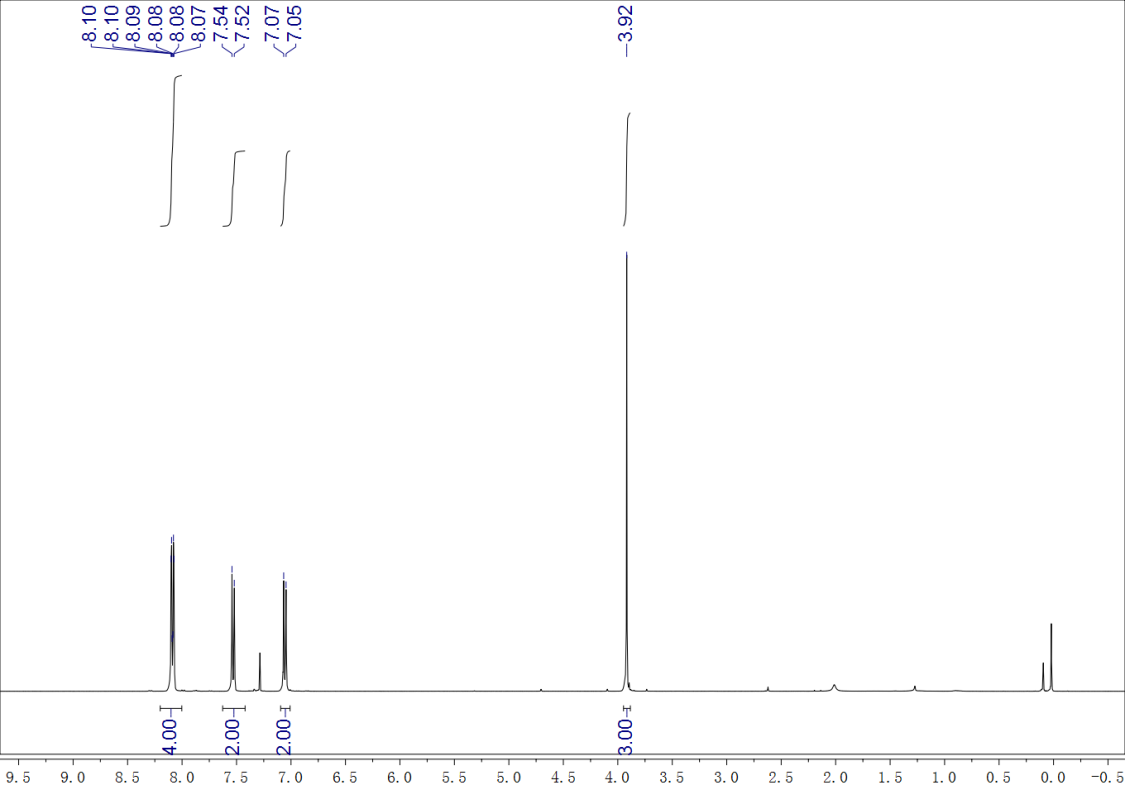


**Figure S13.** ^1^H-NMR spectrum of compound **4g**


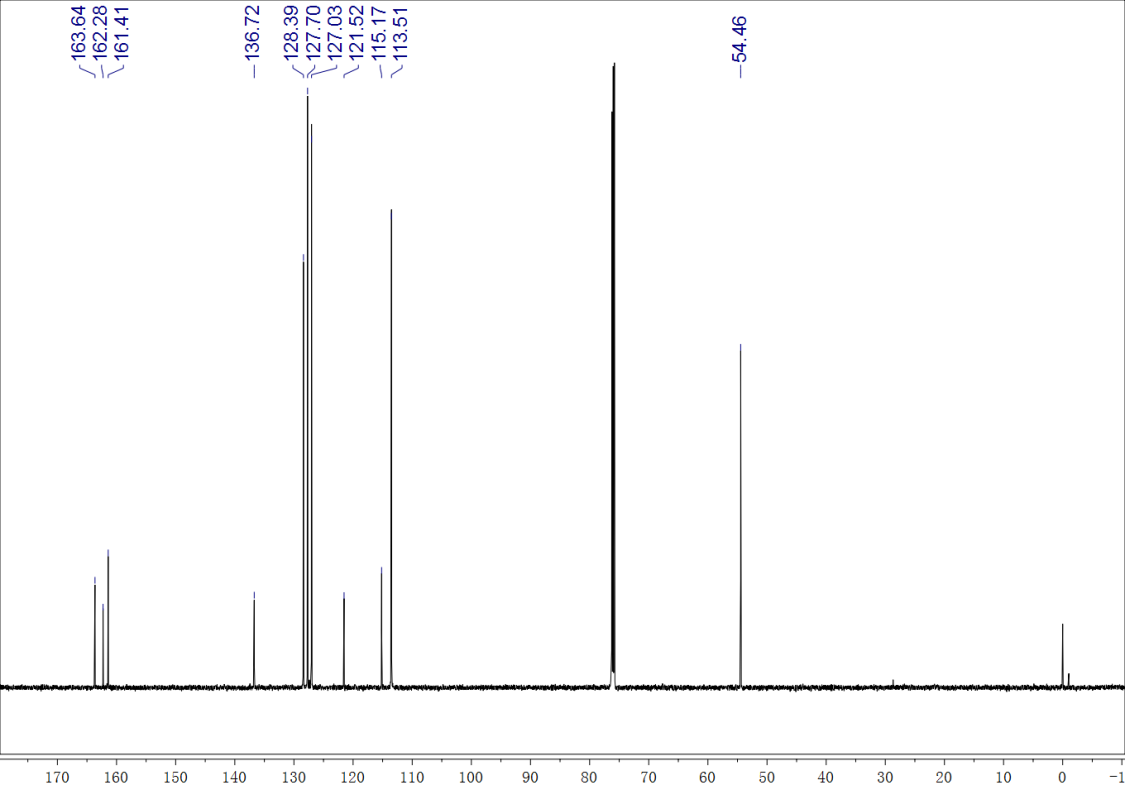


**Figure S14.** ^13^C-NMR spectrum of compound **4g**

**
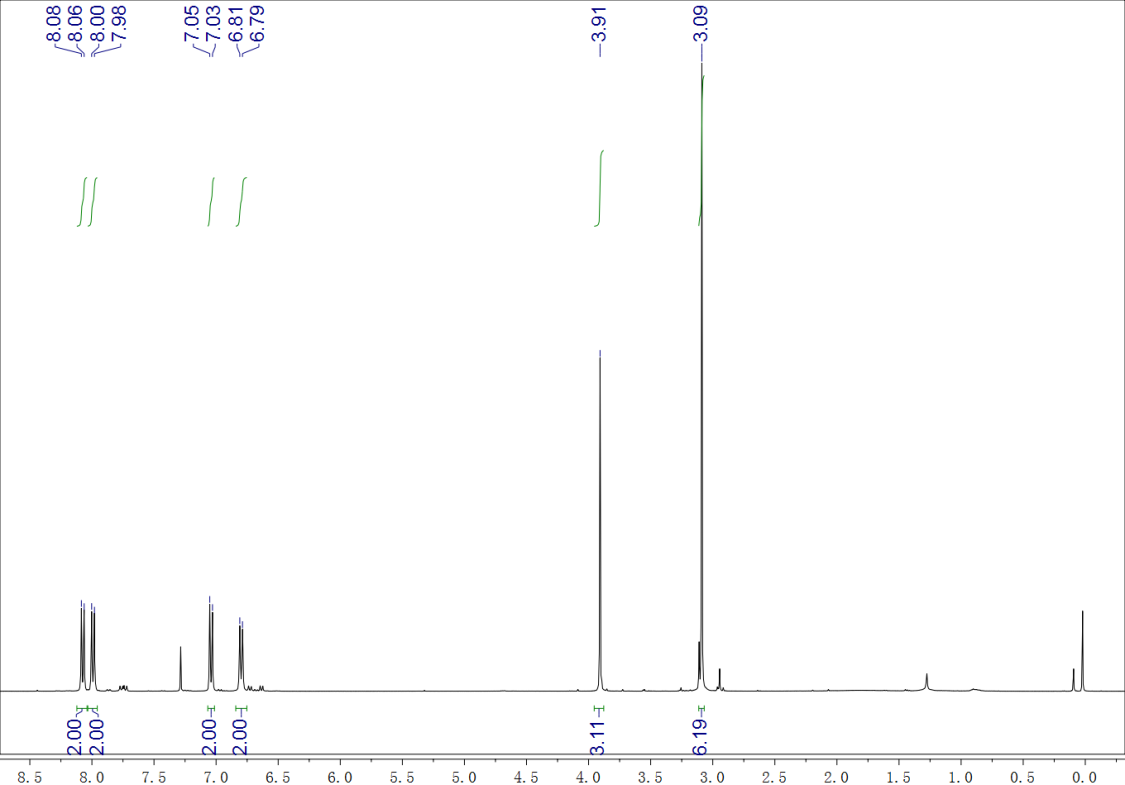
**

**Figure S15.** ^1^H-NMR spectrum of compound **4h**

**
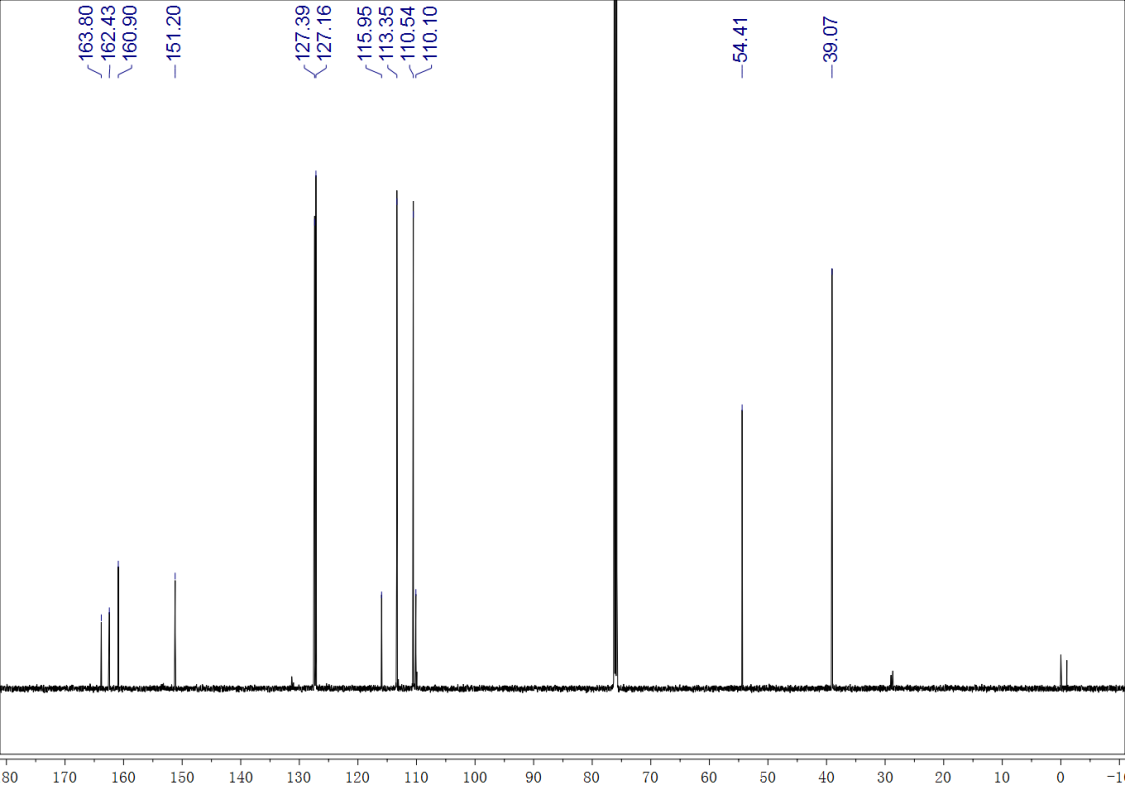
**

**Figure S16.** ^13^C-NMR spectrum of compound **4h**


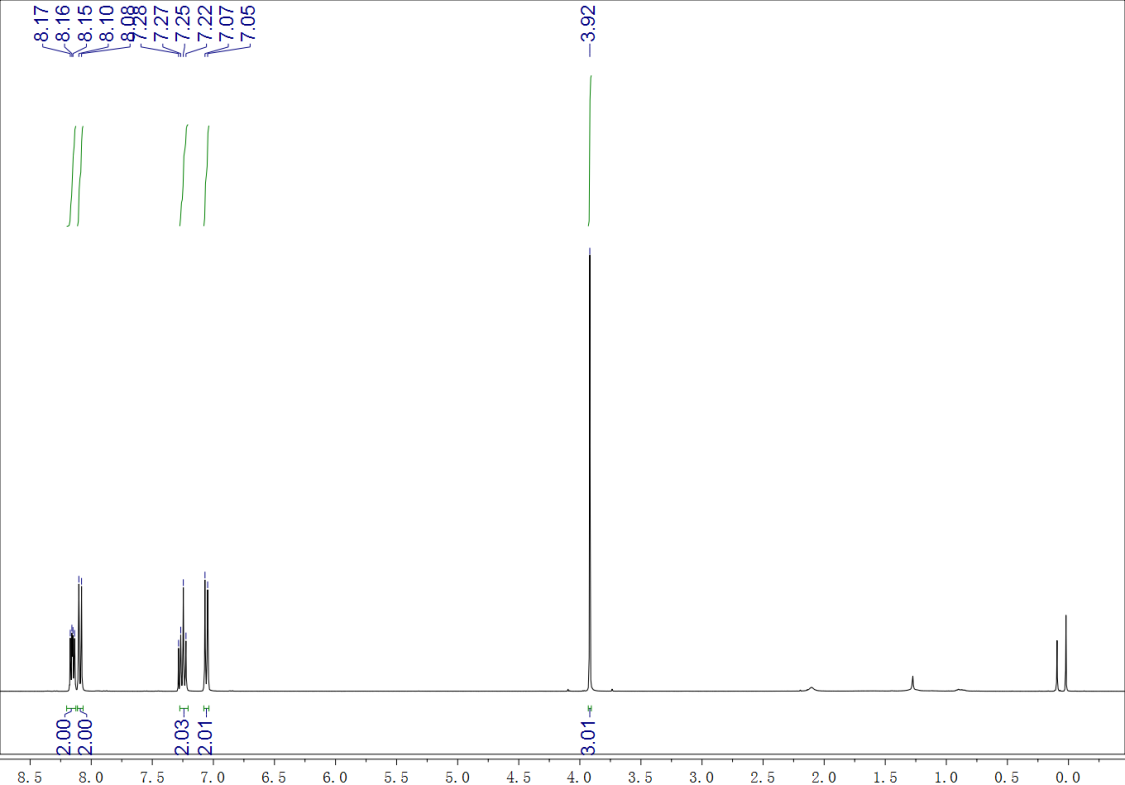


**Figure S17.** ^1^H-NMR spectrum of compound **4i**


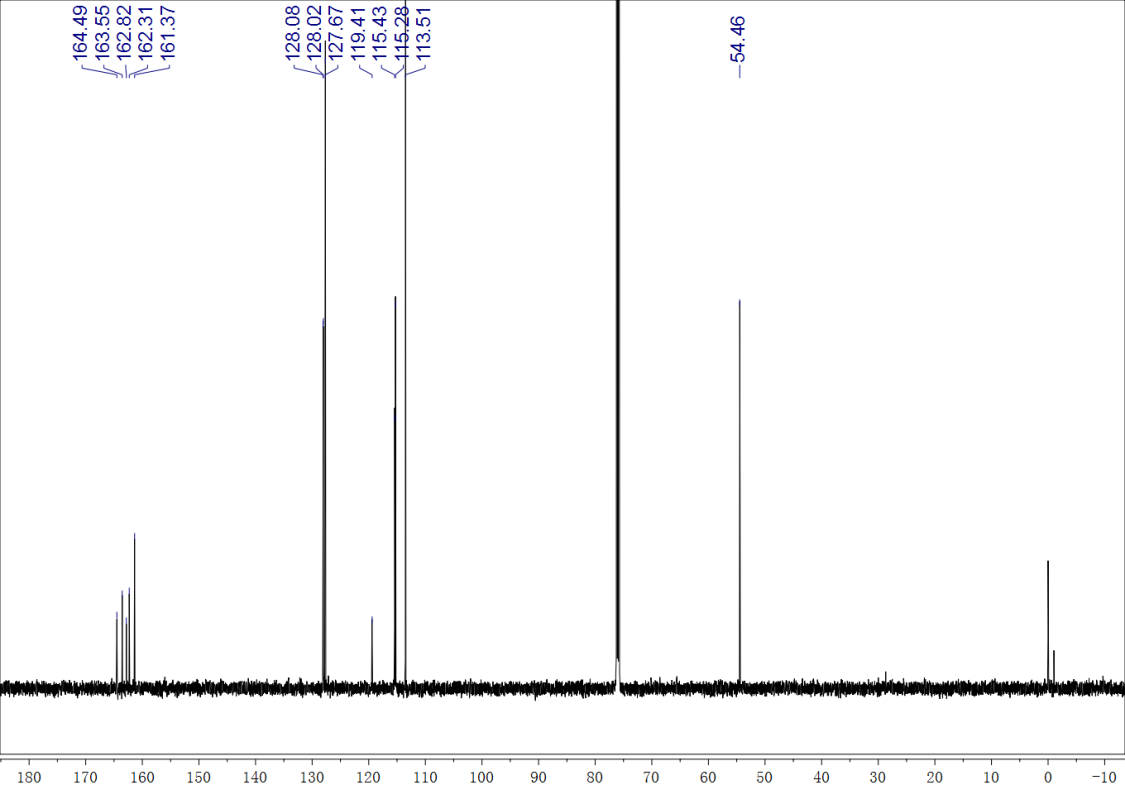


**Figure S18.** ^13^C-NMR spectrum of compound **4i**

**
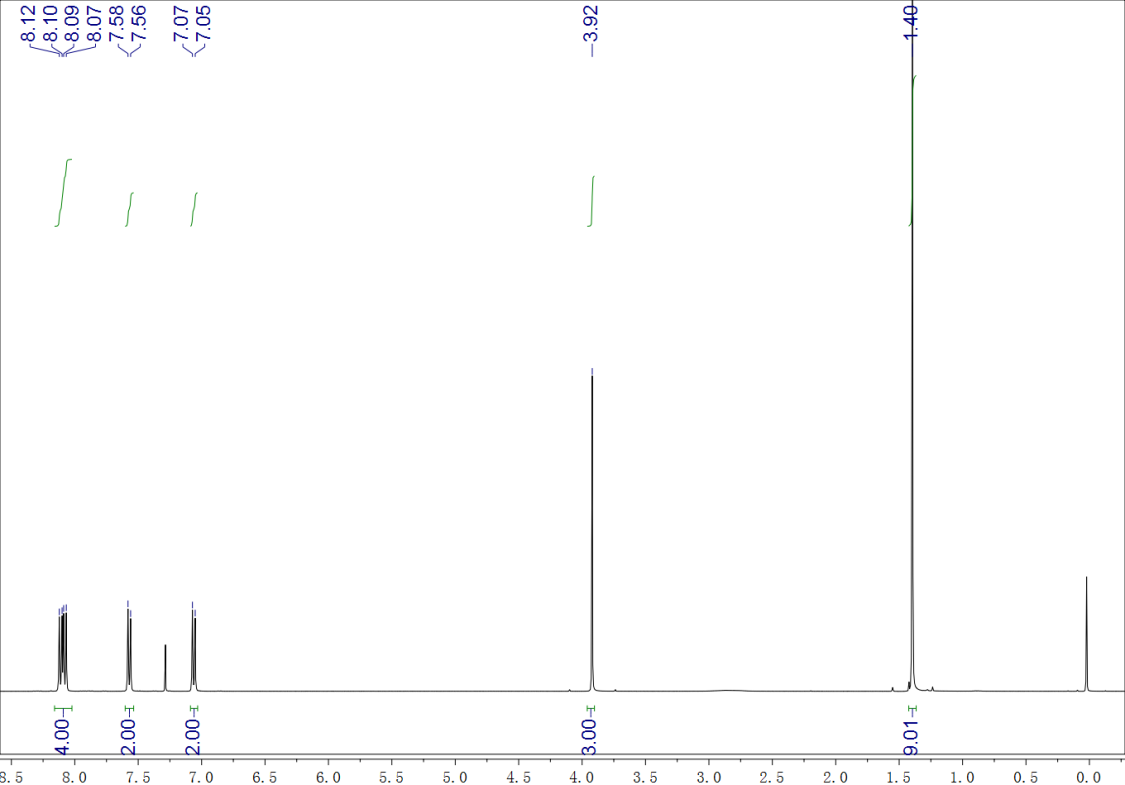
**

**Figure S19.** ^1^H-NMR spectrum of compound **4j**

**
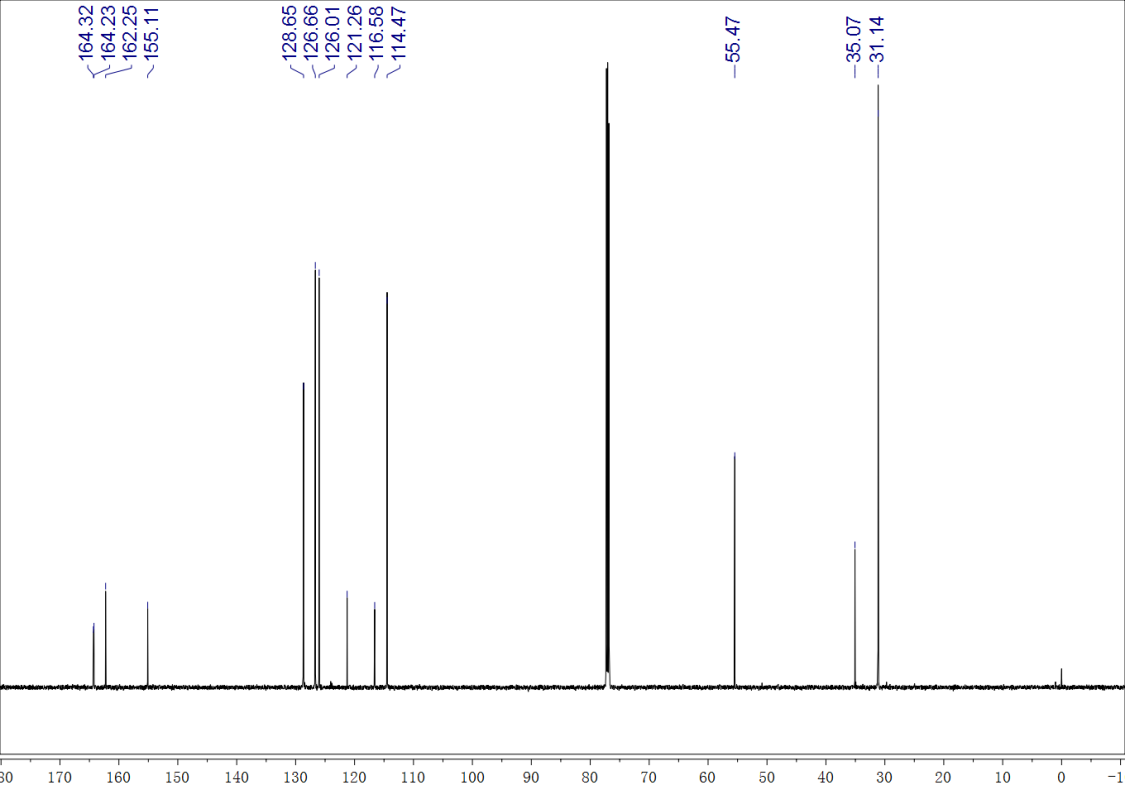
**

**Figure S20.** ^13^C-NMR spectrum of compound **4j**


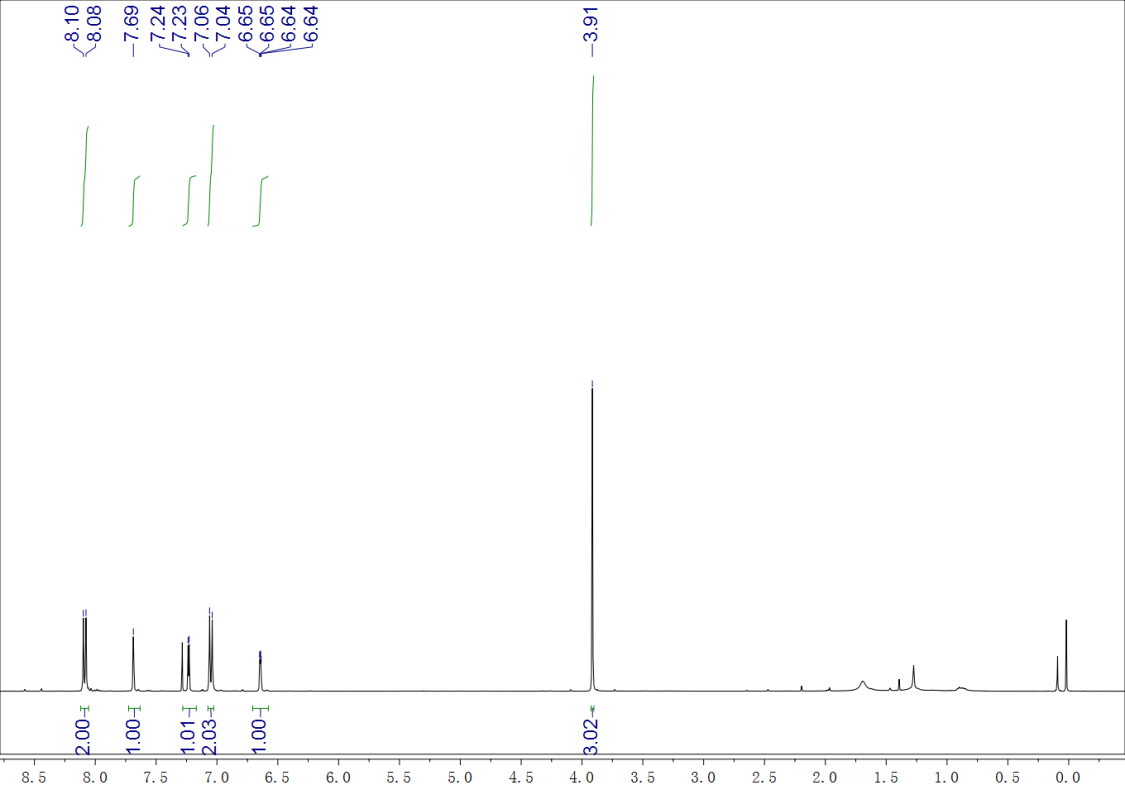


**Figure S21.** ^1^H-NMR spectrum of compound **4k**


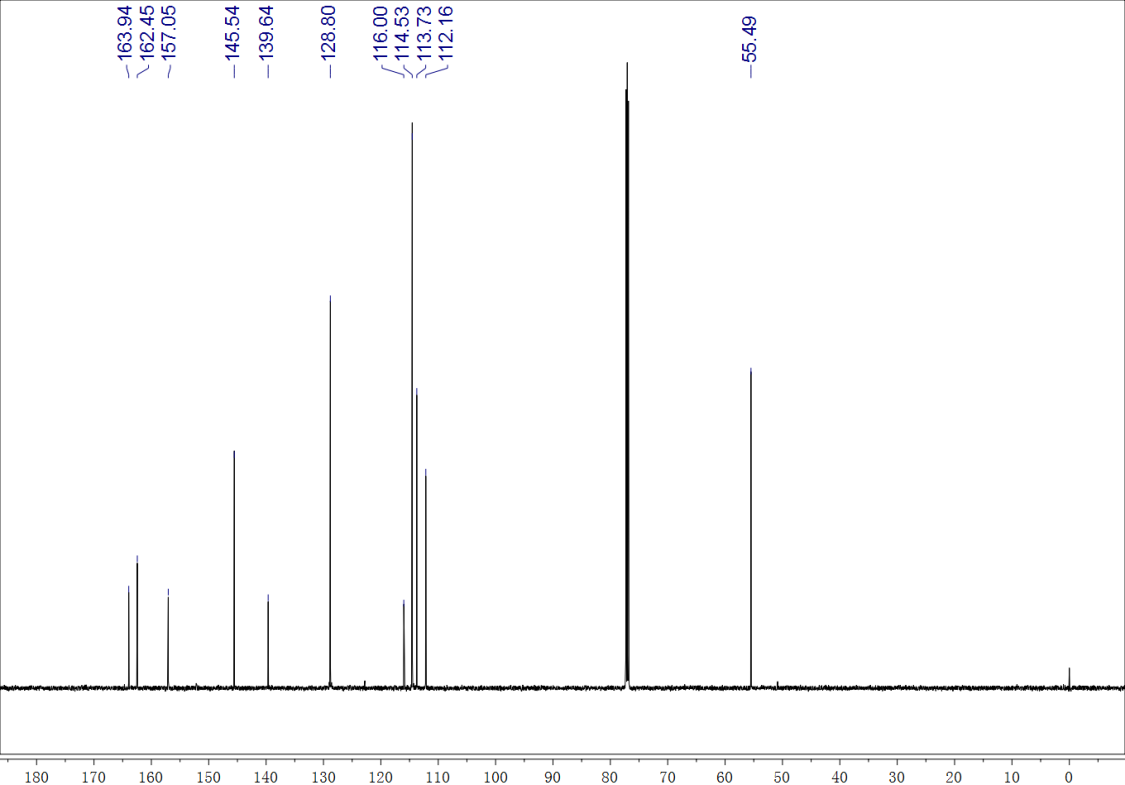


**Figure S22.** ^13^C-NMR spectrum of compound **4k**

**
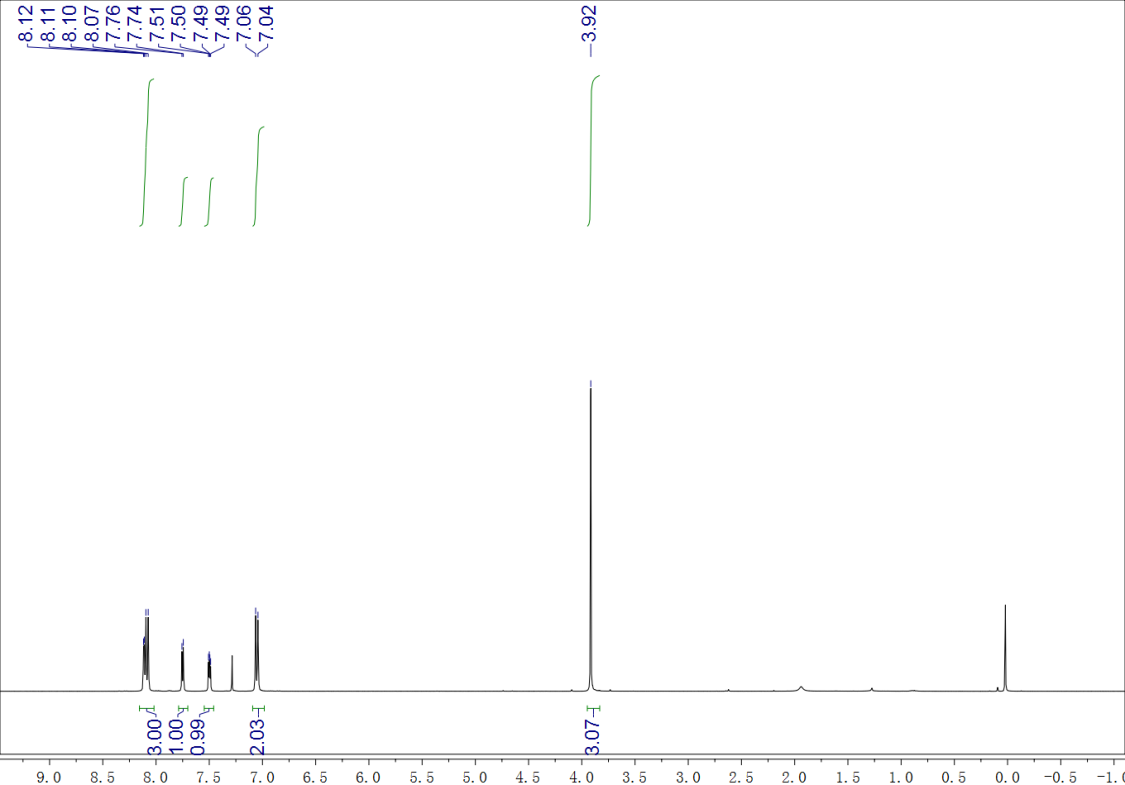
**

**Figure S23.** ^1^H-NMR spectrum of compound **4l**

**
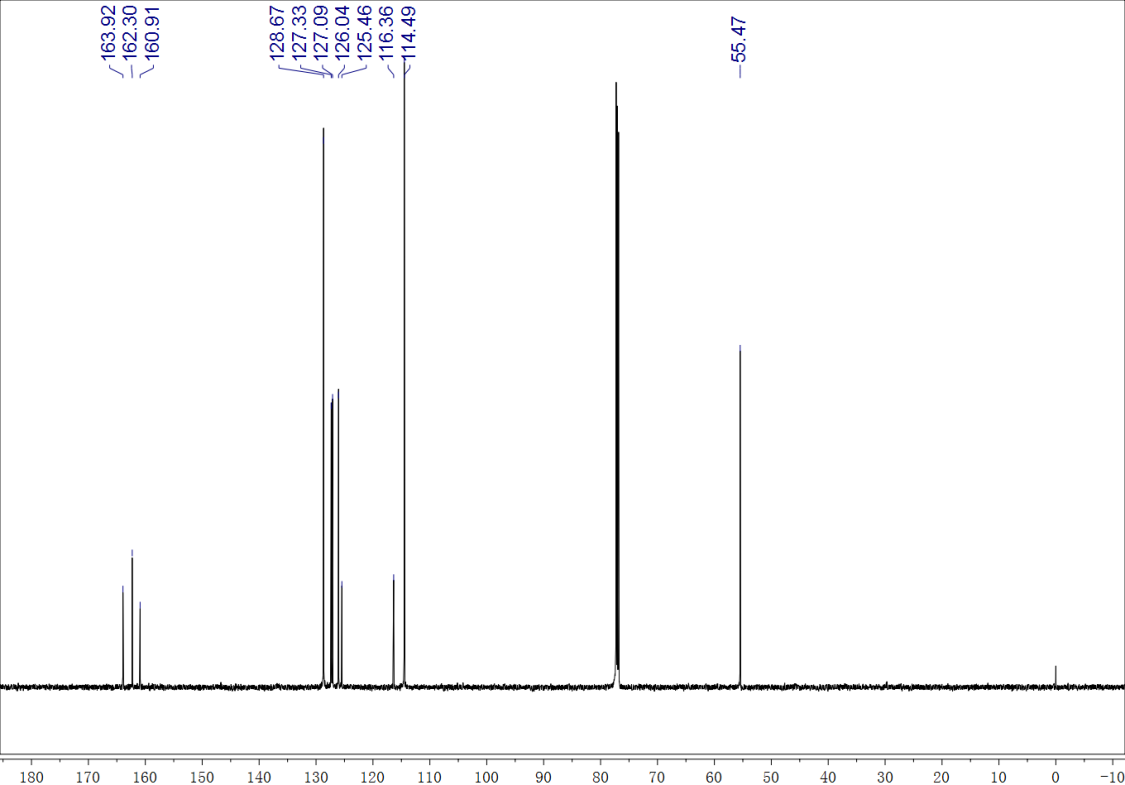
**

**Figure S24.** ^13^C-NMR spectrum of compound **4l**


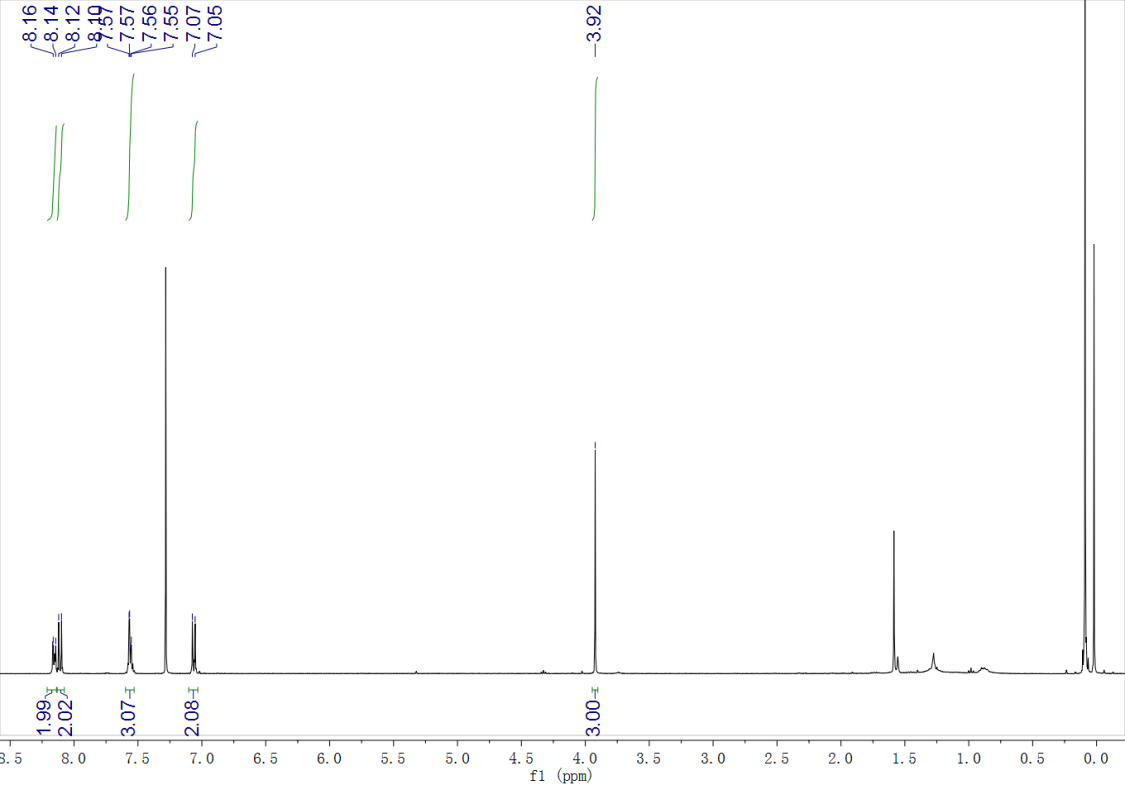


**Figure S25.** ^1^H-NMR spectrum of compound **5a**


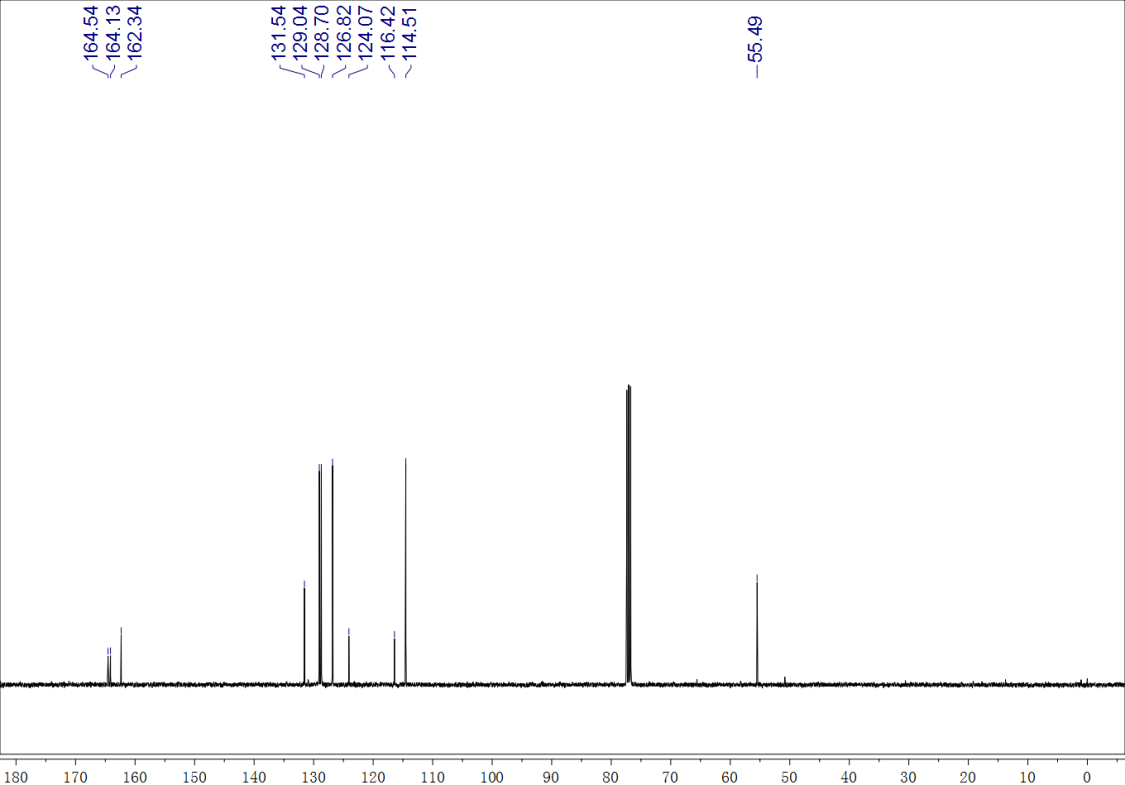


**Figure S26.** ^13^C-NMR spectrum of compound **5a**


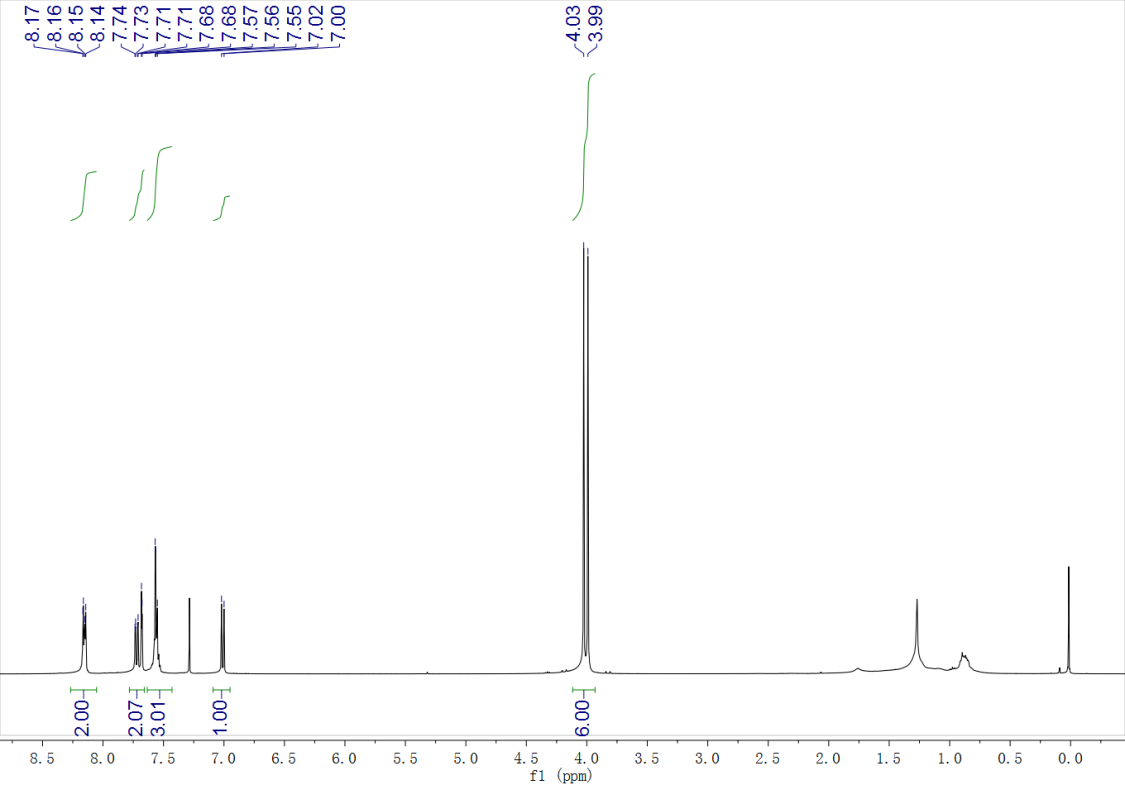


**Figure S27.** ^1^H-NMR spectrum of compound **5b**


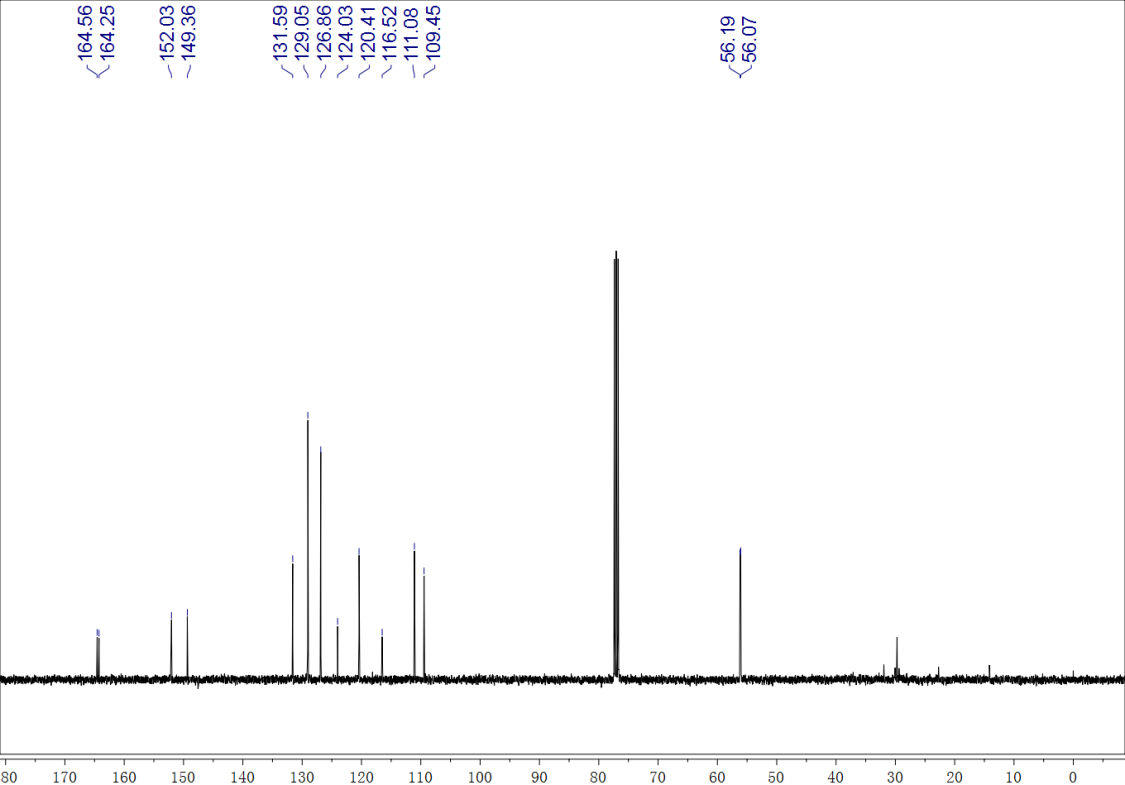


**Figure S28.** ^13^C-NMR spectrum of compound **5b**


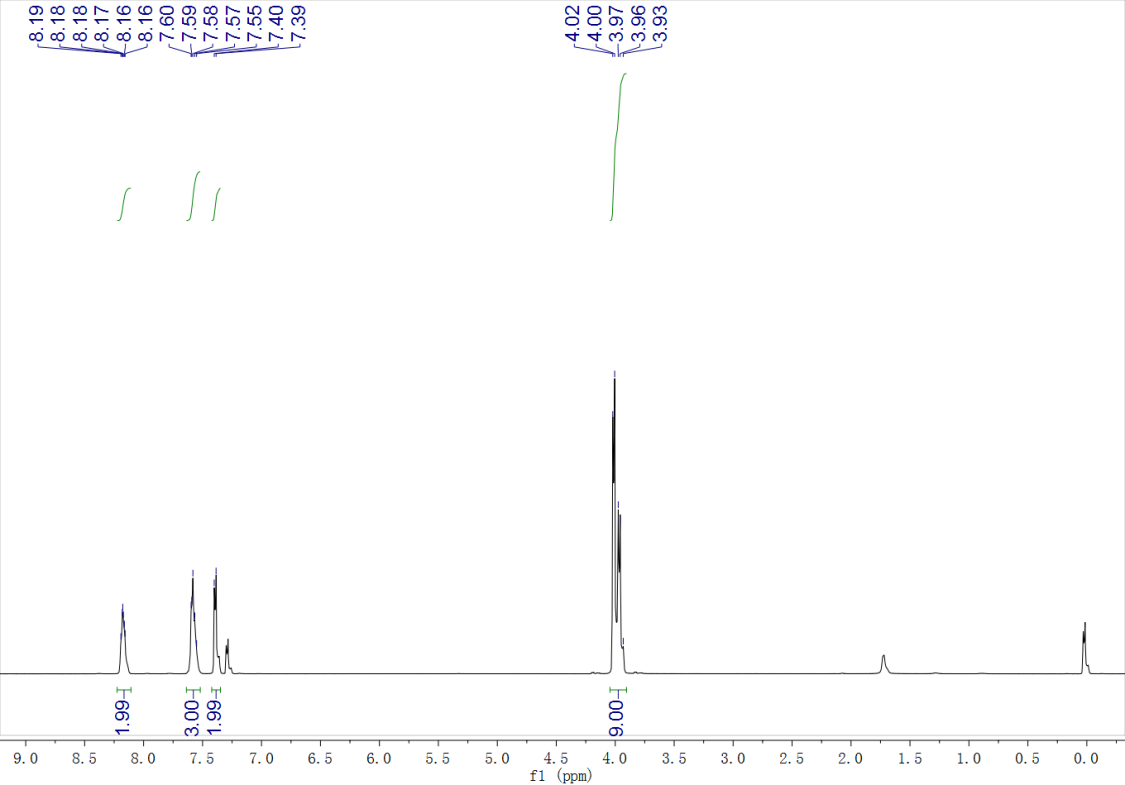


**Figure S29.** ^1^H-NMR spectrum of compound **5c**


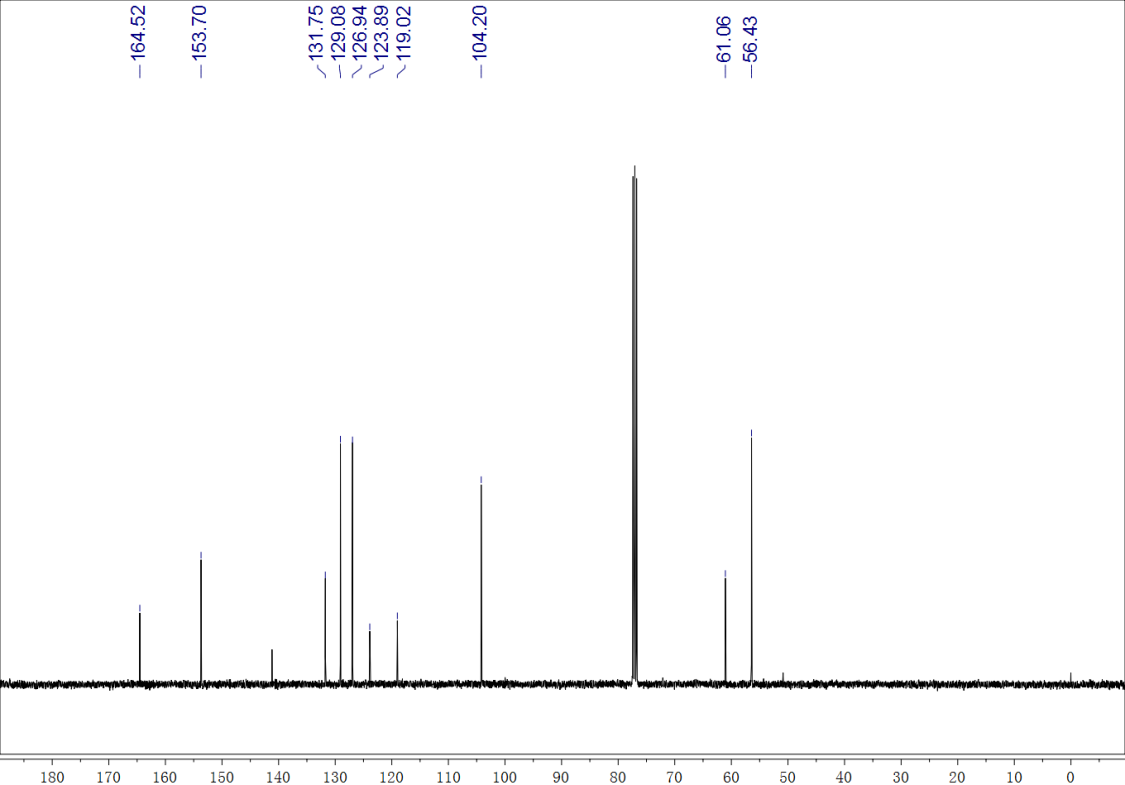


**Figure S30.** ^13^C-NMR spectrum of compound **5c**


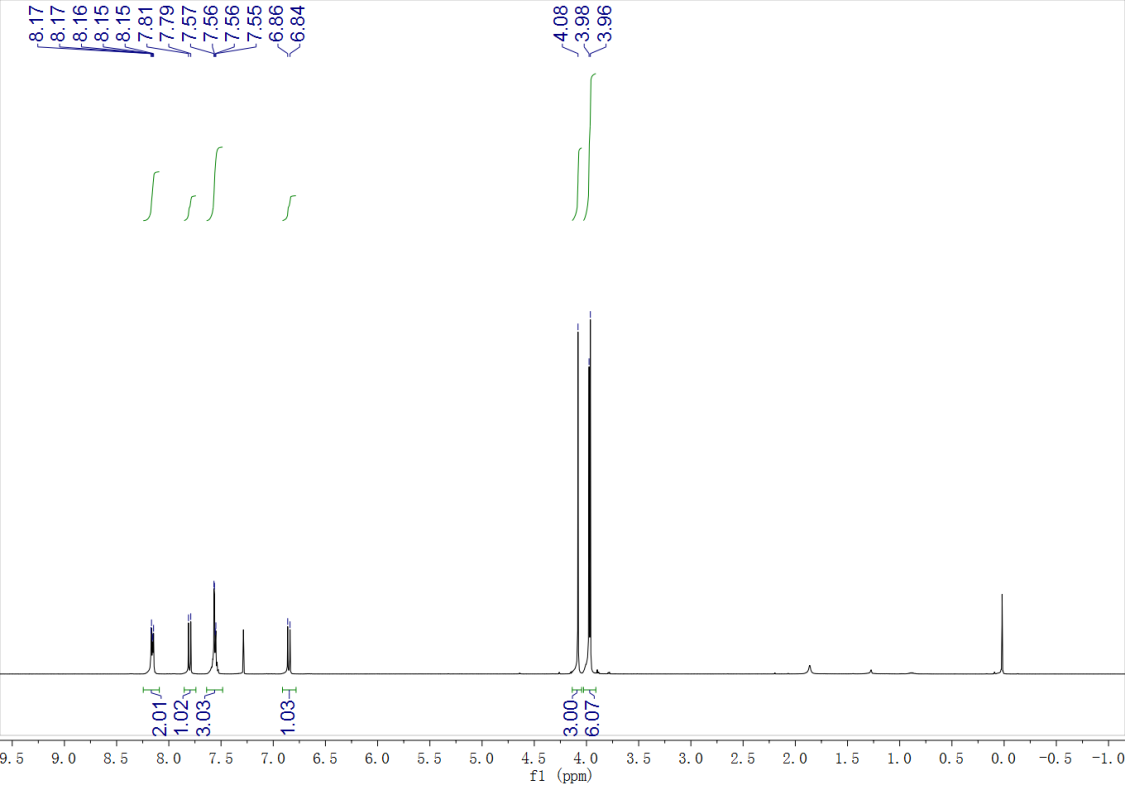


**Figure S31.** ^1^H-NMR spectrum of compound **5d**


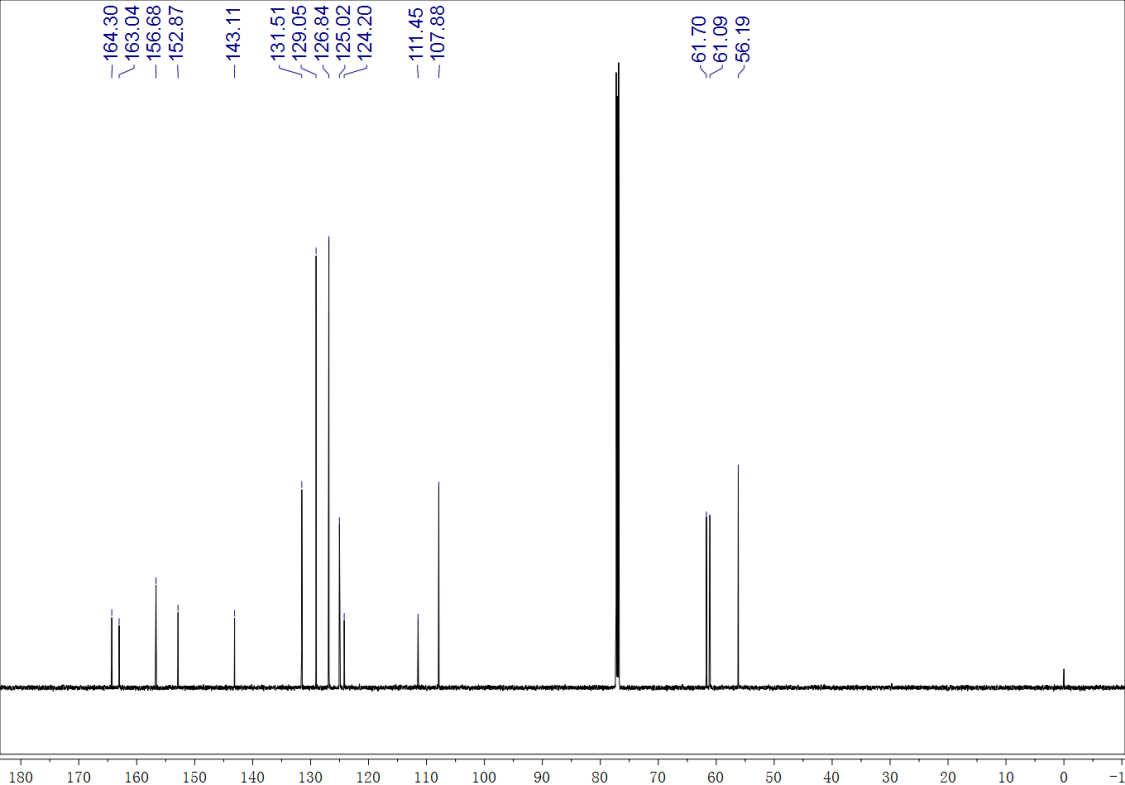


**Figure S32** ^13^C-NMR spectrum of compound **5d**


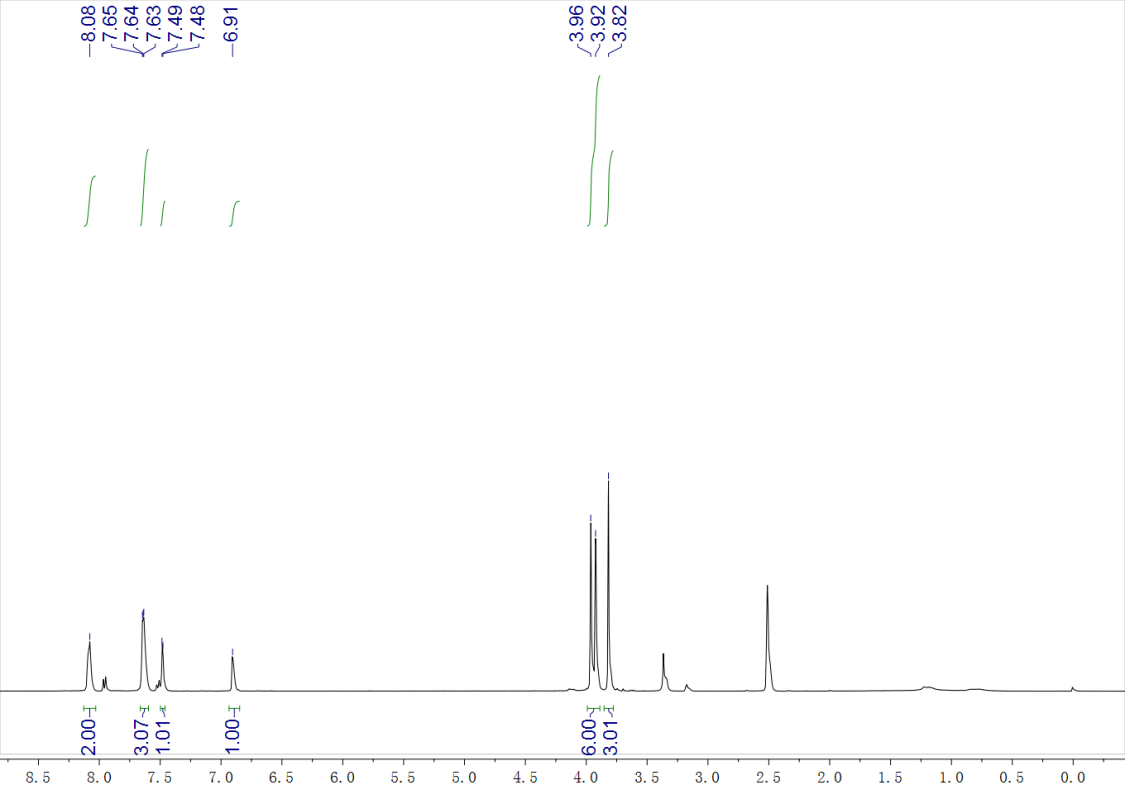


**Figure S33.** ^1^H-NMR spectrum of compound **5e**


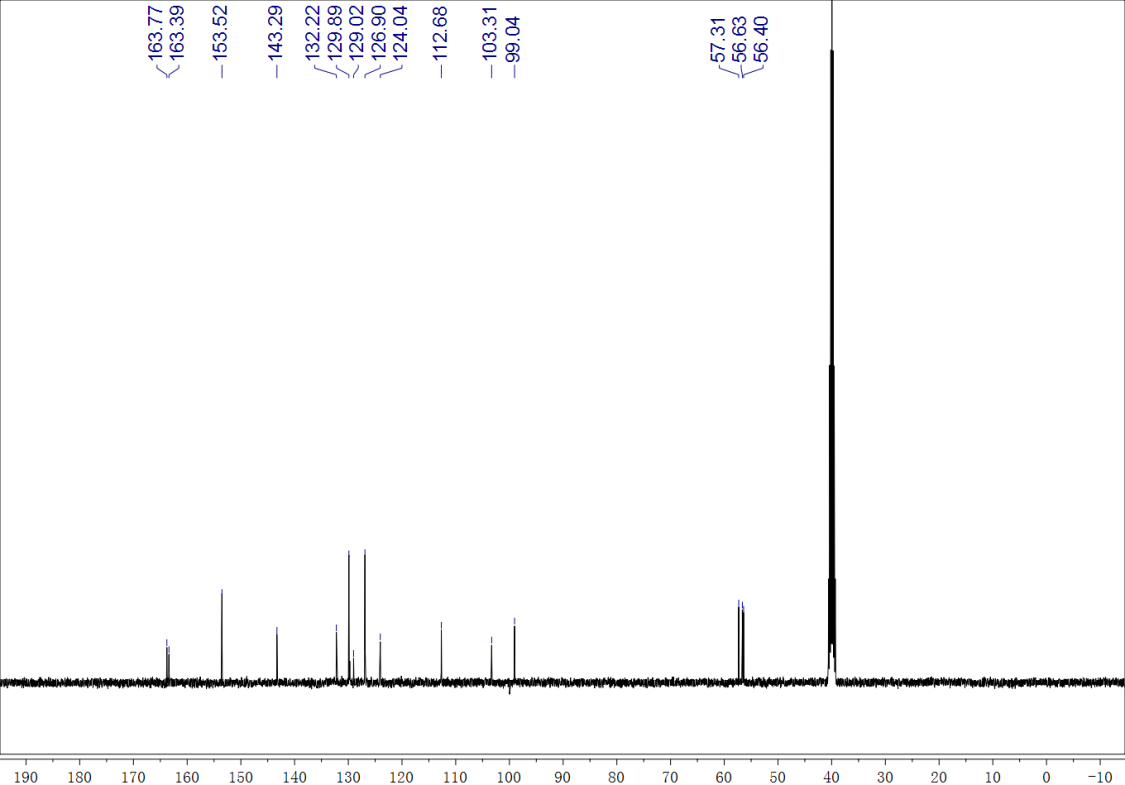


**Figure S34.** ^13^C-NMR spectrum of compound **5e**


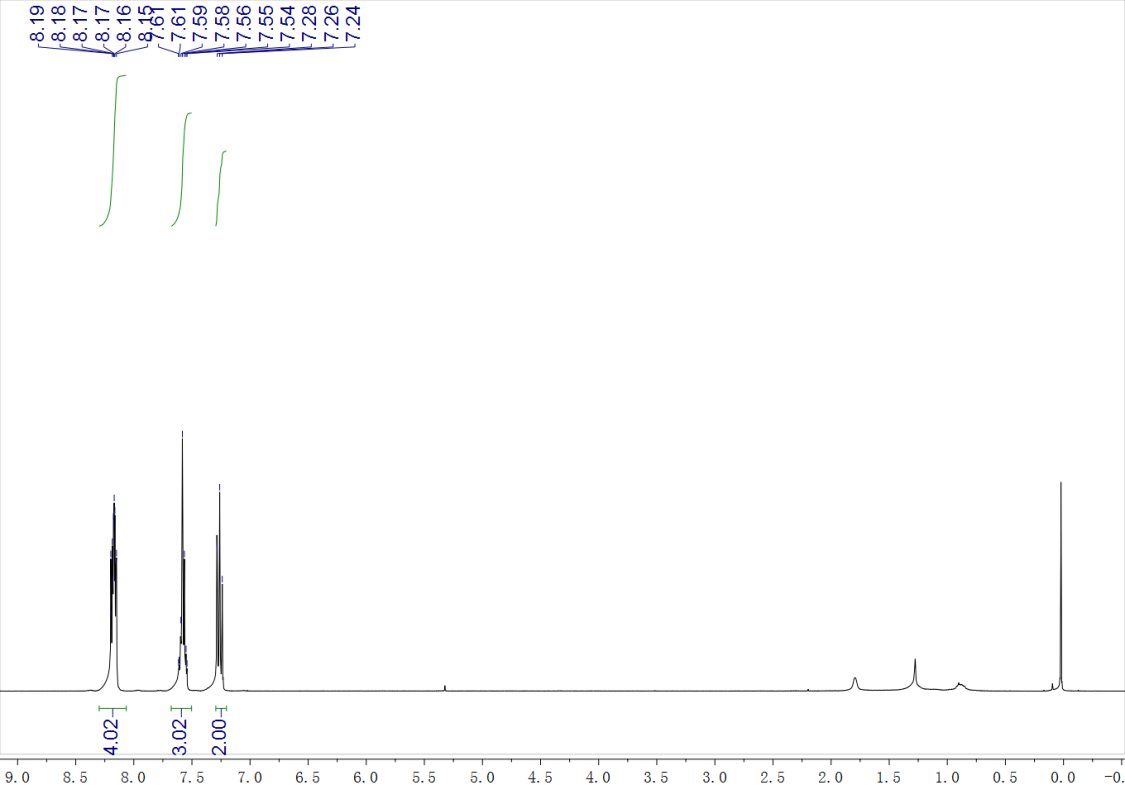


**Figure S35.** ^1^H-NMR spectrum of compound **5f**


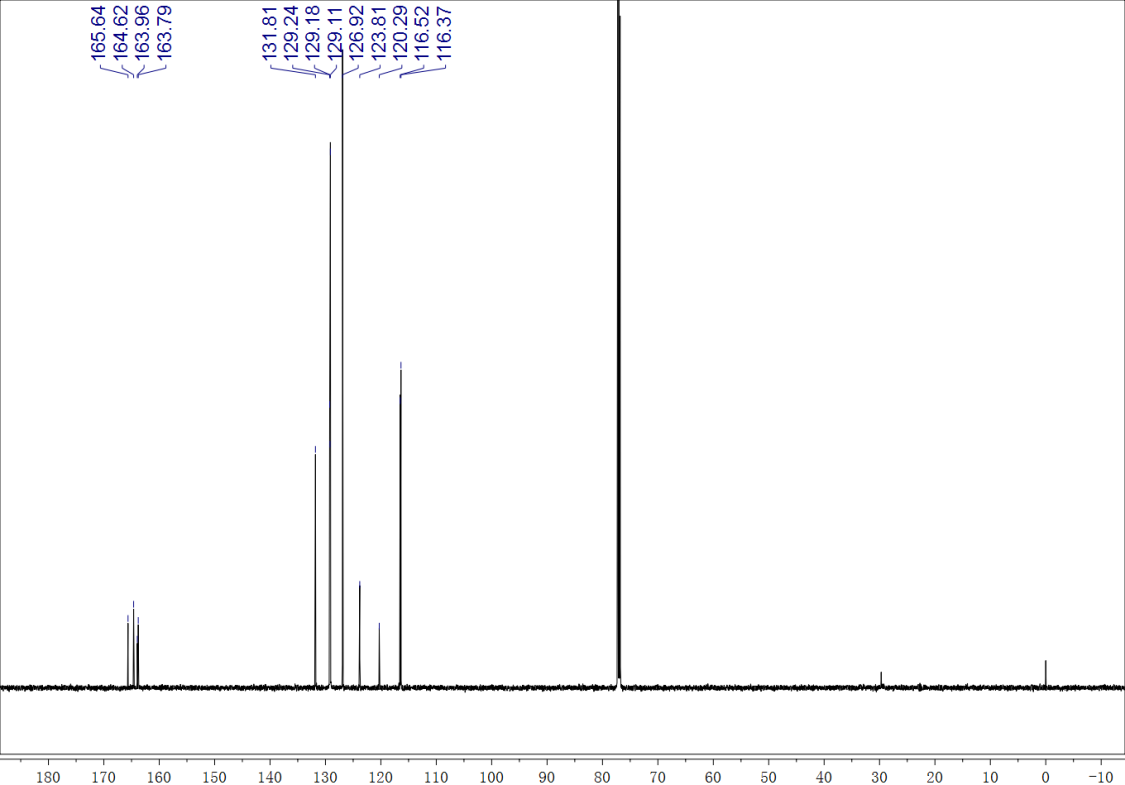


**Figure S36.** ^13^C-NMR spectrum of compound **5f**


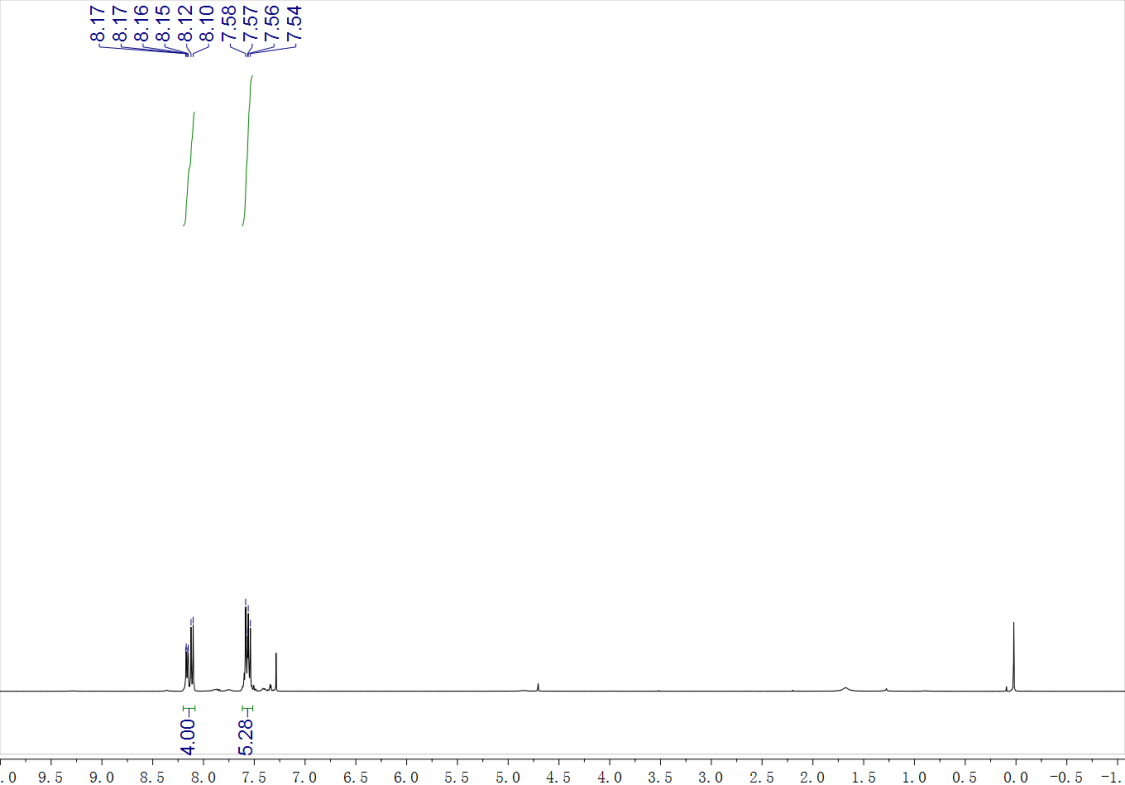


**Figure S37.** ^1^H-NMR spectrum of compound **5g**


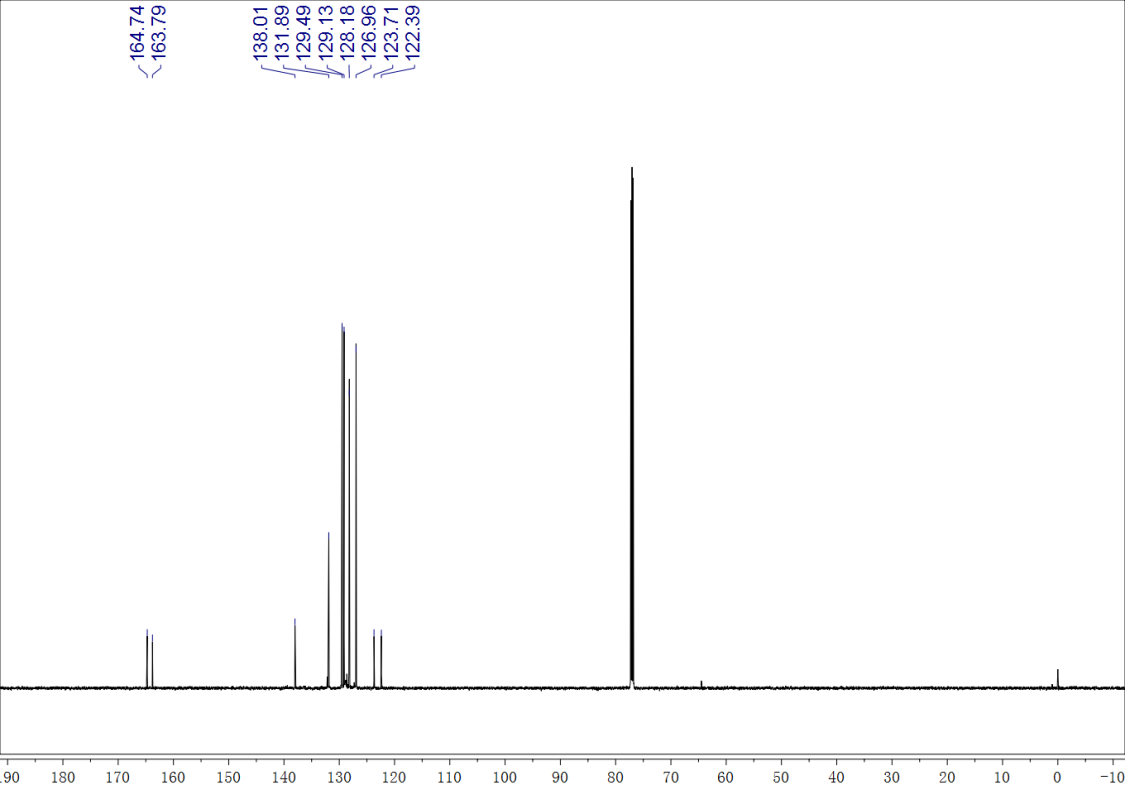


**Figure S38.** ^13^C-NMR spectrum of compound **5g**


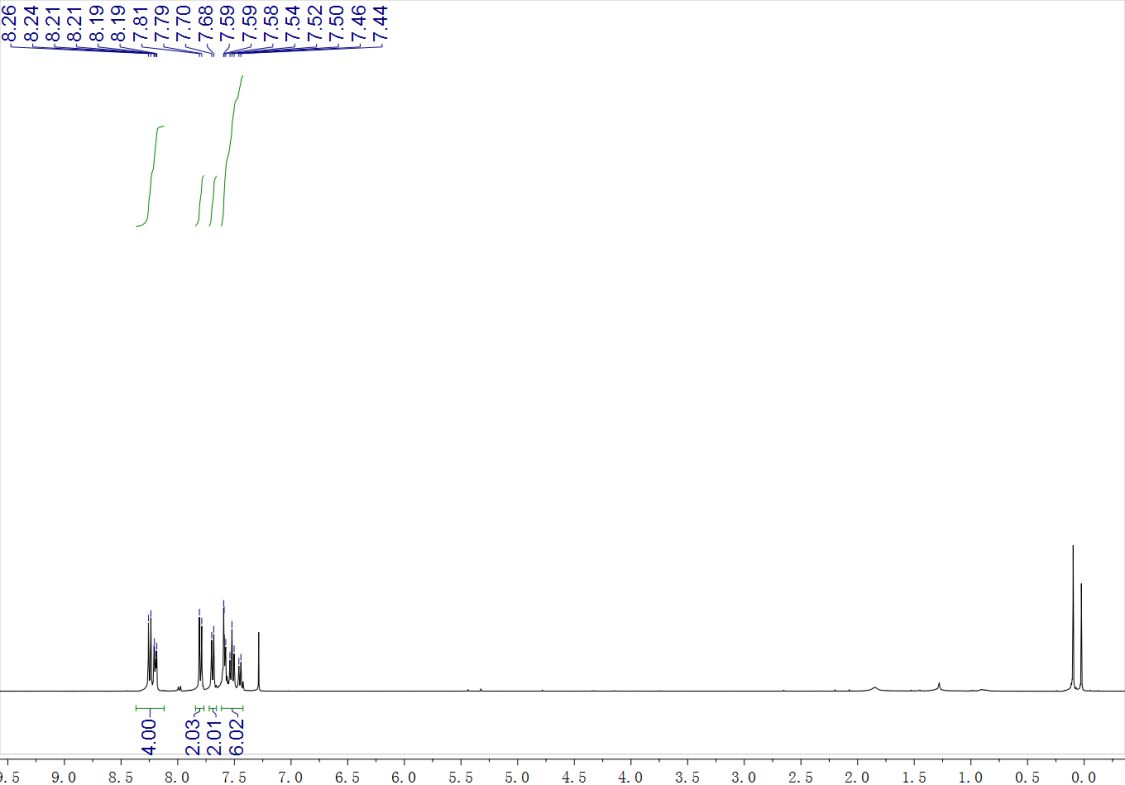


**Figure S39.** ^1^H-NMR spectrum of compound **5h**


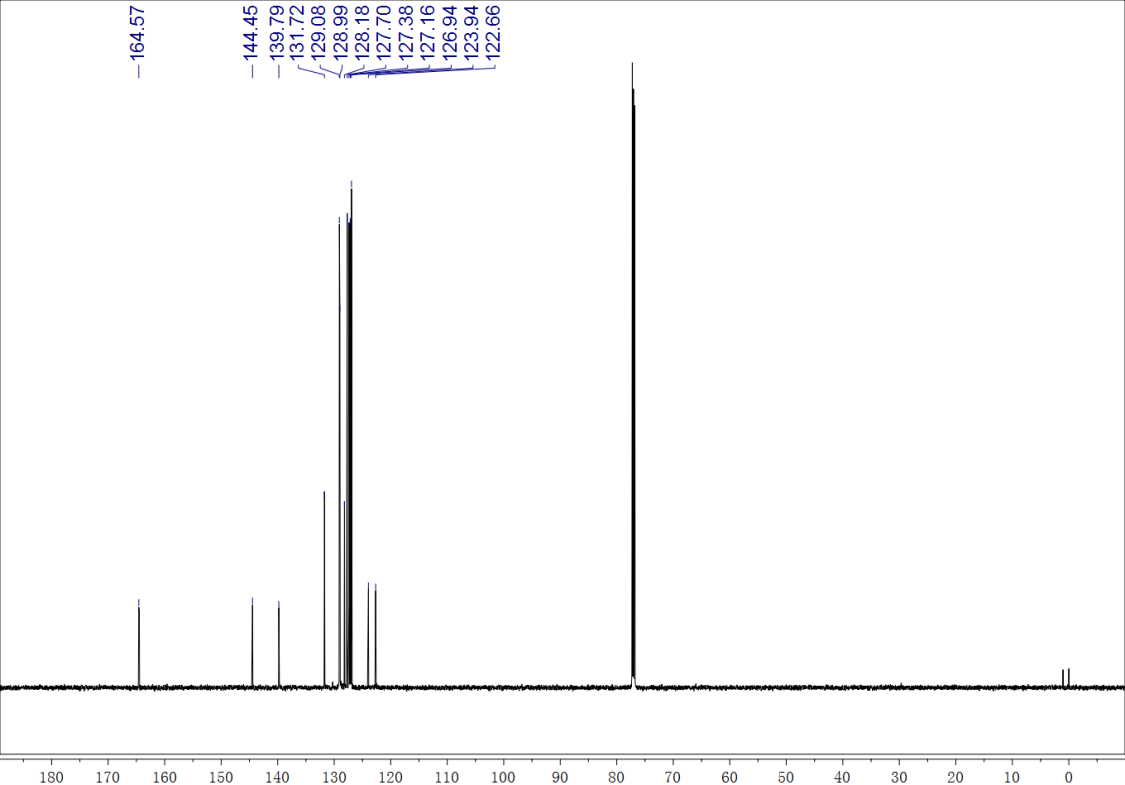


**Figure S40.** ^13^C-NMR spectrum of compound **5h**


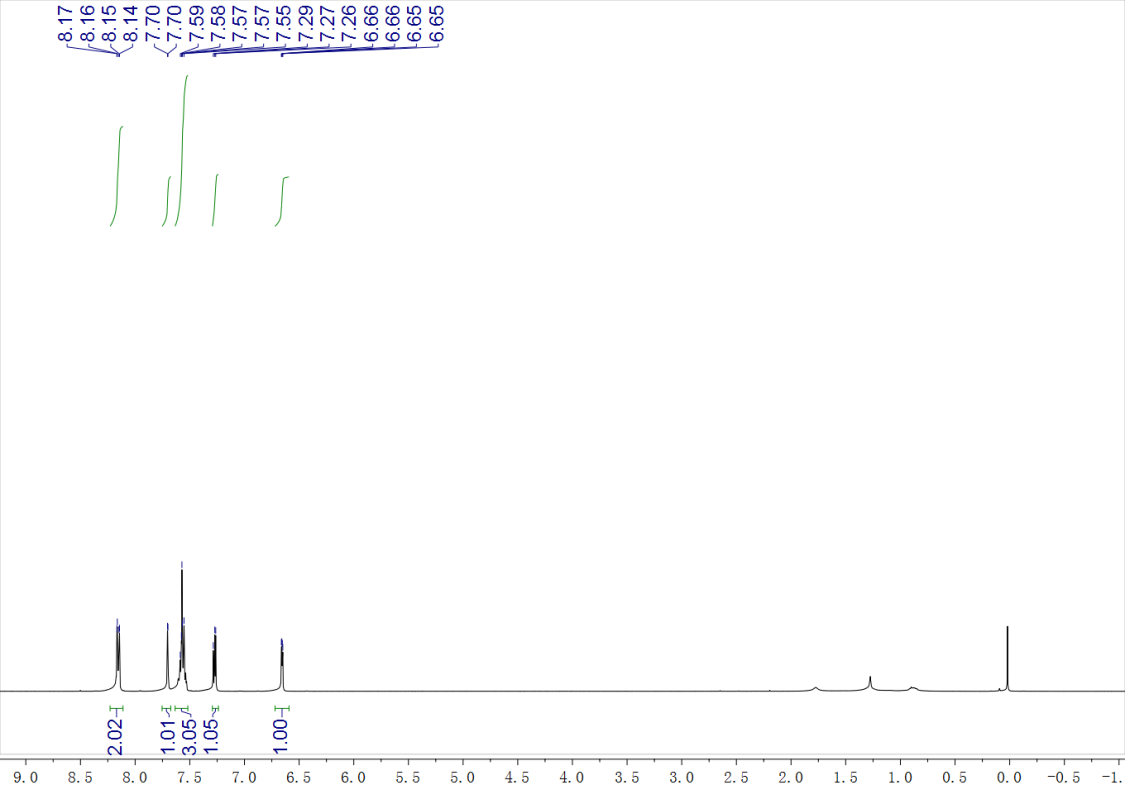


**Figure S41.** ^1^H-NMR spectrum of compound **5i**


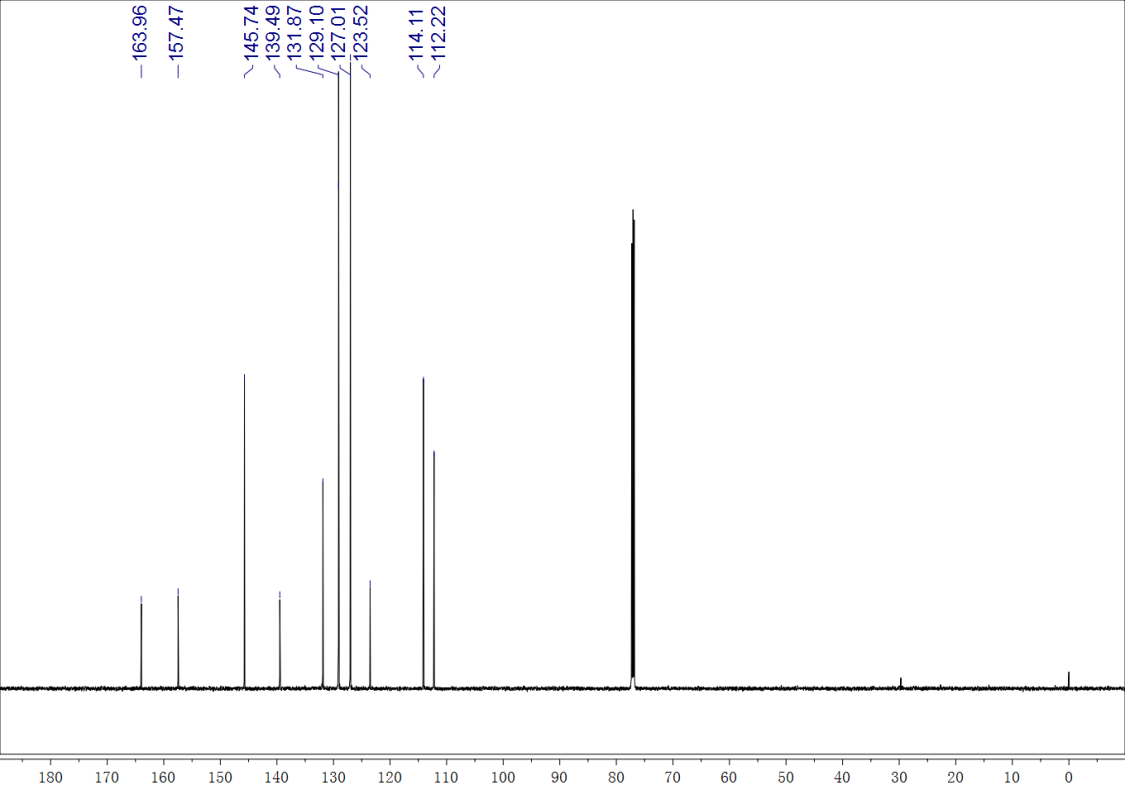


**Figure S42.** ^13^C-NMR spectrum of compound **5i**


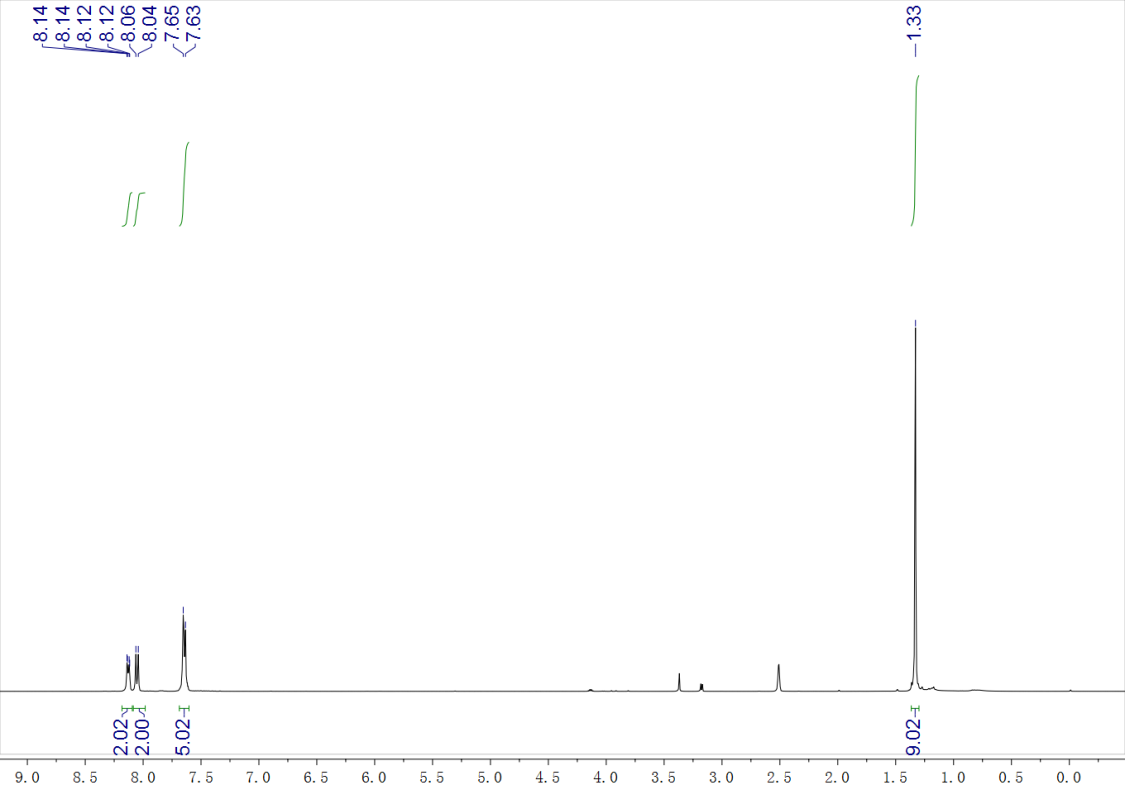


**Figure S43.** ^1^H-NMR spectrum of compound **5j**


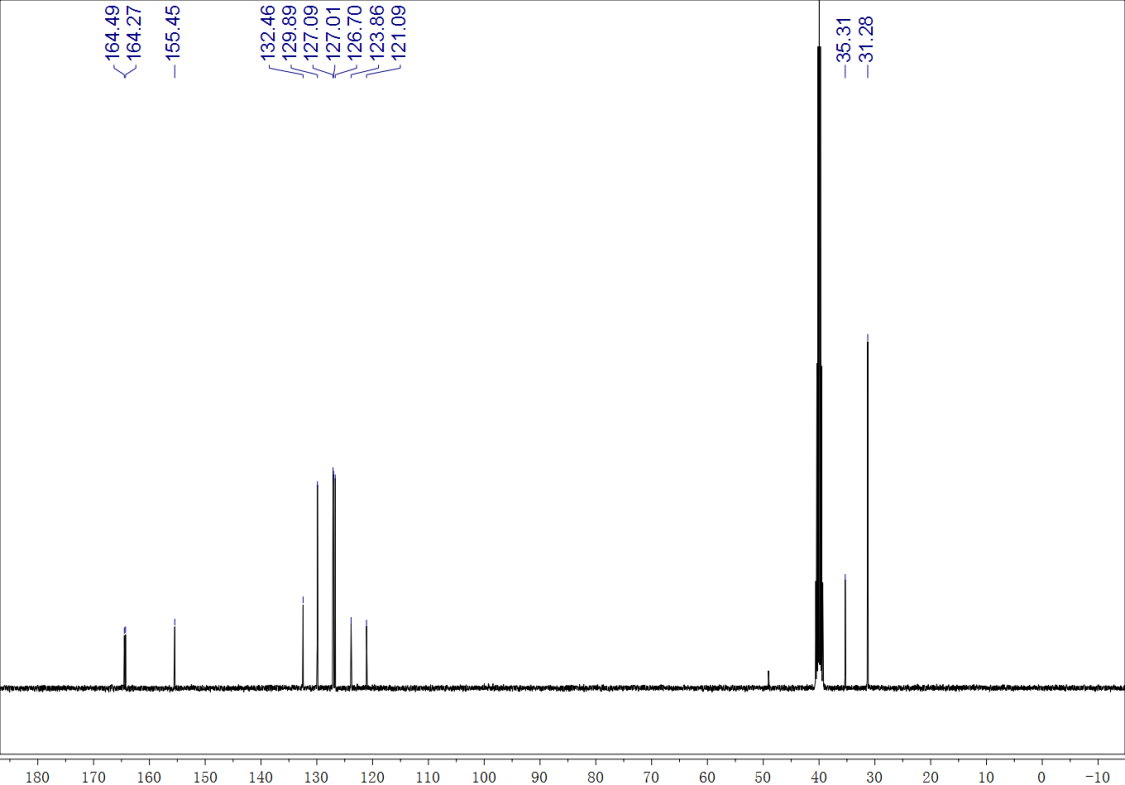


**Figure S44.** ^13^C-NMR spectrum of compound **5j**


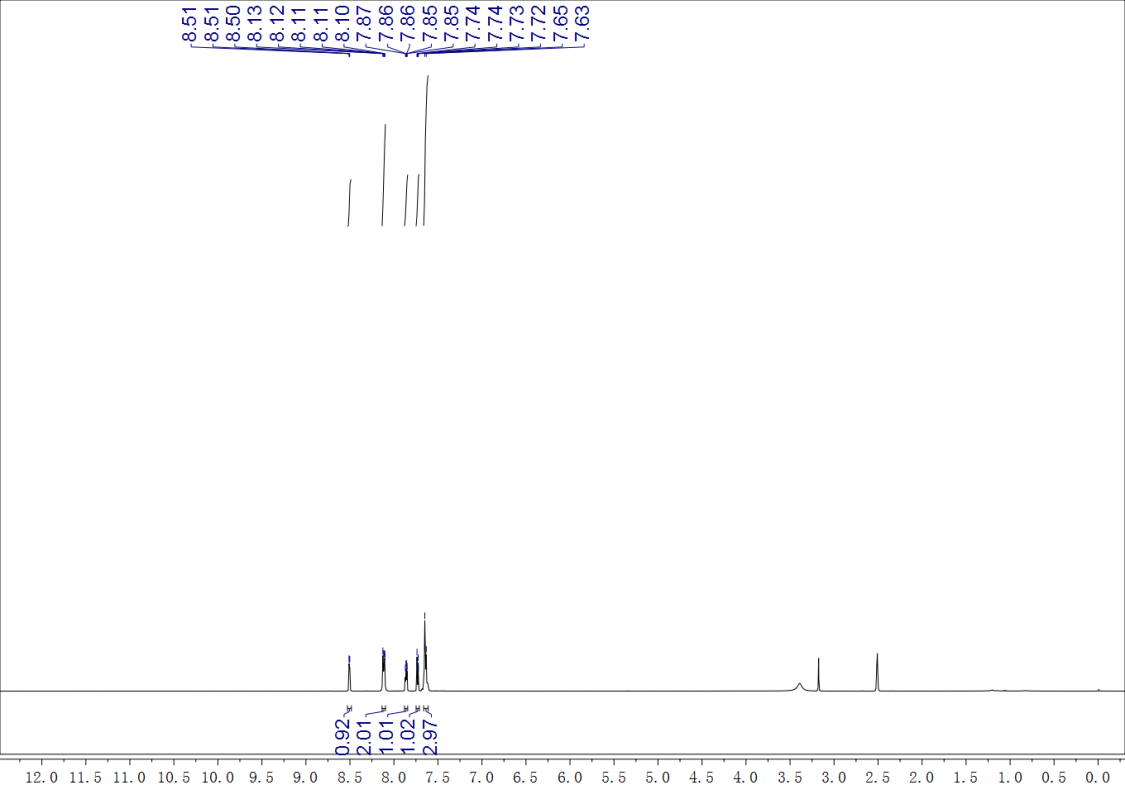


**Figure S45.** ^1^H-NMR spectrum of compound **5k**


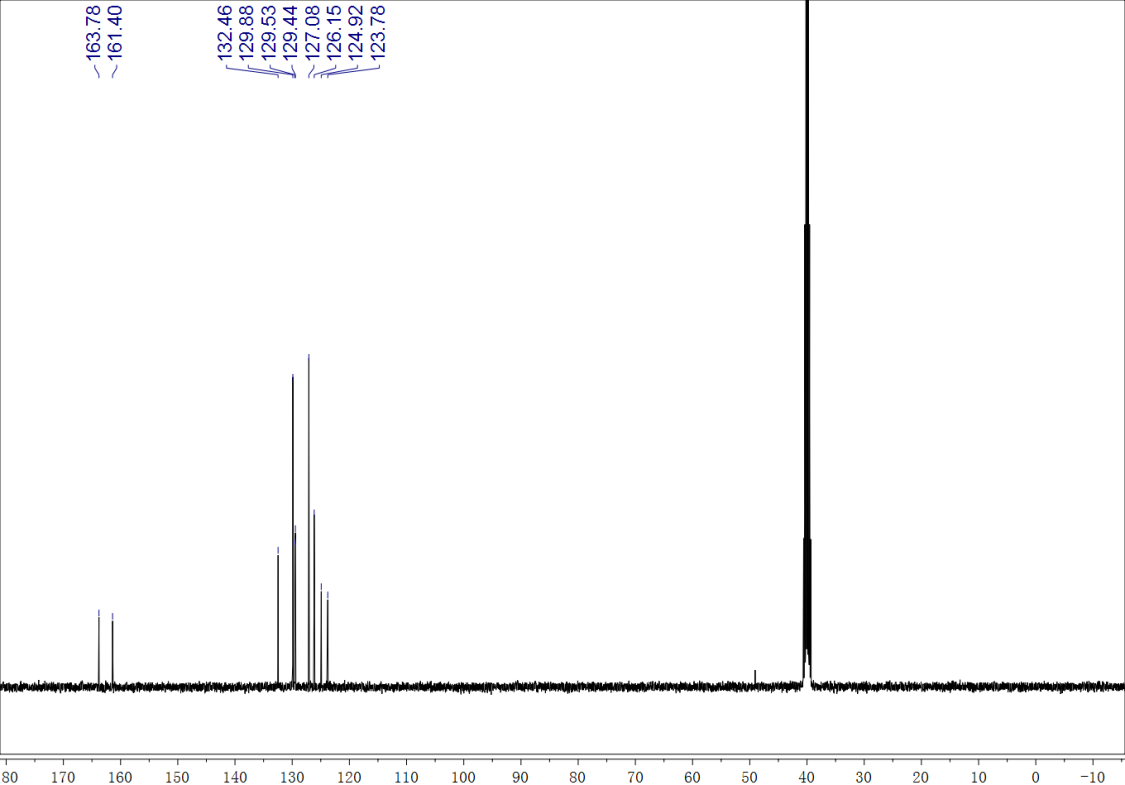


**Figure S46.** ^13^C-NMR spectrum of compound **5k**


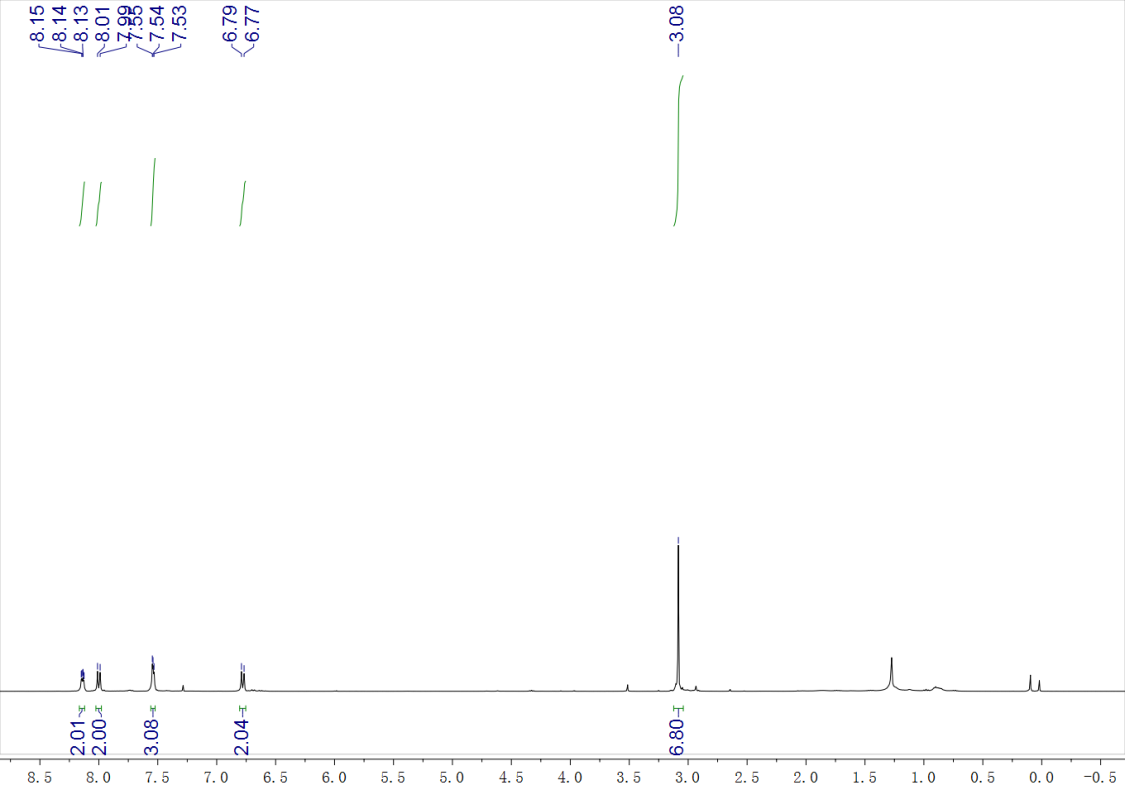


**Figure S47.** ^1^H-NMR spectrum of compound **5l**


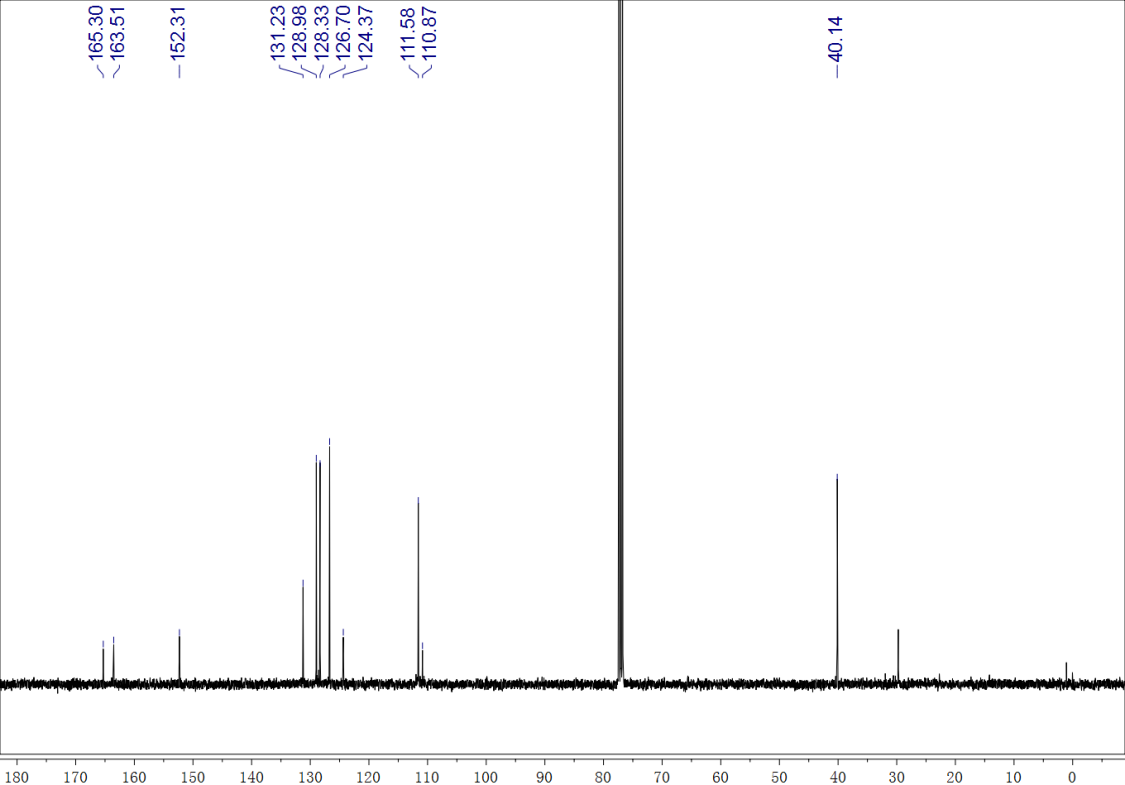


**Figure S48.** ^13^C-NMR spectrum of compound **5l**


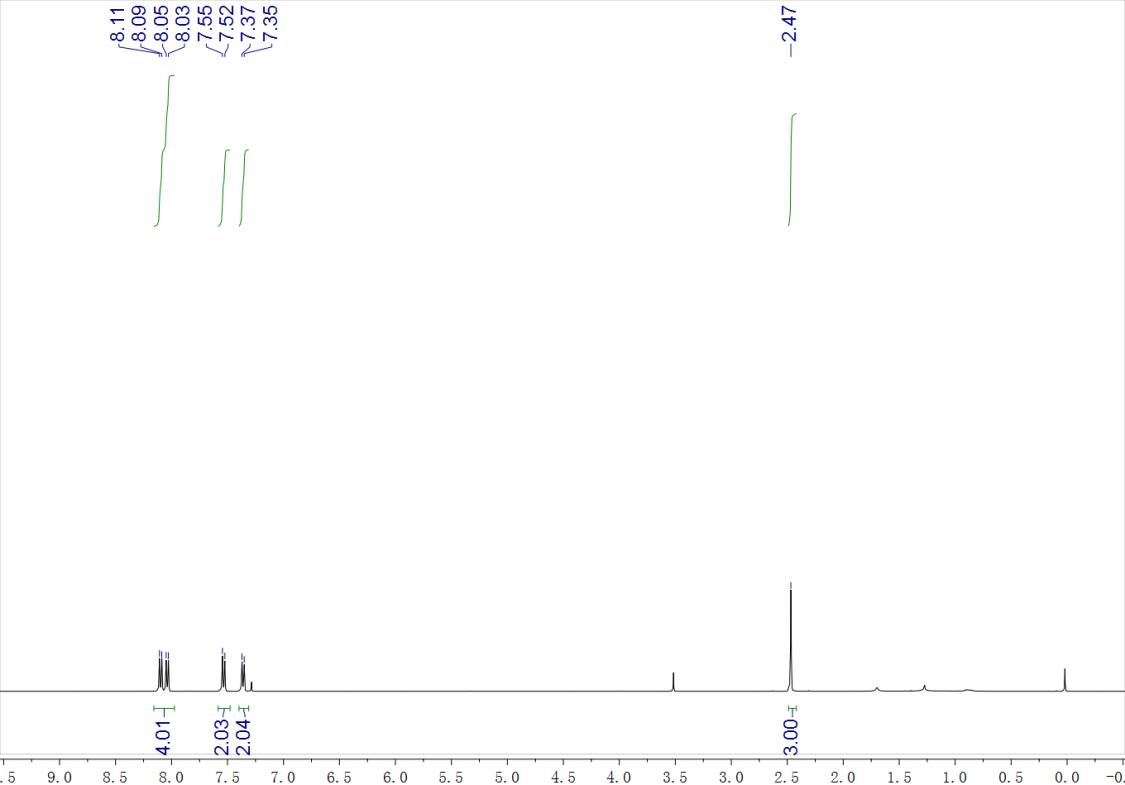


**Figure S49.** ^1^H-NMR spectrum of compound **6a**


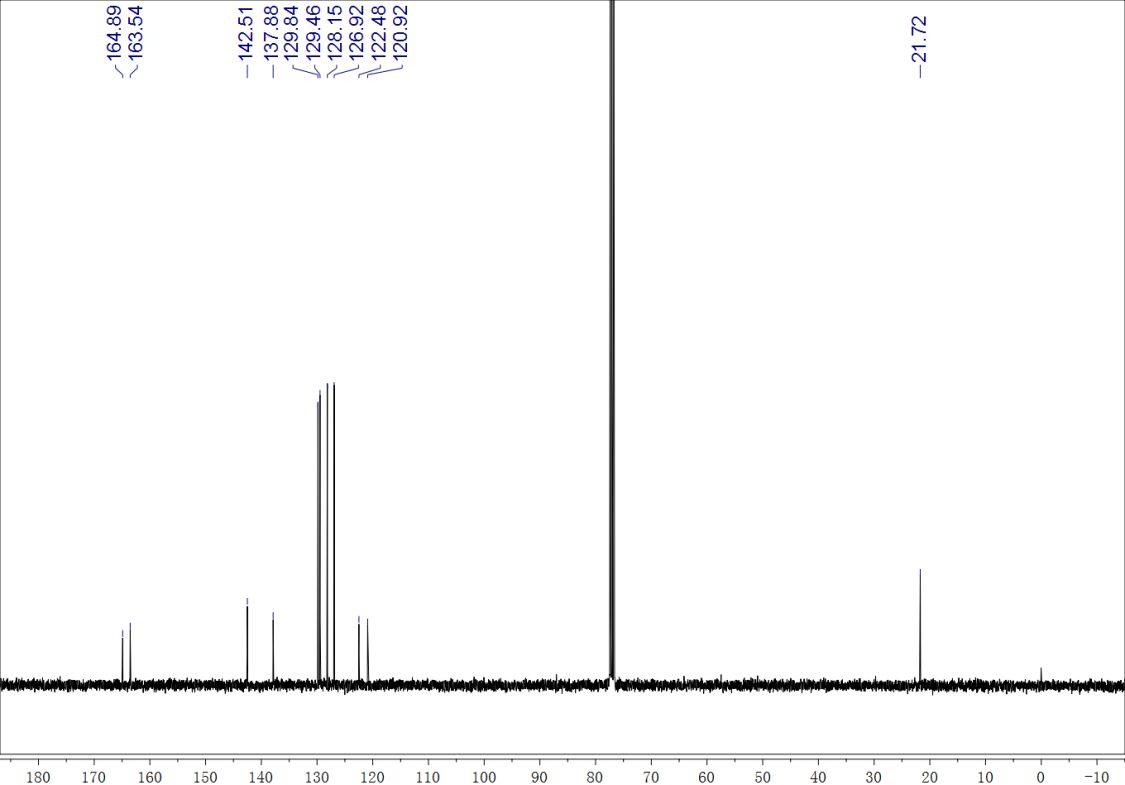


**Figure S50.** ^13^C-NMR spectrum of compound **6a**


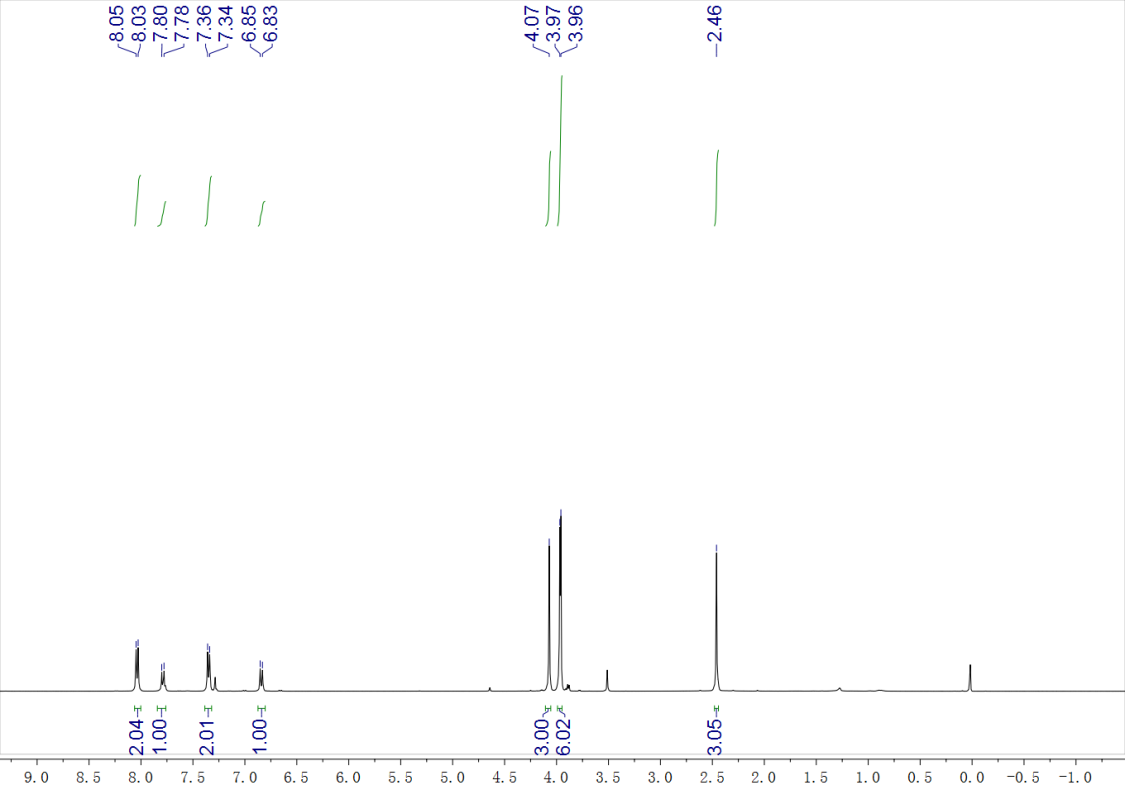


**Figure S51.** ^1^H-NMR spectrum of compound **6b**


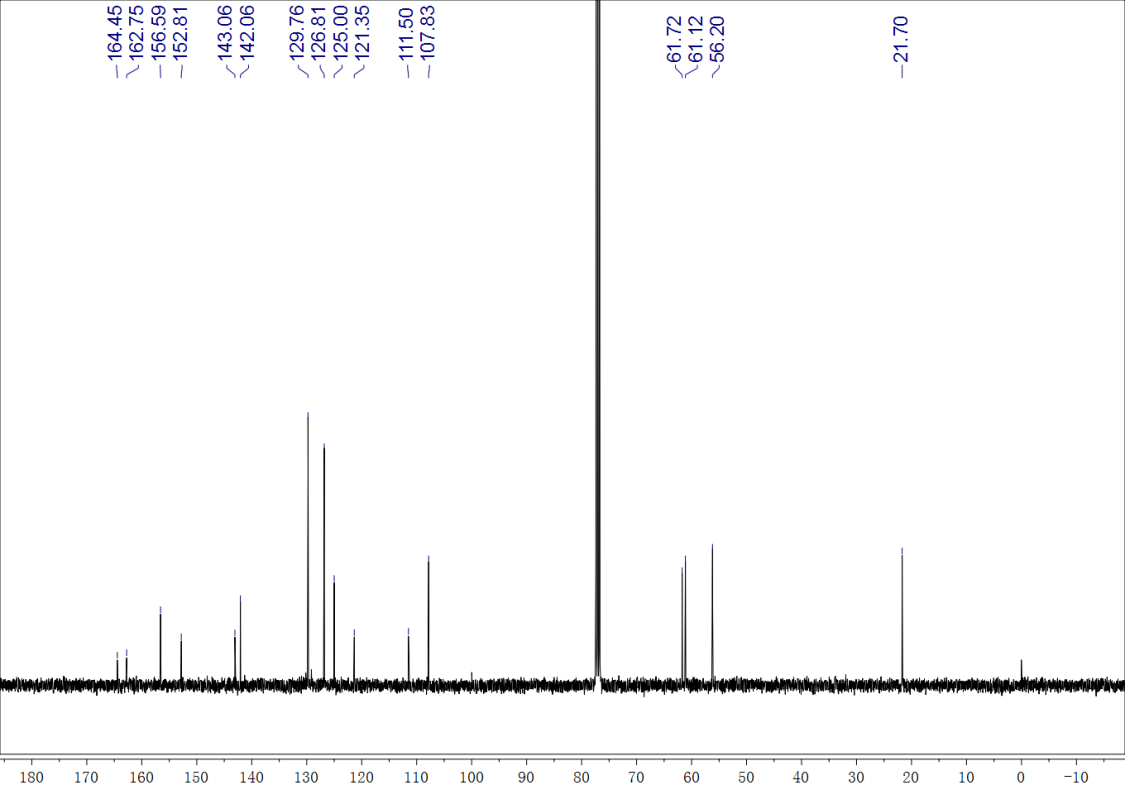


**Figure S52.** ^13^C-NMR spectrum of compound **6b**


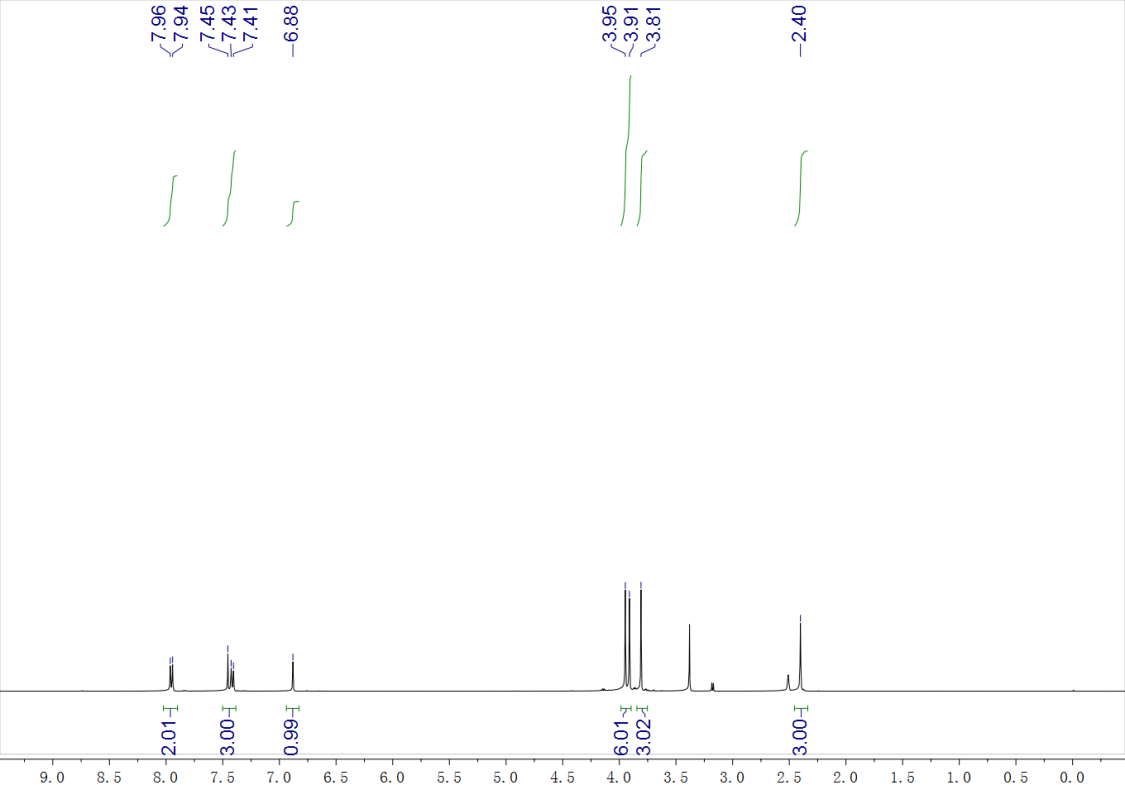


**Figure S53.** ^1^H-NMR spectrum of compound **6c**


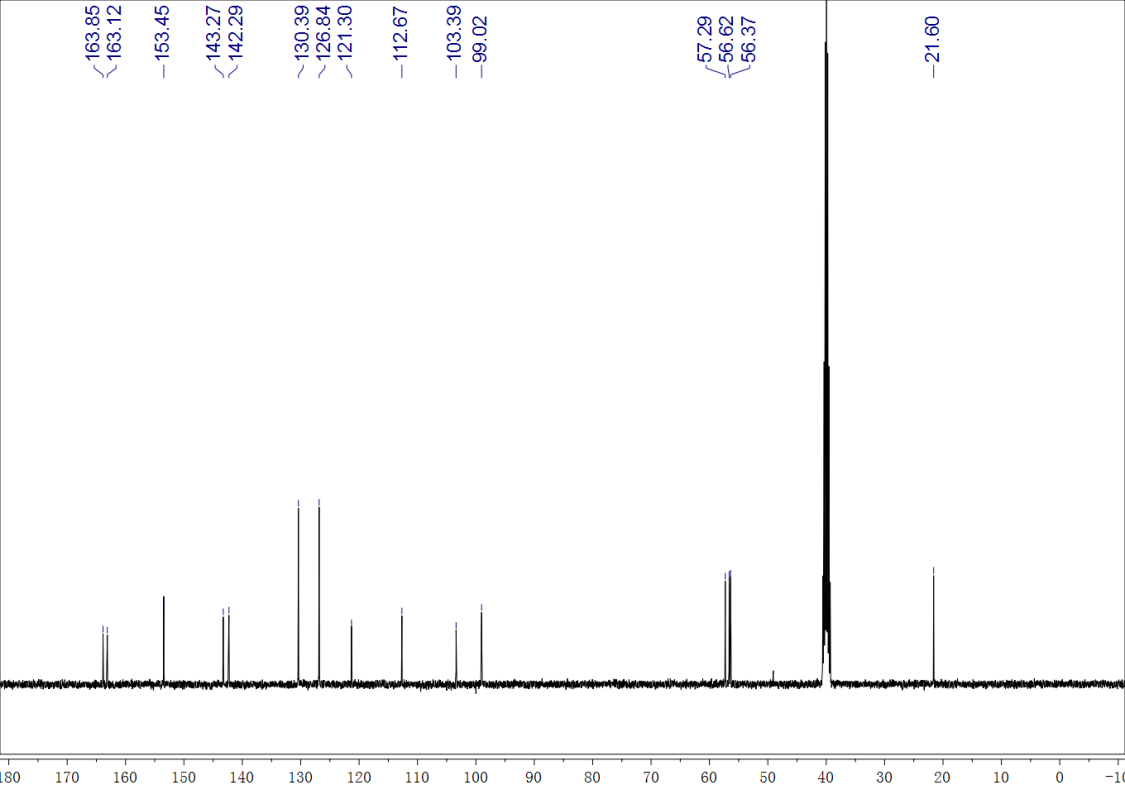


**Figure S54.** ^13^C-NMR spectrum of compound **6c**


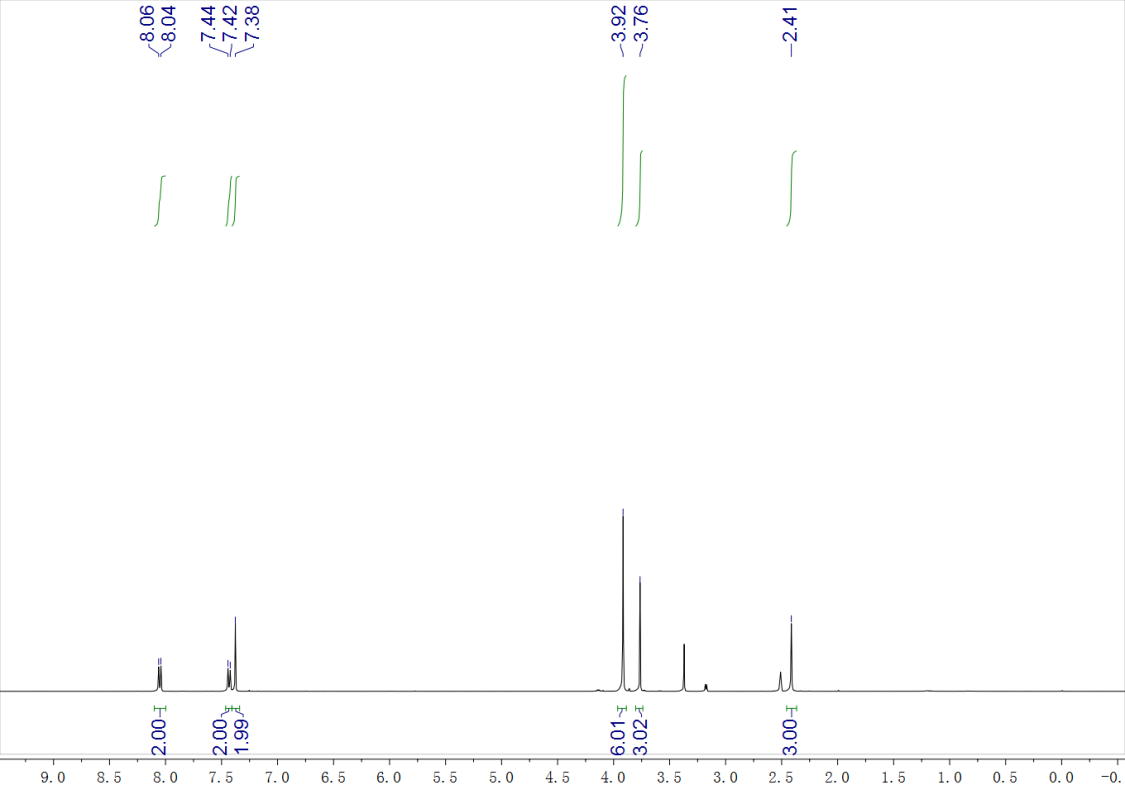


**Figure S55.** ^1^H-NMR spectrum of compound **6d**


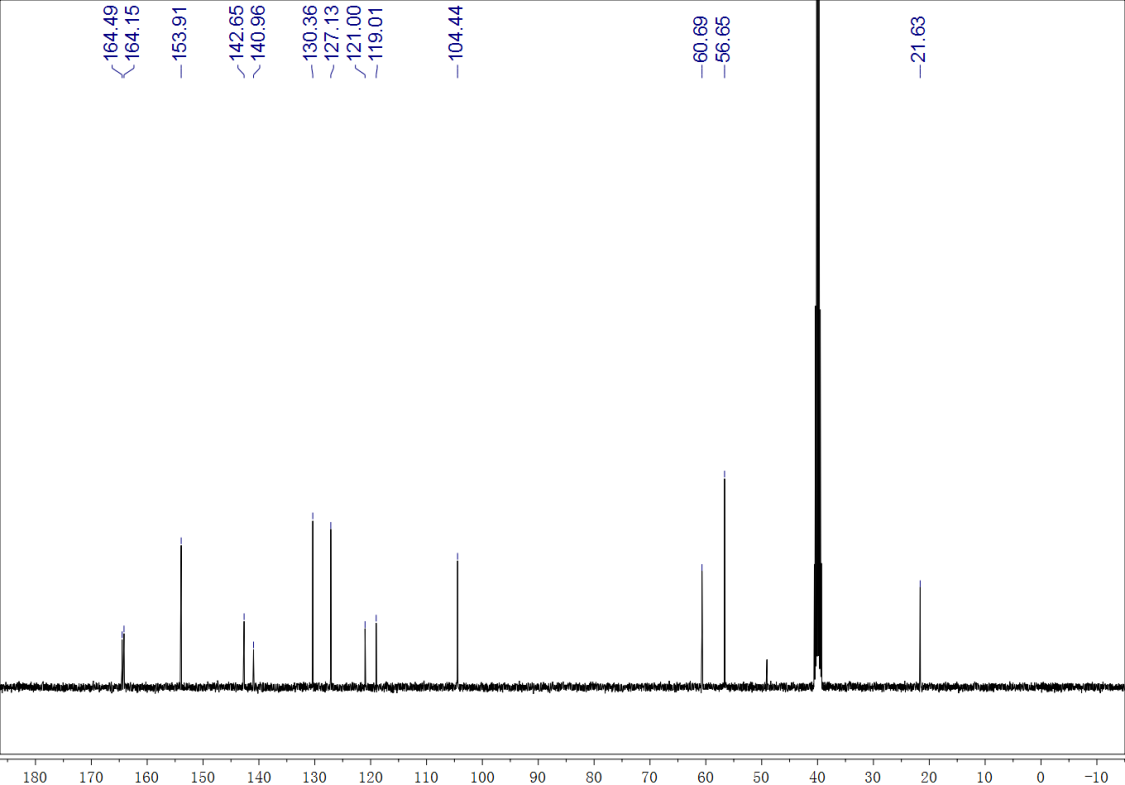


**Figure S56.** ^13^C-NMR spectrum of compound **6d**


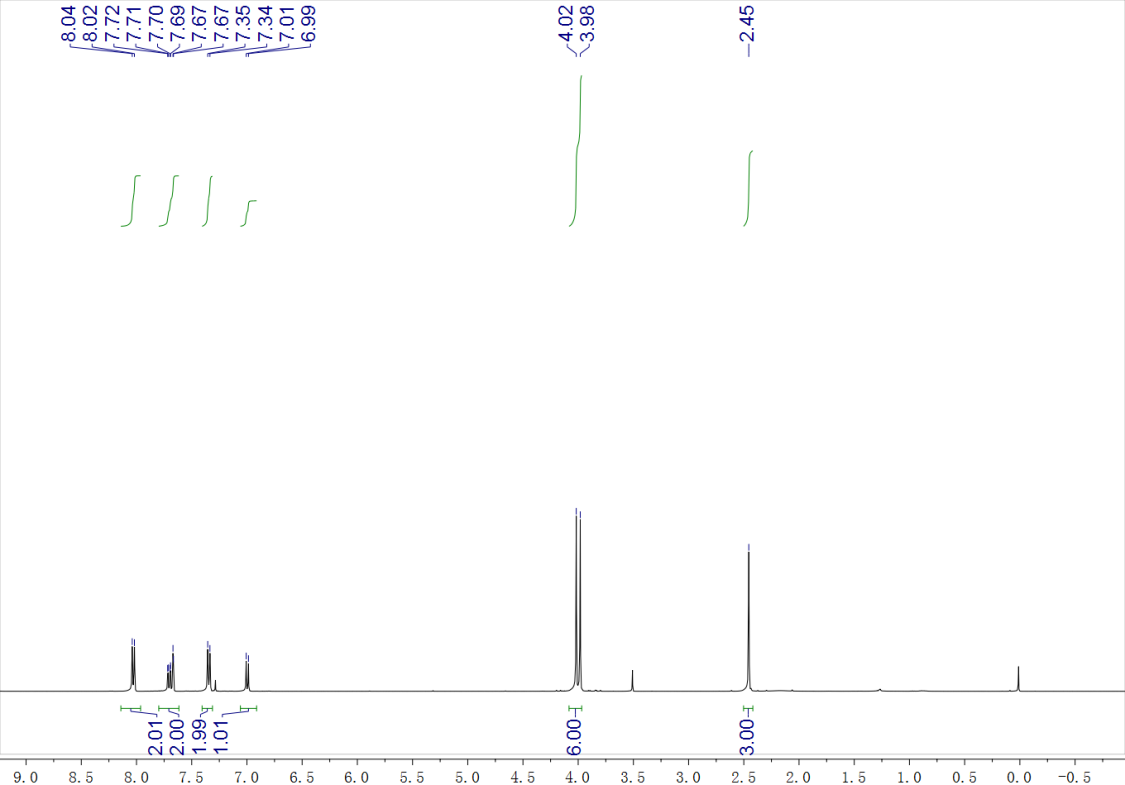


**Figure S57.** ^1^H-NMR spectrum of compound **6e**


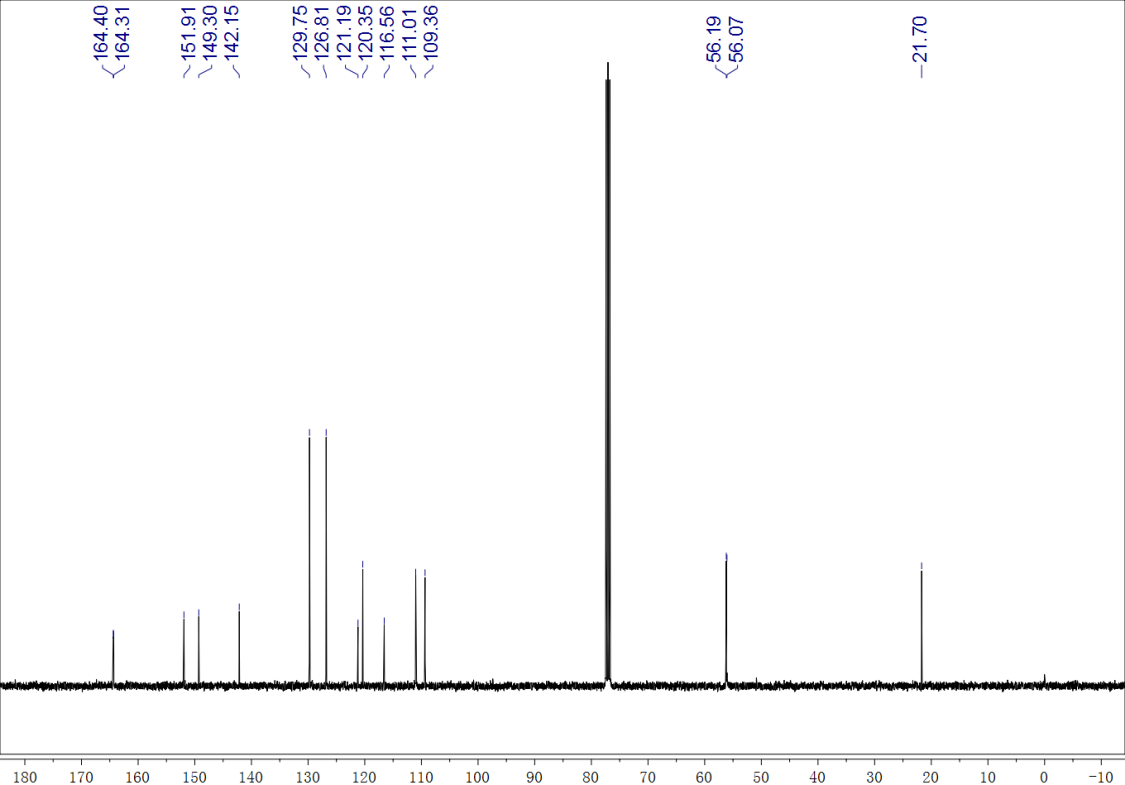


**Figure S58.** ^13^C-NMR spectrum of compound **6e**


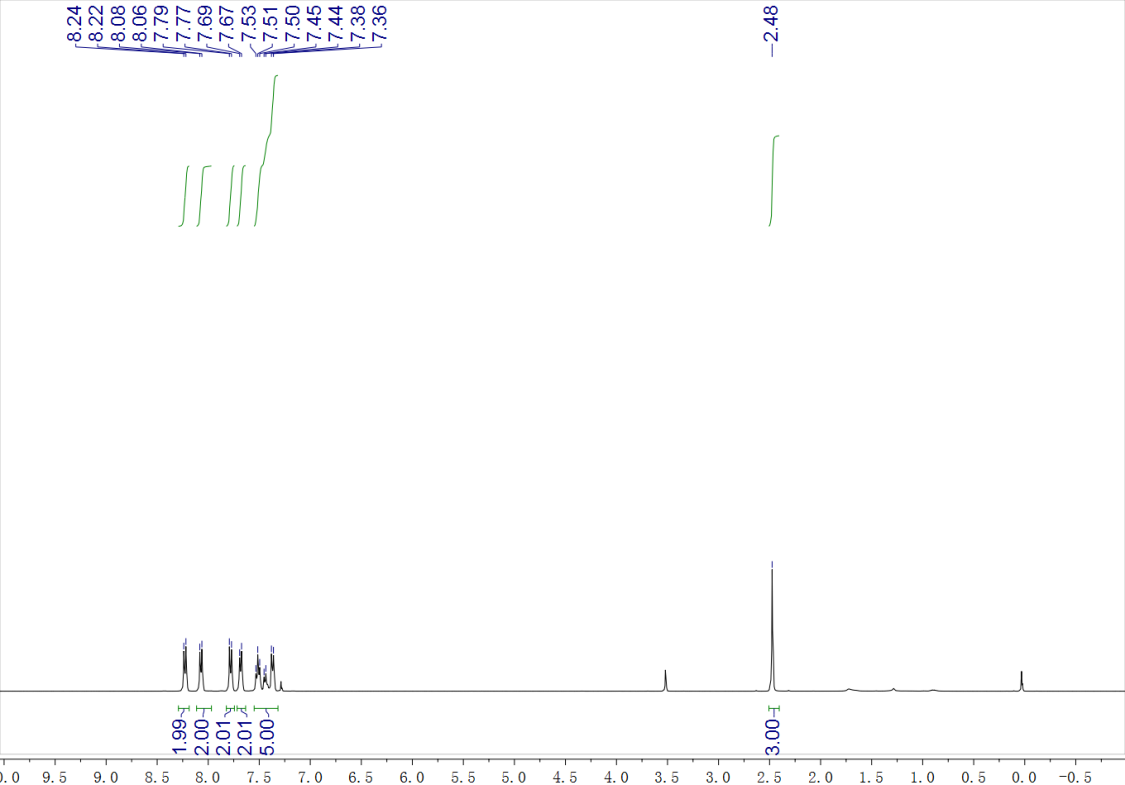


**Figure S59.** ^1^H-NMR spectrum of compound **6f**


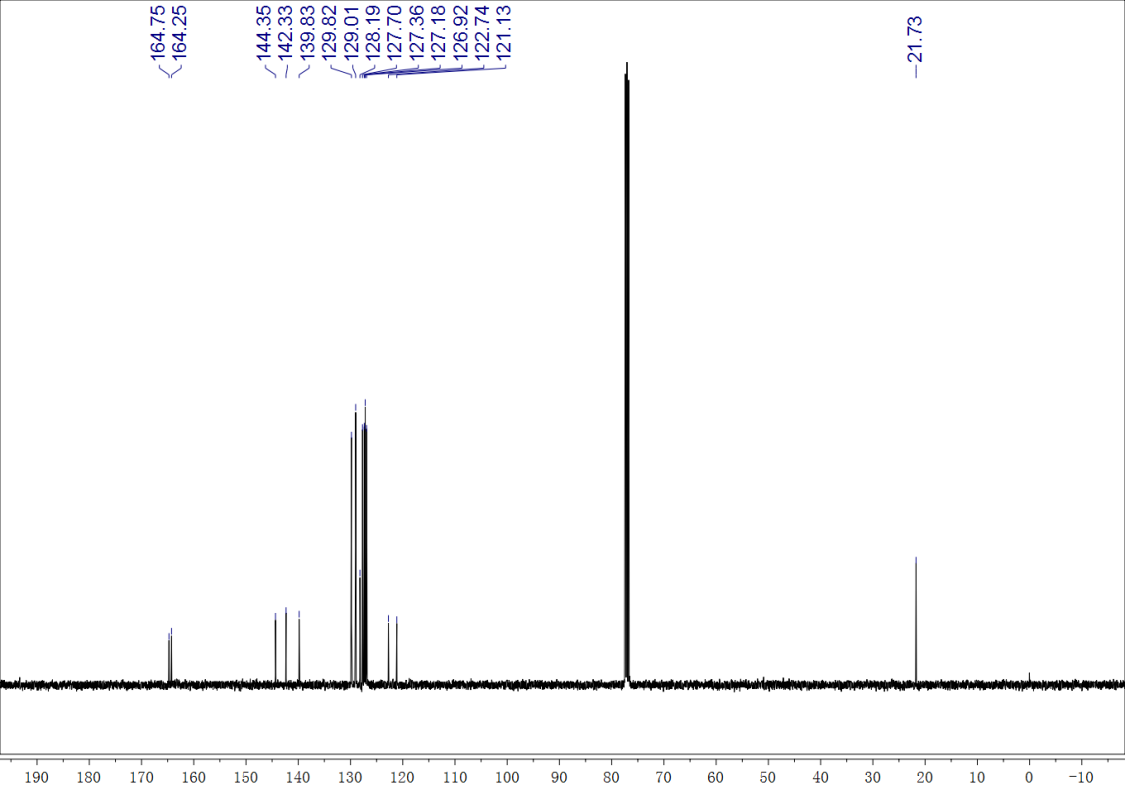


**Figure S60.** ^13^C-NMR spectrum of compound **6f**
